# Heterometallic lanthanide complexes with site-specific binding that enable simultaneous visible and NIR-emission

**DOI:** 10.3389/fchem.2023.1232690

**Published:** 2023-07-31

**Authors:** Matthew E. Thornton, Jake Hemsworth, Sam Hay, Patrick Parkinson, Stephen Faulkner, Louise S. Natrajan

**Affiliations:** ^1^ Department of Chemistry, Faculty of Science and Engineering, The University of Manchester, Manchester, United Kingdom; ^2^ Department of Chemistry, Faculty of Science and Engineering, Manchester Institute of Biotechnology, The University of Manchester, Manchester, United Kingdom; ^3^ Department of Physics and Astronomy and the Photon Science Institute, Faculty of Science and Engineering, The University of Manchester, Manchester, United Kingdom; ^4^ Department of Chemistry, Chemistry Research Laboratory, The University of Oxford, Oxford, United Kingdom

**Keywords:** Lanthanide, luminescence, energy transfer, NIR emission, Macrocycle

## Abstract

Macrocyclic lanthanide complexes have become widely developed due to their distinctive luminescence characteristics and wide range of applications in biological imaging. However, systems with sufficient brightness and metal selectivity can be difficult to produce on a molecular scale. Presented herein is the stepwise introduction of differing lanthanide ions in a bis-DO3A/DTPA scaffold to afford three trinuclear bimetallic [Ln_2_Ln’] lanthanide complexes with site-specific, controlled binding [(Yb_2_Tb), (Eu_2_Tb), (Yb_2_Eu)]. The complexes display simultaneous emission from all Ln^III^ centers across the visible (Tb^III^, Eu^III^) and near infra-red (Yb^III^) spectrum when excited *via* phenyl ligand sensitization at a wide range of temperatures and are consequently of interest for exploiting imaging in the near infra-red II biological window. Analysis of lifetime data over a range of excitation regimes reveals intermetallic communication between Tb^III^ and Eu^III^ centers and further develops the understanding of multimetallic lanthanide complexes.

## 1 Introduction

The photophysical properties of lanthanide ions in the common +III oxidation state (Ln^III^) has become a wide and complex field of research across multiple disciplines and applications, particularly Ln^III^ luminescence for bioimaging (Bünzli and Piguet, 2005; Bünzli, 2010; Monteiro, 2020). Previous works have exploited “windows” of attenuation in the absorption profiles of biological tissues at near-infrared (NIR) wavelengths, allowing deeper penetration of such wavelengths and therefore enhanced imaging (Hemmer et al., 2016; Fan and Zhang, 2019). The Ln^III^ ions are of particular interest due to their characteristic line-like emission across the visible and NIR range, low autofluorescence and photobleaching and high signal-to-noise ratios in comparison to classic organic fluorophores. A caveat to their use is intrinsically poor extinction coefficient as a result of partially-forbidden *f-f* transitions, which is mitigated by sensitization strategies *via* organic chromophores (the antenna effect) or *d-* and *f-*block metal complexes (Faulkner and Pope, 2003; Natrajan et al., 2009). Furthermore, *X*-H (*X* = O, N, C) oscillators are well documented in their ability to vibrationally quench Ln^III^ excited states due to significant overlap between vibrational overtones of these bonds and emissive Ln^III^ energy levels (Doffek et al., 2012).

**FIGURE 1:**
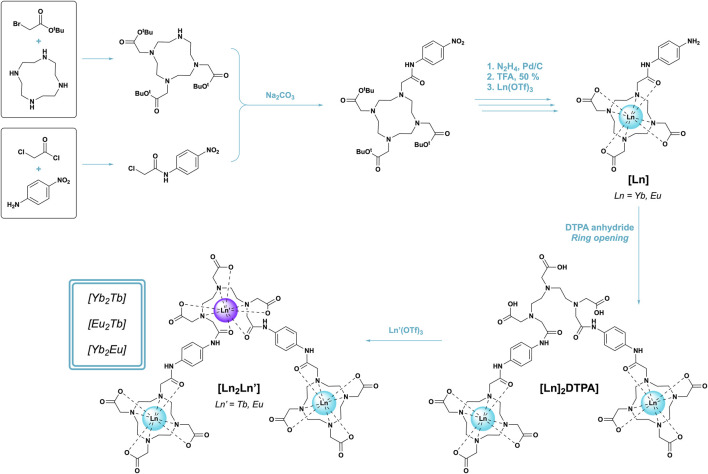
Synthetic scheme of the stepwise approach used in synthesis of [Ln_2_Ln’] trinuclear bimetallics.

This work further develops a previously designed ligand based on a multi-macrocyclic architecture of 1,4,7,10-tetraazacyclododecane-1,4,7-triacetic acid (DO3A) and diethylenetriaminepentaacetic acid (DTPA) (Faulkner and Pope, 2003). Polyamino carboxylate ligands are well known to securely bind lanthanides in a range of environments and have displayed the ability to act as trinuclear heterometallic binding ligands in a site-selective [TbYbTb] arrangement. Herein, we develop the scaffold to include two [LnTbLn] complexes (Ln^III^ = Yb^III^, Eu^III^) and a complementary [YbEuYb] species (Figure 1) and further investigate the photophysical capabilities of the ligand across various temperature windows and excitation regimes. The metal selection results in clear Ln^III^–centred emission across the visible (Tb^III^, Eu^III^) and NIR (Yb^III^) spectrum, which overlaps with both NIR-I (650–950 nm) and NIR-II (1000–1350 nm) imaging windows and therefore invites application in imaging technologies (Foucault-Collet et al., 2013; Jin et al., 2022). The simultaneous response from both Ln^III^ centres has further application as a dual-modal device; the unique magnetic and photophysical behaviour of these metals facilitates use as both MRI contrast agents and optical probes (Rivas et al., 2013; Xu et al., 2013).

Low temperature, solid-state and deuterated media measurements are prioritized to mitigate non-emissive quenching mechanisms. These experiments were also designed to facilitate observation of energy transfer (ET) between metal centers; the relative excited state energies of the chosen metal combinations have been exploited for ET processes in numerous multimetallic Ln^III^ systems (Bispo-Jr et al., 2018; Abad Galán et al., 2021). This work contributes toward the growing library of bimetallic trinuclear Ln^III^ systems that have been studied to elucidate the characteristics of intermetallic communication in discrete molecular complexes (Aboshyan-Sorgho et al., 2012; Zaïm et al., 2014; Tropiano et al., 2015; Maniaki et al., 2023). Finally, the presence of ET in molecular Ln^III^ systems is also useful for imaging applications such as those employing two-photon processes like upconversion (Aboshyan-Sorgho et al., 2011; Nonat et al., 2019).

## 2 Materials and methods

### 2.1 Synthesis and characterization

The overall synthesis of target complexes [Yb_2_Tb], [Eu_2_Tb], and [Yb_2_Eu] was adapted and modified from a previously reported literature procedure (Natrajan et al., 2009). Experimental procedures and characterization are detailed on pages S2-S41.

1,4,7,10-tetraazacyclododecane (cyclen) was purchased from CheMatech and used without further purification. All other reagents and solvents were purchased from Sigma-Aldrich, Fluorochem Ltd. or Apollo Scientific Ltd. and used without further purification. Electrospray +/− (ES-MS) spectra were recorded on a Thermo Orbitrap Exactive Plus mass spectrometer. MALDI-TOF spectra were recorded on a Shimadzu Biotech Axima Confidence mass spectrometer. FT-IR spectra were recorded on a Bruker ALPHA I FT-IR spectrometer. Elemental analysis data were recorder using a Thermo Scientific FlashSmart Elemental Analyzer.

NMR spectra were recorded on a Bruker AVIII HD 500 MHz spectrometer (BBFO inverse probe) in deuterated chloroform, deuterium oxide or deuterated methanol and analyzed using MestReNova 14.1.0. Chemical shifts in parts per million (ppm–δ) are reported relative to residual proton resonances and an internal tetramethylsilane reference. Splitting abbreviations: s: singlet, br. s: broad singlet, d: doublet, dd: doublet of doublets, t: triplet, dt: doublet of triplets, m: multiplet. Blank sections of spectra or those containing solvent resonances are omitted in certain spectra for clarity. Due to complex isomerism between the square and twisted square antiprismatic (SAP ↔ TSAP) forms of multi-macrocyclic cyclen compounds, ^1^H NMR assignments were often achieved *via* correlation with 2D COSY, HSQC and HMBC data where possible (Miller et al., 2010; Tircso et al., 2011). ^1^H NMR data for compounds containing paramagnetic atoms (Yb^III^, Tb^III^, Eu^III^) were processed using a line broadening/apodization factor of 1.5–5 Hz and baseline corrected using a multipoint baseline correction with a Whittaker, cubic spline or segment algorithm. The chemical shift values of the ^1^H resonances in all the lanthanide (III) complexes are reported without assignment due to the complex nature of the paramagnetic NMR assignment of polyaminocarboxylate lanthanide compounds (Sørensen et al., 2017). ^13^C data could not be collected for the same paramagnetic compounds.

Energy minimization of crystal structures was carried out using Avogadro 1.2.0. Compound structures were downloaded as mol2 files from the Cambridge Crystallographic Data Centre (CCDC) and Ln^…^C distances measured directly from crystallographic data without any further modification to the structure. The superimposed Yb^III^—Gd^III^ structure was achieved *via* manual manipulation of each structure to minimize steric clash and bonding of the two structures. Bond angles and lengths were maintained during any new bond formation. The auto optimization tool was then used to minimize the energy of the new structure (UFF force field, steepest descent algorithm). Intermetallic measurements were then conducted on this new, minimzed structure.

### 2.2 Luminescence spectroscopy

Luminescence spectra were recorded on an Edinburgh Instruments FLS1000 Photoluminescence Spectrometer. Solid-state and low temperature spectra were recorded using a cryostat attachment with the sample deposited on a fused silica slide and were the default method of measurement unless stated otherwise. Solution measurements were recorded using a Hellma quartz glass 3.5 mL cuvette with a 1 cm path length and a sample absorbance of 0.1. UV-VIS spectra were recorded using a Mettler-Toledo UV5Bio spectrometer. Samples were excited using a 450 W Xe lamp with a long-pass filter on the detection arm and emission captured by PMT-900 (visible) and PMT-1700 (NIR) detectors. Any direct comparison of spectra used identical settings; excitation/emission monochromator slit widths and post-collection processing were identical for all. Lifetime measurements were collected using a microsecond flash lamp operating at 40 Hz (Tb^III^, Eu^III^ data) or 100 Hz (Yb^III^ data). Plotting, fitting and analysis of data was carried out using Origin 2019b. All data were fitted with exponential decay models starting with the fewest terms (mono-, bi-exponential) until sufficiently good fit residuals were achieved. In particular, 2-component fits were always compared against tri-exponential alternatives and found to better fit the data *via* residual, visual, Chi^2^ and *R* ^2^ analysis.

Shorter–lived lifetimes from Yb^III^ emission require consideration of the instrument response function (IRF) of the excitation source, both of which are on a microsecond timescale. An IRF trace was recorded at 100 Hz in line with the procedure detailed by the instrument manufacturer (Edinburgh Instruments) by matching excitation and detection wavelengths (λ = 280 nm) and recording the decay. This was repeated across different temperatures, both with the sample present and separately using milk powder to provide scatter (in both solid and solution-state). The variation in IRF trace between different variables is minimal. Detector response of the (NIR) PMT-1700 was also considered by recording a pseudo-IRF detecting scattered 2λ light from visible excitation (λ_ex_ = 600 nm, λ_em_ = 1200 nm), to provide a similar result. The convolution tool in Origin was used to generate a decay trace with the appropriate IRF taken into consideration, which was then fitted to an exponential decay profile in the same manner as all other lifetime fits. Fitting of Tb^III^ and Eu^III^ signals used the IRF as a benchmark to ensure fitting parameters were only applied after the instrument response had decayed to background and therefore could not contribute to the resulting lifetime.

## 3 Results

### 3.1 Photophysical properties of the near infra-red-visible emitting complex [Yb_2_Tb]

**FIGURE 2:**
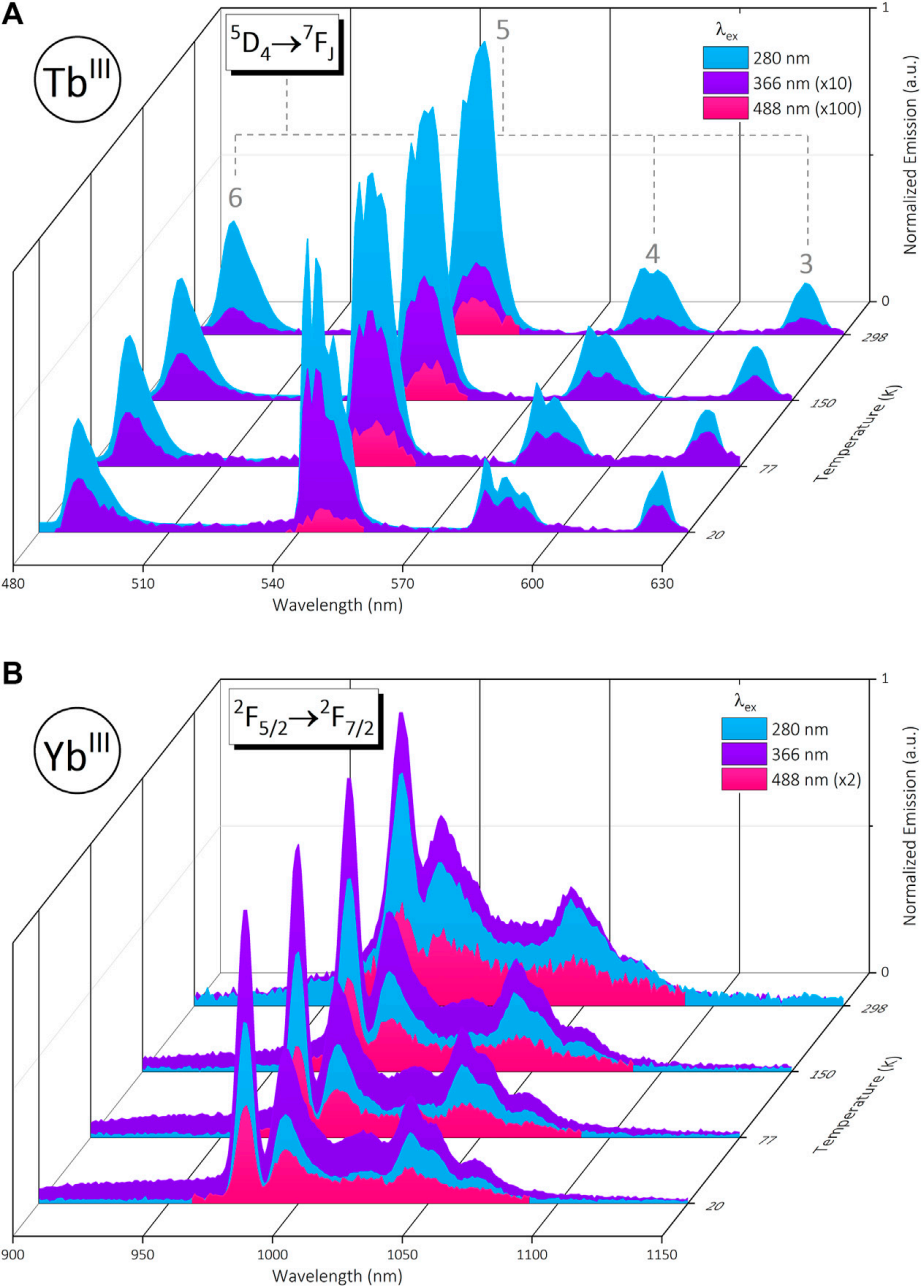
**(A)**: Normalized visible emission of trinuclear bimetallic [Yb_2_Tb] under 280 nm (blue), 366 nm (purple) and 488 nm (pink) excitation in the solid state from 20—298 K. **(B)**: Simultaneous normalized NIR emission from [Yb_2_Tb] under the same conditions. Data are normalized at each temperature relative to the emission maximum (∼545 nm @ λ_ex_ = 280 nm for 2A, ∼980 nm @ λ_ex_ = 366 nm for 2B).

Initial excitation *via* the phenyl linker (λ_ex_ = 280 nm) affords sensitized visible emission from the Tb^III^ metal center ^5^D_4_ → ^7^F_J_ (J = 6, 5, 4, 3) transitions. Additional wavelengths selected to directly probe the ^5^D_3_ (λ_ex_ = 366 nm) and ^5^D_4_ (λ_ex_ = 488 nm) energy levels of Tb^III^ also result in emission, however at a lower intensity due to poor extinction coefficients at these wavelengths. Only the primary ^7^F_5_ transition is visible at λ_ex_ = 488 nm. Solid-state variable temperature spectra at 20, 77, 150, and 298 K highlights an increased intensity and resolution of *m*_*j*_ state crystal field splitting at lower temperatures, most notably in the central 545 nm ^5^D_4_ → ^7^F_5_ transition (Figure 2A, Supplementary Figure S2.1).

Simultaneous NIR Yb^III^ emission from the ^2^F_5/2_ → ^2^F_7/2_ transition (∼980 nm) and the associated 4-fold splitting of the Kramer ground state are present under the same set of excitation regimes across a range of temperatures, with an expected loss in resolution, intensity and broadening of signal at higher temperatures (Figure 2B, Supplementary Figure S2.2). The relationship between emission intensity and excitation wavelength is not concordant between the Yb^III^ and Tb^III^ centers. The signal intensity of the Yb^III^ ^2^F_7/2_ transition increases at λ_ex_ = 366 nm; the intended Tb^III^ ^5^D_3_ state excitation. However, this is not unique to the trinuclear [Yb_2_Tb] species as the mono and bimetallic Yb^III^ precursors [Yb] and [Yb]_2_DTPA display the same behavior (Supplementary Figures S2.3, S2.4). Inspection of solid-state excitation profiles for the series at 980 nm (Supplementary Figure S2.5) show a broad ligand-centered band with a hypsochromic shift across the series from [Yb] (λ_max_ = 331 nm) to [Yb_2_Tb] (λ_max_ = 311 nm) and further again for the Tb^III^ center (λ_em_ = 545 nm, λ_max_ = 296 nm). Solution-state excitation spectra for the series (Supplementary Figure S2.6) identify this primarily as a solid state effect and present a narrowing of each profile (λ_max_ = 285 nm) with a similar ∼15 nm shift for Tb^III^ excitation (λ_max_ = 270 nm). UV-VIS absorption data show minimal change in signal when comparing complexes and resemble solution-state profiles (Supplementary Figure S2.7).

Solid-state lifetime measurements of the primary Tb^III^ emission at 545 nm fit a bi-exponential decay profile across all temperatures and λ_ex_, determined *via* analysis of *R* ^2^ values and fit residuals (Supplementary Figures S2.8–S2.10). For simplicity, we report the global fluorescence lifetime, *τ*_*n*_, of each decay component which is an average across variable λ_ex_ (280, 366, 488 nm) and temperature (20, 77, 150, and 298 K). This value for the two exponents of Tb^III^ decay are 0.14 ± 0.01 ms and 0.70 ± 0.03 ms for τ_1_ and τ_2_ respectively (fitting after the IRF). The relative contribution of each component varies marginally across the variable excitation and temperature series, but the long-lived τ_2_ component remains the most significant (Supplementary Table S2). Calculation of the average lifetime, τ_avg_, considers the variation in percentage contribution from each *τ*_*n*_ component toward an overall average value (Supplemetary equations S1, S2). Factoring in all data across the excitation and temperature range results in τ_avg_ = 0.55 ± 0.03 m for the Tb^III^ center (Table 1).

**TABLE 1:** Solid-state lifetimes of Ln^III^ centers at multiple temperatures from the [Yb] complex series including trinuclear bimetallic [Yb_2_Tb], averaged across variable excitation wavelengths (298 K: dark red, 150 K: light red, 77 K: dark blue, 20 K: light blue). Tb^III^ values are calculated average lifetimes from a bi-exponential fit.

**Lifetime**		**Yb^III^ τ (µs)**			**Tb^III^ τ_avg_ (ms)**
**Complex**		[Yb] (*± 0.98 µs*)	[Yb]_2_DTPA (*± 1.2 µs*)	[Yb_2_Tb] (*± 1.3 µs*)	[Yb_2_Tb] (*± 0.03 ms*)
**Temperature (K)**	298	7.6	10	8.1	0.56
	150	7.7	9.9	8.4	0.57
	77	8.0	10	8.7	0.56
	20	8.5	11	9.2	0.51

The 980 nm–centered Yb^III^ emission fits a mono-exponential decay following convolution with the IRF signal (Supplemetary Figures S2.11—S2.14) and has a global fluorescence lifetime τ = 8.6 ± 0.4 µs across variable λ_ex_ and temperature. This is comparable to lifetime values for Yb^III^ precursors (±0.4 µs); τ [Yb] = 7.9 µs, τ [Yb]_2_DTPA = 10 µs (Table 1).

### 3.2 Emission behavior of the dual-visible emitting complex [Eu_2_Tb]

**FIGURE 3:**
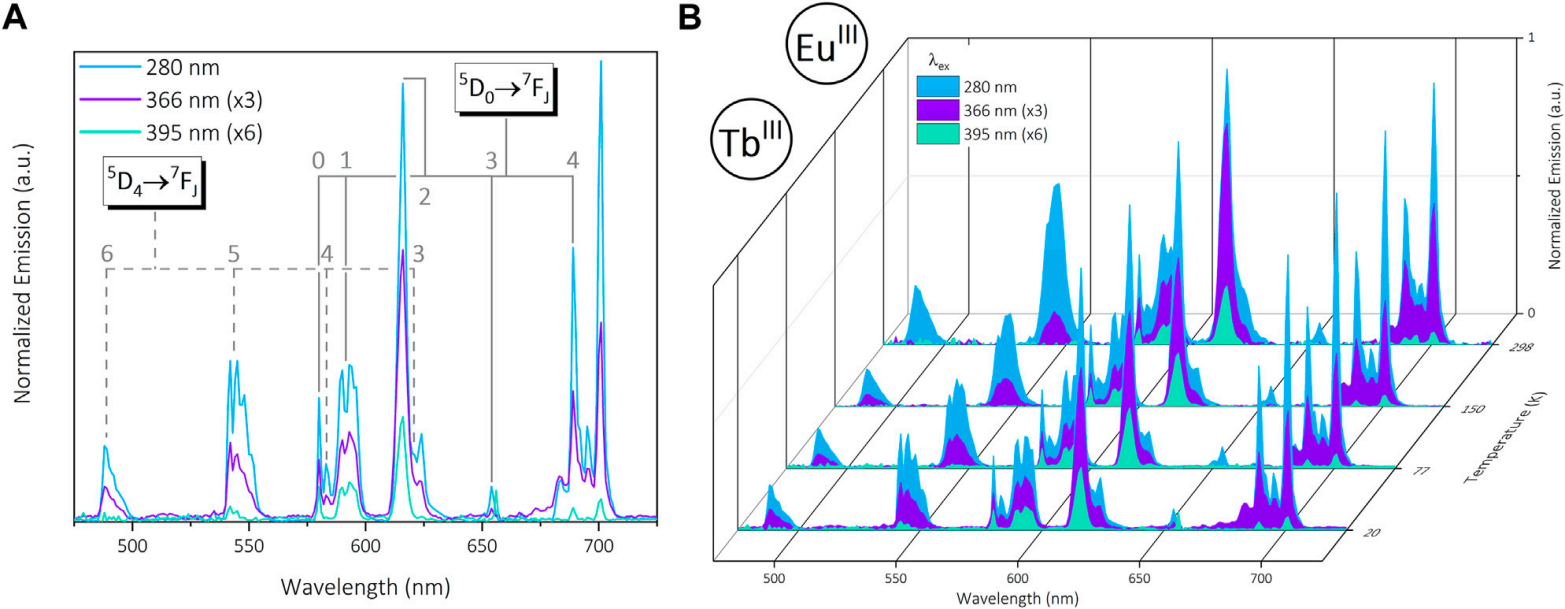
**(A)**: Normalized visible emission of trinuclear bimetallic [Eu_2_Tb] under 280 nm (blue), 366 nm (purple) and 395 nm (green) excitation in the solid state at 20 K. **(B)**: Normalized visible emission from [Eu_2_Tb] at 20—298 K across the same set of excitations. Data are normalized at each temperature relative to the emission maximum (∼701 nm @ λ_ex_ = 280 nm).

Insertion of Eu^III^ into the DO3A binding pocket results in strong visible emission (480—700 nm) from the same ^7^F_J_ Tb^III^ transitions present in [Yb_2_Tb], in addition to the ^5^D_0_ → ^7^F_J_ (J = 0–4) transitions from Eu^III^. As above, excitation of the phenyl linker at λ_ex_ = 280 nm results in simultaneous sensitized emission from the two metal centers (Figure 3A). Direct excitation of the Tb^III^ ^5^D_3_ band (λ_ex_ = 366 nm) again yields emission from both metals proportional to respective excitation profiles. The second direct Tb^III^ excitation (^5^D_4_, λ_ex_ = 488 nm) employed in [Yb_2_Tb] is relatively weak in this complex, meaning no significant emission was observed. Alternatively, direct excitation of the Eu^III^ ^5^L_6_ level (λ_ex_ = 395 nm) can be used to selectively produce Eu^III^–centered emission and minimize Tb^III^ spectral features, namely, the J = 4, 3 transitions (Figure 3A). Variable temperature emission spectra highlight the consistency of this wavelength–selective visible emission in addition to crystal field splitting at lower temperatures, in particular the Eu^III^ ^7^J_1_ and ^7^J_2_ signals (Figure 3B, Supplementary Figure S2.15). The lack of splitting in the ^5^D_0_ → ^7^F_0_ transition is indicative of a singular Eu^III^ environment due to the non-degeneracy of both states, as expected from equivalent [Eu^III^(DO3A)] sites (Binnemans, 2015). Additionally, the hypersensitive ^7^F_2_ transition known to be highly dependent on coordination environment remains consistent throughout. Analysis of λ_ex_ against temperature shows an appreciable change in intensity at 701 nm (Eu^III^ ^7^F_4_ state) when changing from sensitized (λ_ex_ = 280 nm) to direct excitation (λ_ex_ = 395 nm) (Supplementary Figure S2.16). However, the relative intensity of the ^7^F_4_ signal is strong overall which is in agreement with the square antiprismatic geometry expected of macrocyclic species such as DO3A (Binnemans, 2015).

Fitting of the 545 nm Tb^III^ decay results in a bi-exponential fit analogous to the [Yb_2_Tb] species (τ_1_ = 0.11 ± 0.01 ms, τ_2_ = 0.56 ± 0.07 ms). The percentage contribution of each lifetime component is more evenly distributed than in the corresponding [Yb_2_Tb] complex, resulting in a shorter τ_avg_ value of 0.33 ± 0.02 ms (Table 2). However, comparison of the longer-lived τ_2_ component in both Tb^III^-containing complexes highlights similarity between the two (Supplementary Table S3).

**TABLE 2:** Solid-state lifetimes of Ln^III^ centers at multiple temperatures from the [Eu] complex series, averaged across variable excitation wavelengths (298 K: dark red, 150 K: light red, 77 K: dark blue, 20 K: light blue). Both Eu^III^ and Tb^III^ values are calculated average lifetimes from a bi-exponential fit.

**Lifetime**		**Eu^III^ τ_avg_ (ms)**			**Tb^III^ τ_avg_ (ms)**
**Complex**		[Eu] (*± 0.05 ms*)	[Eu]_2_DTPA (*± 0.03 ms*)	[Eu_2_Tb] (*± 0.02 ms*)	[Eu_2_Tb] (*± 0.02 ms*)
**Temperature (K)**	298	0.58	0.76	0.15	0.23
	150	1.1	0.88	0.21	0.35
	77	1.2	0.88	0.23	0.34
	20	1.2	0.89	0.24	0.38

The Eu^III^ lifetimes were measured by fitting the decay of the primary 615 nm emission (^5^D_0_ → ^7^F_2_) at various temperatures and fit with a bi-exponential equation also (Supplementary Figures S2.17—S2.19) (τ_1_ = 82 ± 5 µs, τ_2_ = 0.36 ± 0.04 ms). The contribution of each component toward τ_avg_ is largely independent of λ_ex_ and differs from Tb^III^ bi-exponential fits as the longer-lived τ_2_ component no longer dominates (Supplementary Table S4). Each *τ*_*n*_ component has appreciable significance, yielding a τ_avg_ value of 0.21 ± 0.02 ms. Comparison of component-weighted τ_avg_ Eu^III^ lifetimes from [Eu] and [Eu]_2_DTPA shows they are longer across the series when compared to the final [Eu_2_Tb] bimetallic compound (τ_avg_ = 1.0 ± 0.3 ms and 0.85 ± 0.06 ms respectively, Table 2).

Comparison of Eu^III^ precursors [Eu] and [Eu]_2_DTPA with the trinuclear target species [Eu_2_Tb] highlights significant differences in solid-state excitation profiles arising from 615 nm emission (Supplementary Figure S2.20). The DO3A complex [Eu] displays strong absorption bands corresponding to ^5^D_4_ ← ^7^F_0_ (362 nm), ^5^D_4_ ← ^7^F_1_ (375 nm), ^5^L_6_ ← ^7^F_0_ (395 nm), ^5^L_6_ ← ^7^F_1_ (400 nm) and ^5^D_2_ ← ^7^F_0_ (465 nm) Eu^III^ transitions. Phenyl ligand absorption (λ_ex_ max ≈280 nm) begins to dominate in the following [Eu]_2_DTPA compound, coupled with the loss of the ^5^D_4_ band at 375 nm. [Eu_2_Tb] follows the trend with increased ligand-centered absorption and further absence of the other ^5^D_4_ feature at 362 nm; Eu^III^ series spectra exhibit general solid-state broadening analogous to [Yb_2_Tb]. Solution-state excitation scans in D_2_O are ligand-centered across the series, with a marginal shift between samples (Δλ_max_ = 20 nm) and a minor ^5^L_6_ feature at 395 nm (Supplementary Figure S2.21) and are in agreement with UV-VIS spectra (Supplementary Figure S2.22).

### 3.3 Photophysical properties of the near infra-red-visible emitting complex [Yb_2_Eu]

**FIGURE 4:**
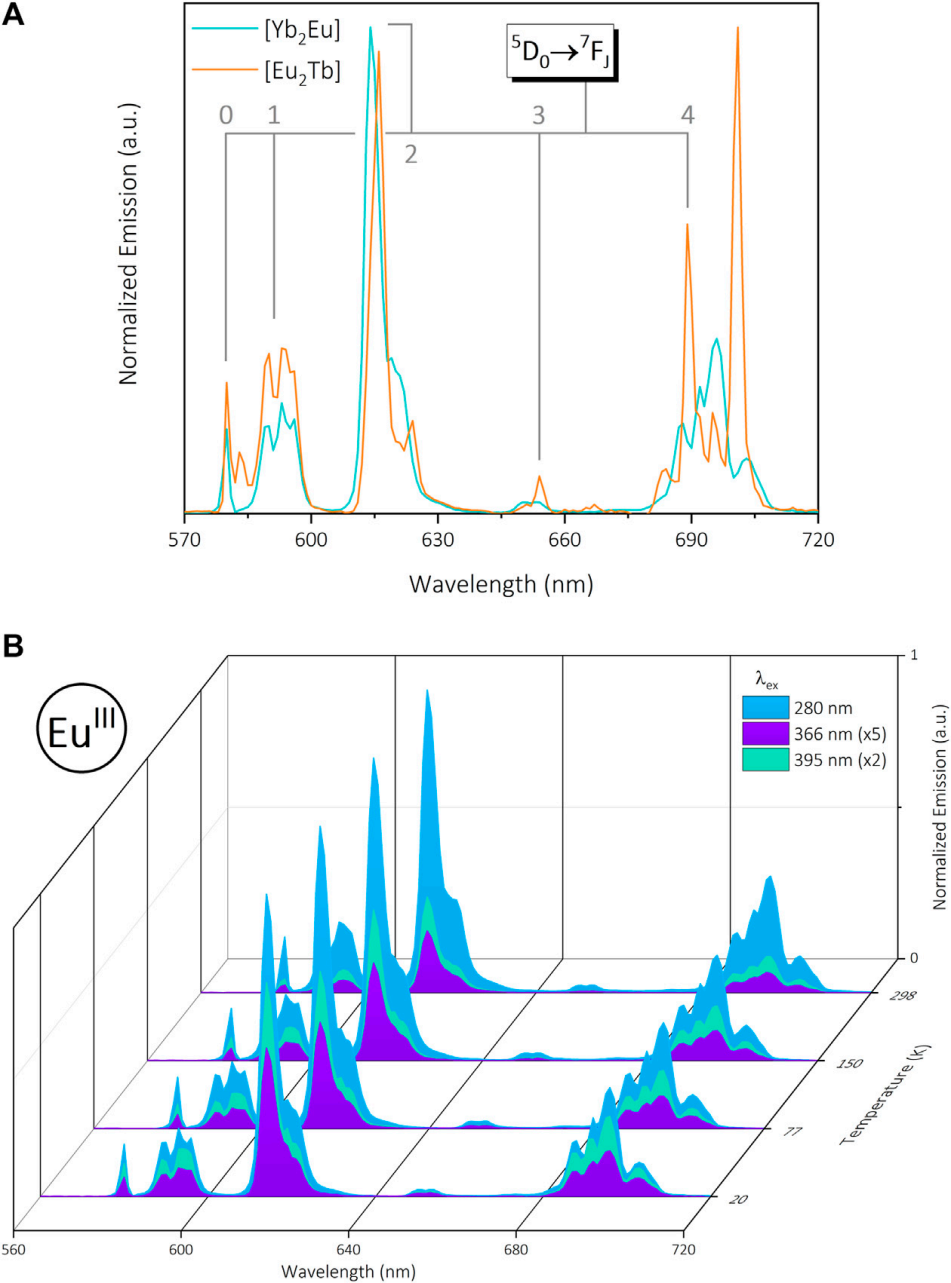
**(A)**: Normalized visible emission of trinuclear bimetallic [Yb_2_Eu] (green) and [Eu_2_Tb] (orange) under 280 nm excitation in the solid state at 20 K. Data are normalized for each complex relative to the emission maximum. **(B)**: Normalized visible emission from [Yb_2_Eu] at 20—298 K under 280 nm (blue), 366 nm (purple) and 395 nm (green) excitation in the solid state (normalized to ∼615 nm @ λ_ex_ = 280 nm).

Synthesis of the {Yb(DO3A)}_2_-{Eu (DTPA)} complex [Yb_2_Eu] results in simultaneous NIR and visible emission from the two metals (λ_ex_ = 280, 366 and 395 nm) analogous to [Yb_2_Tb] and [Eu_2_Tb], with an expected decrease in emission intensity at higher temperatures (Supplementary Figures S2.23, S2.24). Excitation at 366 nm was investigated despite the absence of Tb^III^ to generate results comparable with other data sets. The Eu^III^ ^7^F_2_ transition displays a minor spectral shift, and the ^7^F_4_ presents a change in splitting pattern compared to [Eu_2_Tb], both of which are sensitive to coordination environment and suggest Eu^III^ is selectively bound in the DTPA site (Figure 4A). The Eu^III^ center exhibits a greater response at λ_ex_ = 395 nm compared to [Eu_2_Tb], which is consistent across a range of temperatures (Figure 4B). Solid-state excitation spectra (λ_em_ = 615 nm) show a large ligand-centered signal in addition to distinct Eu^III^ bands analogous to [Eu] and rationalize this behavior (Supplementary Figure S2.25). Solution-state excitation spectra of [Yb_2_Eu] present ^5^D_4_ ← ^7^F_0_, ^7^F_1_ Eu^III^ excitation signals which are absent in solution measurements of the other Eu^III^ complexes (Supplementary Figure S2.26).

The Yb^III^ emission intensity from the ^2^F_5/2_ → ^2^F_7/2_ transition shows an increased temperature-wavelength relationship compared to other Yb^III^ complexes but maintains maximum emission at λ = 366 nm (Supplementary Figure S2.27). At T = 298 K both ligand and Eu^III^ excitation (λ_ex_ = 280 nm and 395 nm, respectively) result in similar emission intensity, however the latter begins to dominate at lower temperatures (T ≤ 150 K). Solid and solution-state excitation spectra at 980 nm exhibit the same relationship as [Yb_2_Tb] with a ∼40 nm blue shift between the two metal centers and UV-VIS spectra is comparable to other Eu^III^ species (Supplementary Figure S2.28).

Lifetime data for Eu^III^ emission in [Yb_2_Eu] is similar to previous samples with τ_1_ = 0.17 ± 0.02 ms, τ_2_ = 0.74 ± 0.03 ms across the temperature and excitation range. The individual component contribution reflects the previous DTPA-bound metal (Tb^III^ [Yb_2_Tb]), with a clear dominance of the long-lived τ_2_ decay (Supplementary Table S5) which gives rise to a longer τ_avg_ value of 0.63 ± 0.09 ms. Yb^III^ lifetimes remain on the same order of magnitude as previous measurements; τ = 9.8 ± 0.6 µs across variable λ_ex_ and temperature. A summary of temperature-dependent lifetime data for each Ln^III^ across the trinuclear bimetallic series is presented in Table 3 and highlights the general observation of longer lifetimes at lower temperatures.

**TABLE 3:** Solid-state lifetimes of Ln^III^ centers at multiple temperatures from the [Ln_2_Ln’] complex series, averaged across variable excitation wavelengths ([Yb_2_Tb] = orange, [Eu_2_Tb] = green, [Yb_2_Eu] = blue). Both Eu^III^ and Tb^III^ values are calculated average lifetimes from a bi-exponential fit.

**Lifetime**		**Yb^III^ τ (µs)**		**Tb^III^ τ_avg_ (ms)**		**Eu^III^ τ_avg_ (ms)**	
**Complex**		$\left[{\mathrm{Yb}}_{2}\mathrm{Tb}\right]$	$\left[{\mathrm{Yb}}_{2}\mathrm{Eu}\right]$	$\left[{\mathrm{Yb}}_{2}\mathrm{Tb}\right]$	$\left[{\mathrm{Eu}}_{2}\mathrm{Tb}\right]$	$\left[{\mathrm{Eu}}_{2}\mathrm{Tb}\right]$	$\left[{\mathrm{Yb}}_{2}\mathrm{Eu}\right]$
**Temperature (K)**	**298**	8.1	9.1	0.56	0.23	0.15	0.60
	**150**	8.4	9.7	0.57	0.35	0.21	0.65
	**77**	8.7	10	0.56	0.34	0.23	0.66
	**20**	9.2	11	0.51	0.38	0.24	0.62
**Global τ**		**8.6** *± 0.4*	**9.8** *± 0.6*	**0.55** *± 0.03*	**0.33** *± 0.06*	**0.21** *± 0.02*	**0.63** *± 0.09*

### 3.4 Solution-state measurements

Solution-state measurements in water and D_2_O result in lifetimes with mono-exponential decay profiles for all metals; the shorter-lived τ_1_ component in the bi-exponential Tb^III^ and Eu^III^ solids are not present. Solution measurements are likely probing an average lifetime arising from minor changes in hydration state and exchange between the square and twisted square antiprismatic (SAP ↔ TSAP) isomers of the DO3A macrocycles (Miller et al., 2010; Tircso et al., 2011; Nielsen and Sørensen, 2019). Consequently, solution experiments exhibit significantly longer lifetimes on average, with significant gains in deuterated solvent due to reduced energetic overlap of *X*–D oscillators with emissive Ln^III^ states (Supplementary Figures S2.29—S2.31) (Doffek et al., 2012). The number of bound solvent molecules can be calculated *via* the inner sphere hydration parameter, *q*, using a modified Horrocks equation (Supplementary Equation S3, Supplementary Table S1) (Beeby et al., 1999). This calculation takes into account the degree of vibrational quenching by proximate *X*-H (*X* = C, N, O) groups for each metal; O-H oscillators contribute significantly for all three, however the significance of N-H groups is relevant for Eu^III^ only and is negligible in magnitude for Tb^III^ and Yb^III^ systems. Whilst C-H quenching arising from the acetate methylene and DO3A ring groups has a considerable influence on the luminescent lifetimes of near infra-red emitting lanthanides, in the case of Yb^III^ this contribution is small when compared to closely diffusing O-H oscillators and mostly unobserved, due to the long *X*-H … Ln^III^ distances of these species and a 1/r^6^ dependence for quenching *via* energy transfer. This enables the estimation of *q* by a modified Horrock’s equation (Beeby et al., 1999).

Comparison of the luminescence decay in water and D_2_O (Table 4) shows *q* values change depending on both metal choice and binding environment, with the lowest arising from DO3A-bound Yb^III^ in [Yb_2_Tb] (*q* = 0.21) and [Yb_2_Eu] (*q* = 0.29). The small size of Yb^III^ due to the lanthanide contraction and availability of hard Lewis basic N- and O- donors in the octadentate DO3A precludes access to nearby solvent molecules (Faulkner and Pope, 2003). In complex [Yb_2_Tb], the neighboring DTPA-bound Tb^III^ is more readily accessible due to a larger ionic radius and reduction in kinetic stability of the DTPA binding pocket, resulting in ∼1 bound water molecule (Idée et al., 2009; Sørensen and Faulkner, 2018). Similarly, in the complex [Eu_2_Tb], *q* = 1.0 for the DO3A Eu^III^ center as a result of the slightly larger 9 coordinate ionic radius (Sastri et al., 2003) which competes with the steric bulk of the DO3A macrocycle to allow inclusion of 1 solvent donor. In the case of the Tb^III^(DTPA) centre in [Eu_2_Tb], an apparent *q* value of 2.4 is determined. However, in the case of substantial energy transfer from the assumedly ^5^D_4_ excited state of Tb^III^ to the ^5^D_0_ state of Eu^III^ occuring, phonon assisted energy transfer processes through the O-H vibrational manifold are in direct competition with those that act to quench the ^5^D_4_ → ^7^F_J_ transitions. Given that both of these quenching pathways will possess different rate constants, it follows that Horrocks equation is no longer appropriate (Beeby et al., 1999). Analysis of lifetime data for the analogous [Yb_2_Eu] complex indicates that any competitive intermolecular energy transfer from the ^5^D_0_ Eu^III^ excited state to the ^7^F_5/2_ excited state of Yb^III^ is inconsequential, and *q* of Eu^III^ bound in the DTPA coordination pocket is calculated as 1.1 as expected.

**TABLE 4:** Solution-state lifetimes of Ln^III^ centers in [Ln_2_Ln’] complexes and associated *q* values for each, at 298 K (λ_ex_ = 280 nm, λ_em_ = 980 nm (Yb^III^), 545 nm (Tb^III^), 615 nm (Eu^III^).

**Complex**	**[Yb_2_Tb]**		**[Eu_2_Tb]**		**[Yb_2_Eu]**	
**Metal**	**Yb^III^**	**Tb^III^**	**Eu^III^**	**Tb^III^**	**Yb^III^**	**Eu^III^**
$\tau_{H_{2}O}$ **(ms)**	0.0019	1.7	0.60	1.0	0.0016	0.58
$\tau_{D_{2}O}$ **(ms)**	0.0078	3.0	1.9	2.4	0.0080	2.3
***q***	**0.2**	**1.1**	**1.0**	**-**	**0.3**	**1.1**

## 4 Discussion

**FIGURE 5:**
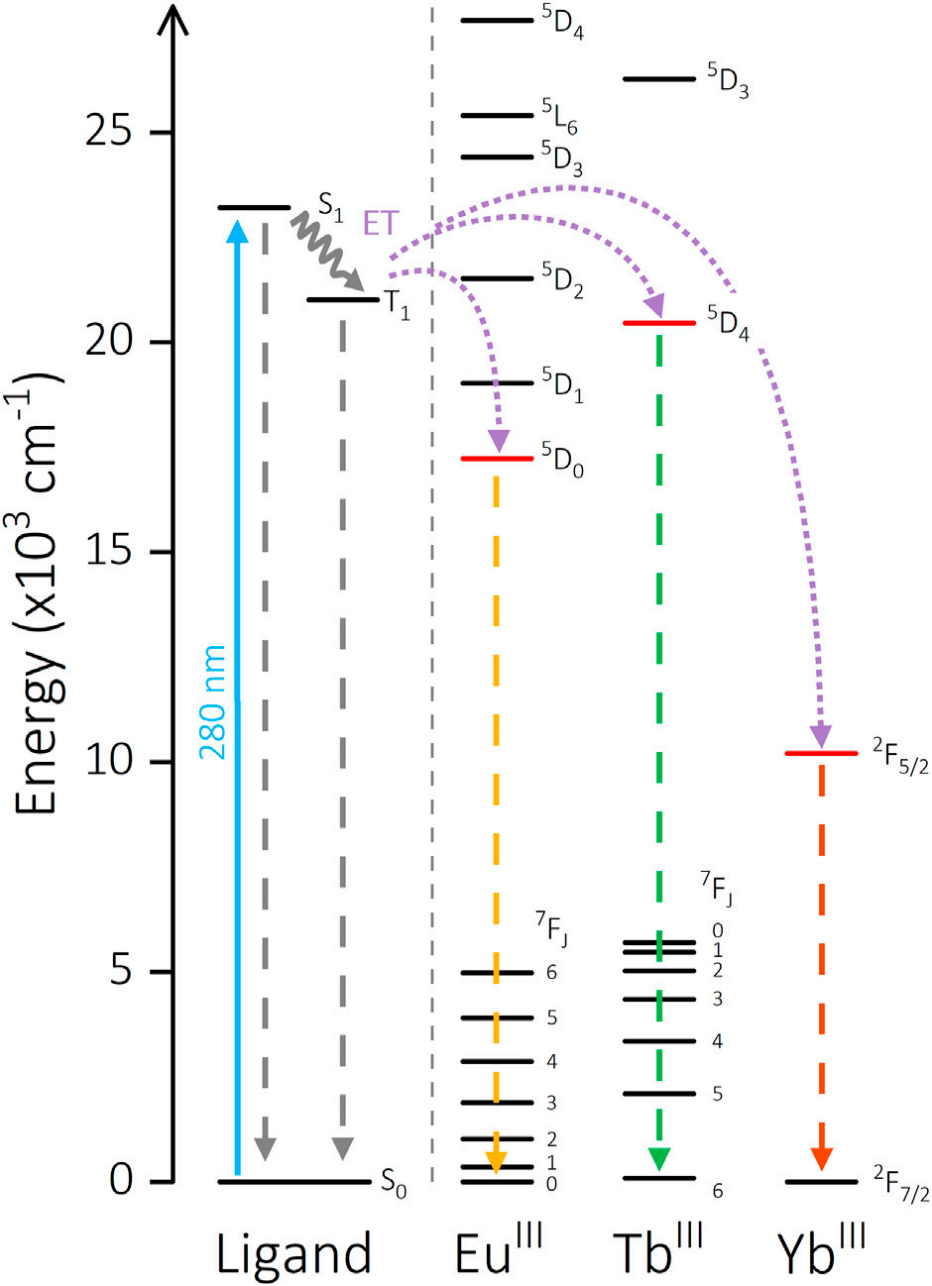
Energy level diagram showing the sensitization pathways of [Ln_2_Ln’] complexes. Solid arrow: excitation, waved arrow: non-radiative decay, dashed arrow: emission, dotted arrow: energy transfer pathway. Ligand energy levels are calculated from [Gd] measurements. Eu^III^, Tb^III^, and Yb^III^ energies are taken from literature for comparison (Carnall et al., 1968a; Carnall et al., 1968b; Sastri et al., 2003).

The potential of this molecular scaffold to facilitate energy transfer between two bound Ln^III^ ions was a key factor in both the ligand and experimental design. Measurement of the Gd-(DO3A)-aminophenyl acetamide complex [Gd] (analogous to [Yb] and [Eu]) facilitates characterization of the triplet state T_1_ of the phenyl linker. The primary Gd ^6^P_7/2_ state is too high in energy to be sensitized, therefore emission of [Gd] is entirely ligand-centered. Solid-state measurements at 298 K display broad fluorescence (λ_em_ = 431 nm), while phosphorescence from the triplet state is observed at 20 K (λ_em_ = 476 nm, Supplementary Figure S2.32). Crucially, this is high enough in energy to sensitize Tb^III^, Eu^III^ and Yb^III^ emissive states (T_1_ = 21,008 cm^-1^, Tb^III^ ^5^D_4_ = 20,453 cm^-1^, Eu^III^ ^5^D_0_ = 17,227 cm^-1^, Yb^III^ ^2^F_7/2_ = 2924 cm^-1^) (Carnall et al., 1968b; 1968a; Sastri et al., 2003) (Figure 5). Additionally, the relative energies and stoichiometry of potential donor and acceptor states (2:1 acceptor:donor ratio) of the metal centers are conducive toward intermetallic ET pathways in [Yb_2_Tb] (Tb^III^ ^5^D_4_ → Yb^III^ ^2^F_5/2_) and [Eu_2_Tb] (Tb^III^ ^5^D_4_ → Eu^III 5^D_0_). The potential [Yb_2_Eu] pathway (Eu^III^ ^5^D_0_ → Yb^III^ ^2^F_5/2_) requires too great an energy mismatch to be a competing process, even when considering phonon assistance *via* the vibrational manifold of O-H oscillators, as is possibly the case in the Tb^III^ → Yb^III^ systems.

Existence of energy transfer between bound metals is often strongly correlated with changes in the luminescence lifetime of excited states, where an energetic pathway from the donor excited state to an acceptor results in an overall reduction in lifetime in the former. An observed reduction in lifetime is apparent as temperature increases for each metal site due to increased *X*-H (*X* = C, N, O) vibrations and therefore quenching of excited states. Recorded lifetimes for the potential Tb^III^ ^5^D_4_ donor state are largely consistent across a range of excitation bands. There is however a marked decrease in Tb^III^ τ_avg_ values between target complexes [Yb_2_Tb] and [Eu_2_Tb] (0.55 ms vs. 0.33 ms). There is also a significant reduction of Eu^III^ τ_avg_ in [Eu_2_Tb] compared to the Eu^III^ complex series (0.64 ms, ∼75%) which is not present in the analogous Yb^III^(DO3A) acceptor series, suggesting the existence of an relaxation pathway present in [Eu_2_Tb] that is absent in [Yb_2_Tb]. Furthermore, Table 3 indicates a 48% decrease in Eu^III^ τ_avg_ from [Yb_2_Eu] to [Eu_2_Tb] and supports evidence toward a Eu^III^-Tb^III^ interaction. This is illustrated more clearly when considering the observed rate constants *k*_obs_ (1/τ_avg_), for Eu^III^ emission in various systems. There is an increase in *k*_obs_ upon addition of Tb^III^ compared to the Eu^III^–only complex series ([Eu] *k*_obs_ = 1000 µs^-1^ [Eu]_2_DTPA *k*_obs_ = 1180 µs^-1^ [Eu_2_Tb] *k*_obs_ = 4762 µs^-1^). Estimation of the potential energy transfer rate constant *k*_*ET*_ can be calculated as the difference between the rate radiative decay from Eu^III^ in the presence and absence of a Tb^III^ transfer partner (*k*_*ET*_ = *k*_*EuTb*_–*k*_*Eu*_), using data from [Eu_2_Tb] and [Eu]_2_DTPA, respectively. Calculation with τ_avg_ values for these compounds yield a value of *k*_*ET*_ = 3582 µs^-1^. Analogous changes in lifetime are not observed in complexes where either metal is paired with Yb^III^. Additionally, DO3A-bound Yb^III^ and Eu^III^ complexes show minimal variations in lifetime when paired with a spectroscopically silent Lu^III^ in the central DTPA pocket ([Yb_2_Lu] [Eu_2_Lu], Supplementary Table S6).

**FIGURE 6:**
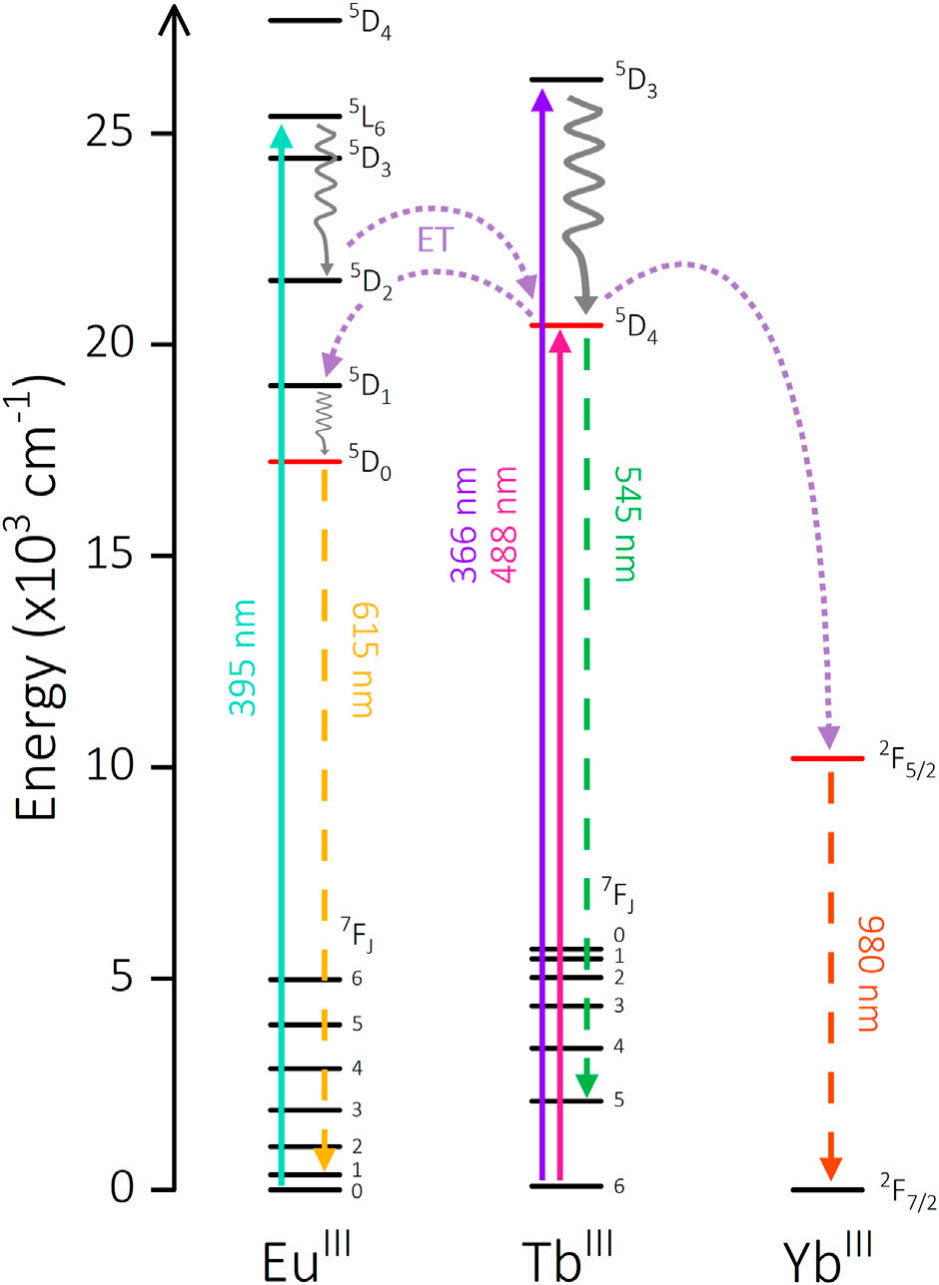
Energy level diagram showing potential intermetallic energy transfer mechanisms in [Ln_2_Ln’] complexes. Solid arrow: excitation, waved arrow: non-radiative decay, dashed arrow: emission, dotted arrow: energy transfer pathway.

Indeed, the ^5^D_J_ energy levels of Tb^III^ (J = 4) and Eu^III^ (J = 2, 1, 0) exhibit appreciable energetic overlap. The 395 nm excitation feature in [Eu_2_Tb] populates a Eu^III^ ^5^L_6_ state which is energetically higher than the Tb^III^ ^5^D_4_ level (ΔE = 4947 cm^-1^) and can populate the latter *via* non-radiative decay to ^5^D_2_ and subsequent ET (Figure 6) (Bispo et al., 2018). Additionally, there is the possibility of phonon-assisted ET facilitated by proximate *X*-H oscillators. The manifold provided by vibrational overtones of O-H and O-D oscillators can provide an alternate energetic pathway to populate ^7^F_J_ states of Tb^III^ from direct Eu^III^ ^5^L_6_ population. Irrespective of these pathways, the emissive states of both Tb^III^ and Eu^III^ are higher in energy than the ^7^F_J_ states of either metal (Supplementary Figure S2.33) and have been reported to communicate in similar systems (Zaïm et al., 2014). The simultaneous reduction in lifetime of both Eu^III^ and Tb^III^ is exclusive to [Eu_2_Tb] and is not present in the parent complexes. The ability of each to act as both donor and acceptor when paired together appears to facilitate a series of ET and possibly back energy transfer (BET) processes between the metal centers.

Despite evidence of solvent interactions and consequent vibrational quenching, solution lifetimes are comparable or often longer than those in the solid state. The additional components in solid-state data likely arise from a combination of SAP ↔ TSAP isomerism and intermolecular quenching between emissive centers in two or more molecules. Solid-state quenching effects appear to play a significant role in the ability of the complex to facilitate energy transfer, as preliminary evidence of Tb^III^-sensitized Yb^III^ emission has previously been reported in solution (Faulkner and Pope, 2003). However, intermetallic distance is another contributing factor and is especially pertinent in solution-state measurements (Sørensen et al., 2017). Single crystal X-ray diffraction structures reported for aminophenyl trifluoromethyl Yb^III^(DO3A) (CCDC ID: EGOWUV) and bis-aminocarboxyphenyl Gd^III^(DTPA) (CCDC ID: QEZGIM) complexes with analogous phenyl moieties allow approximate calculation of these distances *via* superimposition and preliminary energy minimization. Crystal structures were used without modification and manipulated into a feasible geometry before undergoing optimization in Avogadro to yield an average Yb^III^–Gd^III^ distance of 11.9 Å (Supplementary Figure S2.34) (Dutta et al., 2006; Pujales-Paradela et al., 2019). Examples of multimetallic architectures that exhibit intermetallic energy transfer often report shorter distances (≤10 Å) (Natrajan et al., 2009; Maniaki et al., 2023). The significant r^−6^ distance dependence of dipolar ET processes and the innate flexibility of molecular lanthanide systems both act to quench any potential intermetallic communication.

## 5 Conclusion

A series of three trinuclear bimetallic lanthanide complexes have been synthesized with selective introduction of metals into specific DO3A and DTPA binding sites. All three species exhibit strong sensitized emission when excited *via* a phenyl linker and represent a broad spectral range, from visible to NIR depending on the metal combination selected. Extensive photophysical measurements investigating the effects of temperature and excitation wavelength revealed communication between Eu^III^ and Tb^III^ centers when closely bound within the same complex, highlighting the potential for energy transfer between metals. The absence of evidence of intermetallic communication in the Yb^III^ heterometallics emphasizes the impact of solid-state quenching and solution-state molecular motion on multimetallic lanthanide scaffolds that could facilitate energy transfer. Further work on optimizing the distance between metal centers and varying Ln^III^ selection will be investigated in the future, with the aim of observing energy transfer and therefore accessing more intricate photophysics and applications.

## Data Availability

The datasets presented in this study can be found in online repositories. The names of the repository/repositories and accession number(s) can be found in the article/Supplementary Material.

## References

[B1] Abad Galán L. Aguilà D. Guyot Y. Velasco V. Roubeau O. Teat S. J. (2021). Accessing lanthanide-to-lanthanide energy transfer in a family of site-resolved [LnIIILnIII′] heterodimetallic complexes. Chem. Eur. J. 27, 7288–7299. 10.1002/chem.202005327 33448501

[B2] Aboshyan-Sorgho L. Besnard C. Pattison P. Kittilstved K. R. Aebischer A. Bünzli J.-C. G. (2011). Near-Infrared→Visible light upconversion in a molecular trinuclear d–f–d complex. Angew. Chem. Int. Ed. 50, 4108–4112. 10.1002/anie.201100095 21462286

[B3] Aboshyan-Sorgho L. Nozary H. Aebischer A. Bu J.-C. G. Morgantini P.-Y. Kittilstved K. R. (2012). Optimizing millisecond time scale near-infrared emission in polynuclear chrome(III)−Lanthanide(III) complexes. J. Am. Chem. Soc. 134 (30), 12675–12684. 10.1021/ja304009b 22725838

[B4] Beeby A. Clarkson I. M. Dickins R. S. Faulkner S. Parker D. Royle L. (1999). Non-radiative deactivation of the excited states of europium, terbium and ytterbium complexes by proximate energy-matched OH, NH and CH oscillators: An improved luminescence method for establishing solution hydration states. J. Chem. Soc. Perkin Trans. 2 (3), 493–504. 10.1039/a808692c

[B5] Binnemans K. (2015). Interpretation of europium(III) spectra. Coord. Chem. Rev. 295, 1–45. 10.1016/j.ccr.2015.02.015

[B6] Bispo A. G. Lima S. A. M. Pires A. M. (2018). Energy transfer between terbium and europium ions in barium orthosilicate phosphors obtained from sol-gel route. J. Lumin. 199, 372–378. 10.1016/j.jlumin.2018.03.057

[B7] Bünzli J. C. G. (2010). Lanthanide luminescence for biomedical analyses and imaging. Chem. Rev. 110 (5), 2729–2755. 10.1021/cr900362e 20151630

[B8] Bünzli J. C. G. Piguet C. (2005). Taking advantage of luminescent lanthanide ions. Chem. Soc. Rev. 34, 1048–1077. 10.1039/b406082m 16284671

[B9] Carnall W. T. Fields P. R. Rajnak K. (1968a). Electronic energy levels of the trivalent lanthanide aquo ions. III. Tb^3+^ . J. Chem. Phys. 49, 4447–4449. 10.1063/1.1669895

[B10] Carnall W. T. Fields P. R. Rajnak K. (1968b). Electronic energy levels of the trivalent lanthanide aquo ions. IV. Eu^3+^ . J. Chem. Phys. 49, 4450–4455. 10.1063/1.1669896

[B11] Doffek C. Alzakhem N. Bischof C. Wahsner J. Güden-Silber T. Lügger J. (2012). Understanding the quenching effects of aromatic C-H- and C-D-oscillators in near-IR lanthanoid luminescence. J. Am. Chem. Soc. 134, 16413–16423. 10.1021/ja307339f 23009210

[B12] Dutta S. Kim S. K. Eun J. L. Kim T. J. Kang D. S. Chang Y. (2006). Synthesis and magnetic relaxation properties of paramagnetic Gd-complexes of new DTPA-bis-amides. The X-ray crystal structure of [Gd(L)(H_2_O)]·3H_2_O (L = DTPA-bis(4-carboxylicphenyl)amide). Bull. Korean Chem. Soc. 27, 1038–1042. 10.5012/bkcs.2006.27.7.1038

[B13] Fan Y. Zhang F. (2019). A new generation of NIR-II probes: Lanthanide-based nanocrystals for bioimaging and biosensing. Adv. Opt. Mater. 7, 1801417. 10.1002/adom.201801417

[B14] Faulkner S. Pope S. J. A. (2003). Lanthanide-sensitized lanthanide luminescence: Terbium-sensitized ytterbium luminescence in a trinuclear complex. J. Am. Chem. Soc. 125, 10526–10527. 10.1021/ja035634v 12940728

[B15] Foucault-Collet A. Gogick K. A. White K. A. Villette S. Pallier A. Collet G. (2013). Lanthanide near infrared imaging in living cells with Yb3+ nano metal organic frameworks. Proc. Natl. Acad. Sci. U. S. A. 110, 17199–17204. 10.1073/pnas.1305910110 24108356 PMC3808657

[B16] Hemmer E. Benayas A. Légaré F. Vetrone F. (2016). Exploiting the biological windows: Current perspectives on fluorescent bioprobes emitting above 1000 nm. Nanoscale Horiz. 1, 168–184. 10.1039/c5nh00073d 32260620

[B17] Idée J. M. Port M. Robic C. Medina C. Sabatou M. Corot C. (2009). Role of thermodynamic and kinetic parameters in gadolinium chelate stability. J. Mag. Res. Imag. 30, 1249–1258. 10.1002/jmri.21967 19938037

[B18] Jin G. Q. Sun D. Xia X. Jiang Z. F. Cheng B. Ning Y. (2022). Bioorthogonal lanthanide molecular probes for near-infrared fluorescence and mass spectrometry imaging. Angew. Chem. Int. Ed. 61, 202208707. 10.1002/anie.202208707 35989247

[B19] Maniaki D. Sickinger A. Barrios Moreno L. A. Aguilà D. Roubeau O. Settineri N. S. (2023). Distributive Nd-to-Yb energy transfer within pure [YbNdYb] heterometallic molecules. Inorg. Chem. 62, 3106–3115. 10.1021/acs.inorgchem.2c03940 36753476 PMC9945097

[B20] Miller K. J. Saherwala A. A. Webber B. C. Wu Y. Sherry A. D. Woods M. (2010). The population of SAP and TSAP isomers in cyclen-based lanthanide(III) chelates is substantially affected by solvent. Inorg. Chem. 49, 8662–8664. 10.1021/ic101489t 20812752 PMC3033777

[B21] Monteiro J. H. S. K. (2020). Recent advances in luminescence imaging of biological systems using lanthanide(III) luminescent complexes. Molecules 25 (9), 2089. 10.3390/molecules25092089 32365719 PMC7248892

[B22] Natrajan L. S. Villaraza A. J. L. Kenwright A. M. Faulkner S. (2009). Controlled preparation of a heterometallic lanthanide complex containing different lanthanides in symmetrical binding pockets. Chem. Commun. 40, 6020–6022. 10.1039/b913702e 19809630

[B23] Nielsen L. G. Sørensen T. J. (2019). Including and declaring structural fluctuations in the study of lanthanide(III) coordination Chemistry in solution. Inorg. Chem. 59 (1), 94–105. 10.1021/acs.inorgchem.9b01571 31687812

[B24] Nonat A. Bahamyirou S. Lecointre A. Przybilla F. Mély Y. Platas-Iglesias C. (2019). Molecular upconversion in water in heteropolynuclear supramolecular Tb/Yb assemblies. J. Am. Chem. Soc. 141, 1568–1576. 10.1021/jacs.8b10932 30612432

[B25] Pujales-Paradela R. Savić T. Pérez-Lourido P. Esteban-Gómez D. Angelovski G. Botta M. (2019). Lanthanide complexes with ^1^H paraCEST and ^19^F response for magnetic resonance imaging applications. Inorg. Chem. 58, 7571–7583. 10.1021/acs.inorgchem.9b00869 31094193

[B26] Rivas C. Stasiuk G. J. Gallo J. Minuzzi F. Rutter G. A. Long N. J. (2013). Lanthanide(III) complexes of rhodamine-DO3A conjugates as agents for dual-modal imaging. Inorg. Chem. 52, 14284–14293. 10.1021/ic402233g 24304423 PMC4024063

[B27] Sastri V. S. Bünzli J.-C. Rao V. R. Rayudu G. V. S. Perumareddi J. R. (2003). “Spectroscopy of lanthanide complexes,” in Modern aspects of rare earths and their complexes (United States: American Chemical Society), 569–731. 10.1016/B978-044451010-5/50022-5

[B28] Sørensen T. J. Faulkner S. (2018). Multimetallic lanthanide complexes: Using kinetic control to define complex multimetallic arrays. Acc. Chem. Res. 51, 2493–2501. 10.1021/acs.accounts.8b00205 30222311

[B29] Sørensen T. J. Tropiano M. Kenwright A. M. Faulkner S. (2017). Triheterometallic lanthanide complexes prepared from kinetically inert lanthanide building blocks. Eur. J. Inorg. Chem. 2017, 2165–2172. 10.1002/ejic.201700027

[B30] Tircso G. Webber B. C. Kucera B. E. Young V. G. Woods M. (2011). Analysis of the conformational behavior and stability of the SAP and TSAP isomers of lanthanide(III) NB-DOTA-type chelates. Inorg. Chem. 50, 7966–7979. 10.1021/ic2012843 21819053 PMC3204396

[B31] Tropiano M. Blackburn O. A. Tilney J. A. Hill L. R. Just Sørensen T. Faulkner S. (2015). Exploring the effect of remote substituents and solution structure on the luminescence of three lanthanide complexes. J. Lumin. 167, 296–304. 10.1016/j.jlumin.2015.06.035

[B32] Xu W. Alam Bony B. Rong Kim C. Su Baeck J. Chang Y. Eun Bae J. (2013). Mixed lanthanide oxide nanoparticles as dual imaging agent in biomedicine. Sci. Rep. 3, 3210. 10.1038/srep03210 24220641 PMC3826100

[B33] Zaïm A. Eliseeva S. V. Guénée L. Nozary H. Petoud S. Piguet C. (2014). Lanthanide-to-Lanthanide energy-transfer processes operating in discrete polynuclear complexes: Can trivalent europium Be used as a local structural probe? Chem. Eur. J. 20, 12172–12182. 10.1002/chem.201403206 25099883

