# Supplementary material for: Heterometallic lanthanide complexes with site-specific binding that enable simultaneous visible and NIR-emission
Source: Front Chem. 2023 Jul 31;11:1232690. doi: 10.3389/fchem.2023.1232690 (PMC10424921; doi:10.3389/fchem.2023.1232690)
Supplement: Supplementary file 1 [file DataSheet1.PDF]

Supplementary Material

Heterometallic lanthanide complexes with site-specific binding that enable simultaneous visible and NIR-emission

Matthew E. Thornton, Jake Hemsworth, Sam Hay, Patrick Parkinson, Stephen Faulkner, Louise S. Natrajan*

*** Correspondence:** Corresponding Author: louise.natrajan@manchester.ac.uk

Contents

1 Synthetic procedures and characterization 2
1.1 General Methods 2
1.2 1,4,7-tris(tert-butoxycarbonylmethyl)-1,4,7,10-tetraazacyclododecane (1) 2
1.3 2-chloro-N-(4-nitro-phenyl)-acetamide (2) 5
1.4 10-[1,4,7-tris(tert-butoxycarbonylmethyl)-1,4,7,10-tetraazacyclododecan-1-yl]-N-(4-nitro-phenyl)-acetamide (3) 9
1.5 10-[1,4,7-tris(tert-butoxycarbonylmethyl)-1,4,7,10-tetraazacyclododecan-1-yl]-N-(4-amino-phenyl)-acetamide (4) 12
1.6 2,2',2''-(10-(2-((4-aminophenyl)amino)-2-oxoethyl)-1,4,7,10-tetraazacyclododecane-1,4,7-triyl)triacetic acid (5) 16
1.7 Yb-(DO3A)-aminophenyl acetamide [Yb] 19
1.8 {Yb(DO3A)}$_2$-DTPA [Yb]$_2$DTPA 21
1.9 {Yb(DO3A)$_2$-{Tb(DTPA)} [Yb$_2$Tb] 24
1.10 Eu-(DO3A)-aminophenyl acetamide [Eu] 29
1.11 {Eu(DO3A)}$_2$-DTPA [Eu]$_2$DTPA 32
1.12 {Eu(DO3A)}$_2$-{Tb(DTPA)} [Eu$_2$Tb] 35
1.13 {Yb(DO3A)}$_2$-{Eu(DTPA)} [Yb$_2$Eu] 38
2 Luminescence data 42
2.1 Equations 42
2.2 [Yb$_2$Tb] spectra 43
2.3 [Eu$_2$Tb] spectra 57
2.4 [Yb$_2$Eu] spectra 66
2.5 Supporting figures 72
3 References 79

1 Synthetic procedures and characterization

1.1 General Methods

Complexation of Ln^{III} into deprotected DO3A ligands followed a general procedure analogous to literature (Natrajan et al., 2009). Complexation of Ln^{III} into deprotected DTPA ligands followed the same general procedure with $[Yb]_2$DTPA and $[Eu]_2$DTPA starting materials.

DO3A-(triacetic acid)-aminophenyl acetamide (**5**) was dissolved in methanol and stirred. $Ln(OTf)_3$ dissolved in methanol was slowly added dropwise and the reaction mixture warmed gently to 40°C for 72 h. All volatiles were removed under reduced pressure and the residue dissolved in minimal deionized water. The pH was adjusted to ~10 with 0.1 M NaOH to precipitate any uncomplexed Ln^{III} as the hydroxide, and the solution filtered through a pad of celite. The pH was then readjusted to ~6 with 0.1 M HCl and all solvents removed under reduced pressure. Minimal ethanol was added and the solution and sonicated to dissolve, then left to stand at 4°C for several hours before filtration and washed with ethanol. All volatiles were then removed and the crude product was re-dissolved in minimal methanol. The product was slowly precipitated by layering the concentrated methanol solution with diethyl ether and allowed to diffuse for 48 hours at room temperature. The solid was filtered, washed with diethyl ether and dried under vacuum to isolate the complex as a white solid.

1.2 1,4,7-tris(tert-butoxycarbonylmethyl)-1,4,7,10-tetraazacyclododecane (1)

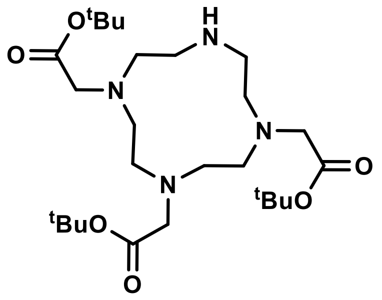

Figure S1.1: Chemical structure of compound **1**.

1,4,7,10-tetraazacyclododecane (17.4 g, 0.10 mol) and sodium hydrogen carbonate (28 g, 0.33 mol) were stirred at 0°C in acetonitrile. *Tert*-butyl bromoacetate (45 mL, 0.30 mol) dissolved in acetonitrile was added dropwise over 4 hours and the mixture was stirred at room temperature for 48 hours. The resulting inorganic salts that precipitated were filtered off and washed with acetonitrile. The filtrate was evaporated under reduced pressure (< 40°C) to leave a white solid. Warm toluene (< 50°C) was added to the crude product mixture and stirred, then left to stand at room temperature overnight. The white powder precipitate was filtered and washed with toluene then diethyl ether and dried under vacuum to isolate the product as a white powder in 44% yield (22.9 g).

^{1}H NMR (500 MHz, $CDCl_3$) δ (ppm): 1.45 (s, 9H, ^{t}Bu **H**), 1.46 (s, 18H, ^{t}Bu **H**), 2.87 (br. s, 4H N-C**H**$_2$), 2.92 (m, 8H, N-C**H**$_2$), 3.10 (m, 4H, NH-C**H**$_2$), 3.29 (s, 2H, CO-C**H**$_2$), 3.37 (s, 4H, CO-C**H**$_2$), 10.03 (br. s, 1H, N**H**). ^{13}C NMR (126 MHz, $CDCl_3$) δ (ppm): 28.33, 28.37 (^{t}Bu **C**H$_3$), 47.67, 49.33, 51.35, 51.49 (NH-**C**H$_2$), 58.37 (CO-**C**H$_2$), 81.83, 81.99 (^{t}Bu **C**), 169.76,170.65 (**C**O). EI-MS: *m/z* = 515 $[M]^+$ (100%), 537 [M+Na]+ (20 %). IR (FT-IR**)** ν (cm^{-1}): 2998, 2979, 2941, 2852 (sp^3 C-H stretch); 2732; 1718 (C=O stretch); 1450, 1368 (C-H bend); 1254 (acyl C-O stretch); 1147 (alkoxy C-O stretch). CHN elemental analysis $C_{26}H_{50}N_4O_6{\cdot}HBr{\cdot}H2O$ (%): Expected C 50.89, H 8.71, N 9.13; found C 50.94, H 8.71, N 9.45.

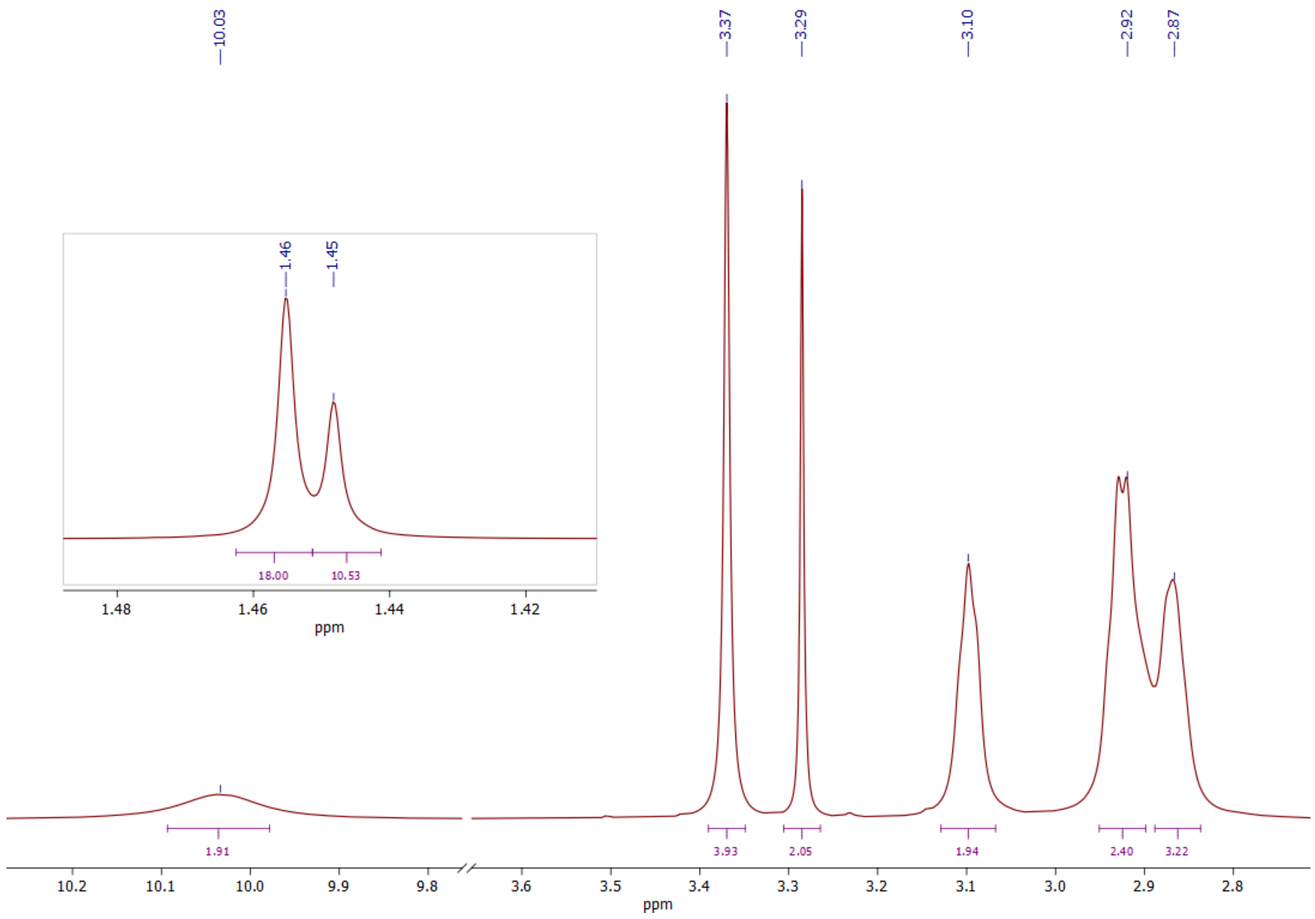

Figure S1.2: ^{1}H NMR spectrum of compound **1**. Spectra between 3.6 – 9.8 ppm omitted for clarity.

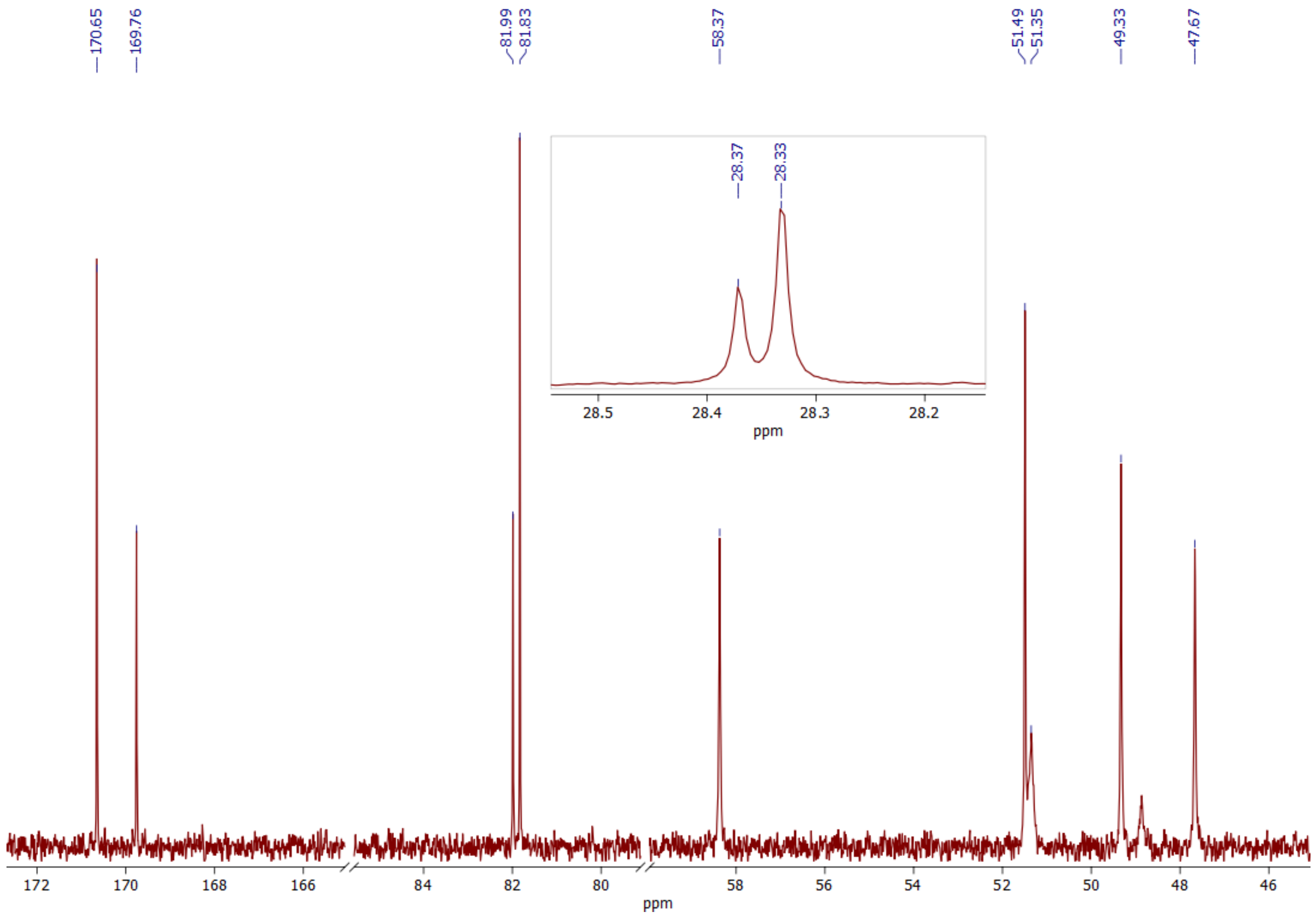

Figure S1.3: ^{13}C NMR spectrum of compound **1**. Spectra between 59 – 80 and 86 – 166 ppm omitted for clarity.

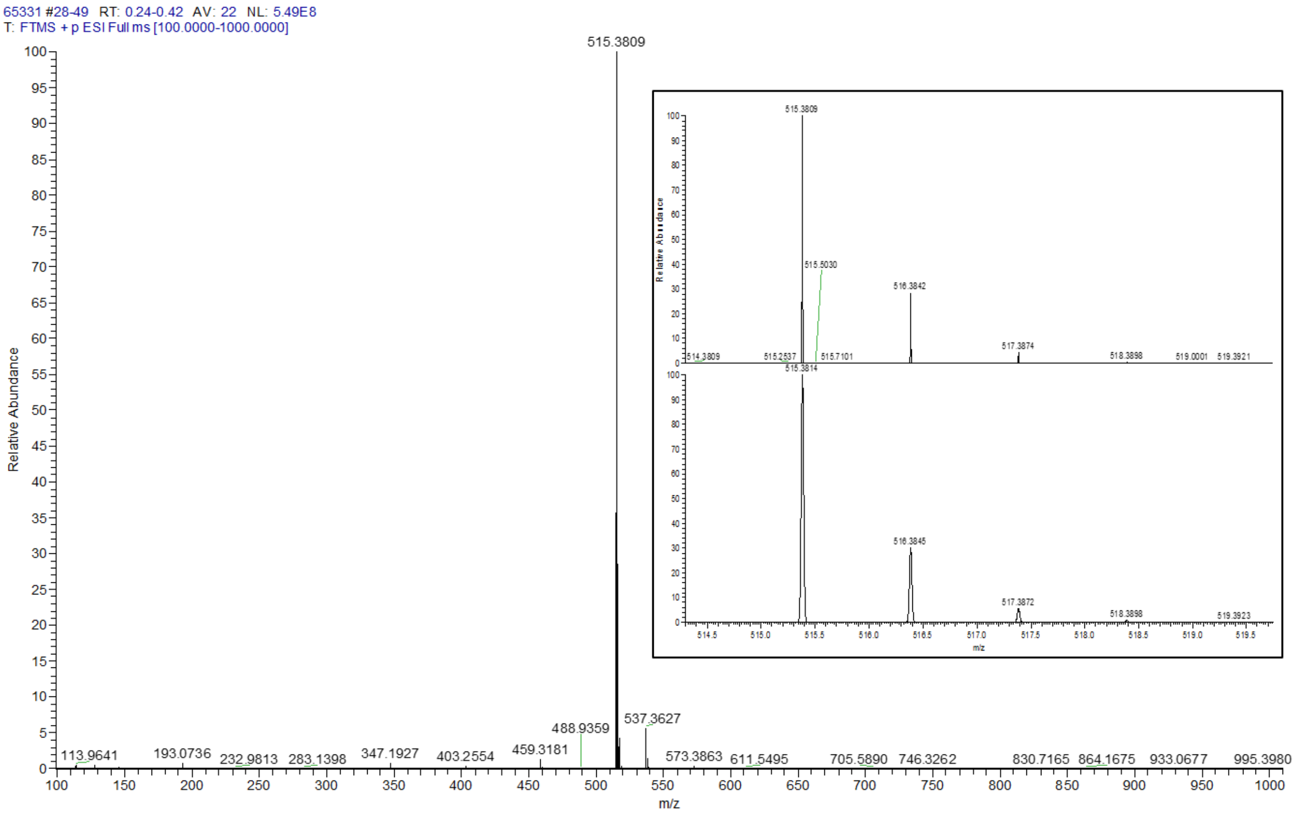

Figure S1.4: Accurate mass spectrum of compound **1**. Inset: measured (top) and calculated (bottom) splitting pattern for the $[M+H]^+$ adduct.

1.3 2-chloro-N-(4-nitro-phenyl)-acetamide (2)

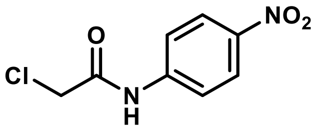

Figure S1.5: Chemical structure of compound **2**.

4-Nitroaniline (10 g, 0.072 mol) was dissolved in acetonitrile. Sodium hydrogen carbonate was added (10.1 g, 0.12 mol) and the mixture stirred at 0°C. Chloroacetyl chloride (6 mL, 0.075 mol) diluted in acetonitrile was added dropwise to the solution over 2 hours and the mixture allowed to stir overnight at room temperature. The sodium hydrogen carbonate was filtered off and solvent removed under reduced pressure. The yellow solid was dissolved in hot toluene and allowed to crystallize in the refrigerator overnight (~ 4°C). The solid was filtered and washed with cold toluene, to yield a yellow powder in 94% yield, (14.6 g).

^{1}H NMR (500 MHz, $CDCl_3$) δ: 4.24 (s, 2H, C**H**$_2$), 7.77 (dt, 2H, $^3J_{H\text{-}H}$ = 9.1 Hz, $^4J_{H\text{-}H}$ = 3 Hz, NH-Ar **H**), 8.26 (dt, 2H, $^3J_{H\text{-}H}$ = 9.1 Hz, $^4J_{H\text{-}H}$ = 3 Hz, NO_2-Ar **H**), 8.49 (br. s, 1H, N**H**). ^{13}C NMR (126 MHz, $CDCl_3$) δ 43.21 (**C**H$_2$), 119.90 (NH-Ar **C**H), 125.55 (NO_2-Ar **C**H), 142.68 (NH-Ar **C**), 144.74 (NO_2-Ar **C**), 164.55 (**C**O). EI-MS: *m/z* = 213 $[M-H]^-$ (100%). IR (FT-IR**)** ν (cm^{-1}): 3315 (N-H stretch); 3274,

3226, 3163, 3107 (sp^2 C-H stretch); 2941 (sp^3 C-H stretch); 1685 (C=O stretch); 1623, 1566 (C=C stretch); 1502 (N=O asymm. stretch); 1405 (sp^3 C-H bend); 1336 (N=O symm. stretch); 850, 748 (sp^2 C-H bend). CHN elemental analysis $C_8H_7N_2O_3Cl$ (%): Expected C 44.77, H 3.29, N 13.05; found C 44.09, H 3.19, N 13.02.

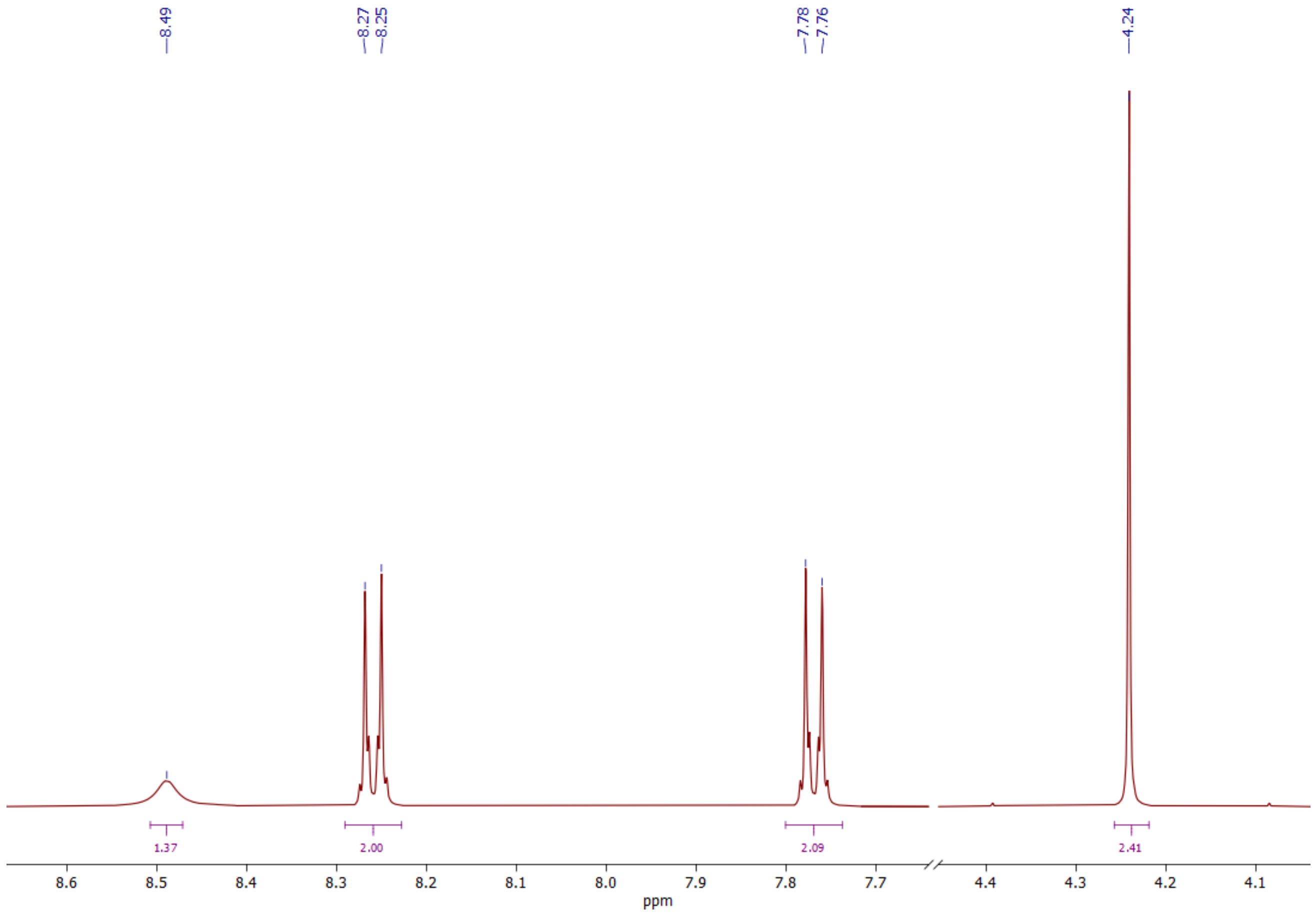

Figure S1.6: ^{1}H NMR spectrum of compound **2**. Spectra between 4.4 – 7.7 ppm omitted for clarity.

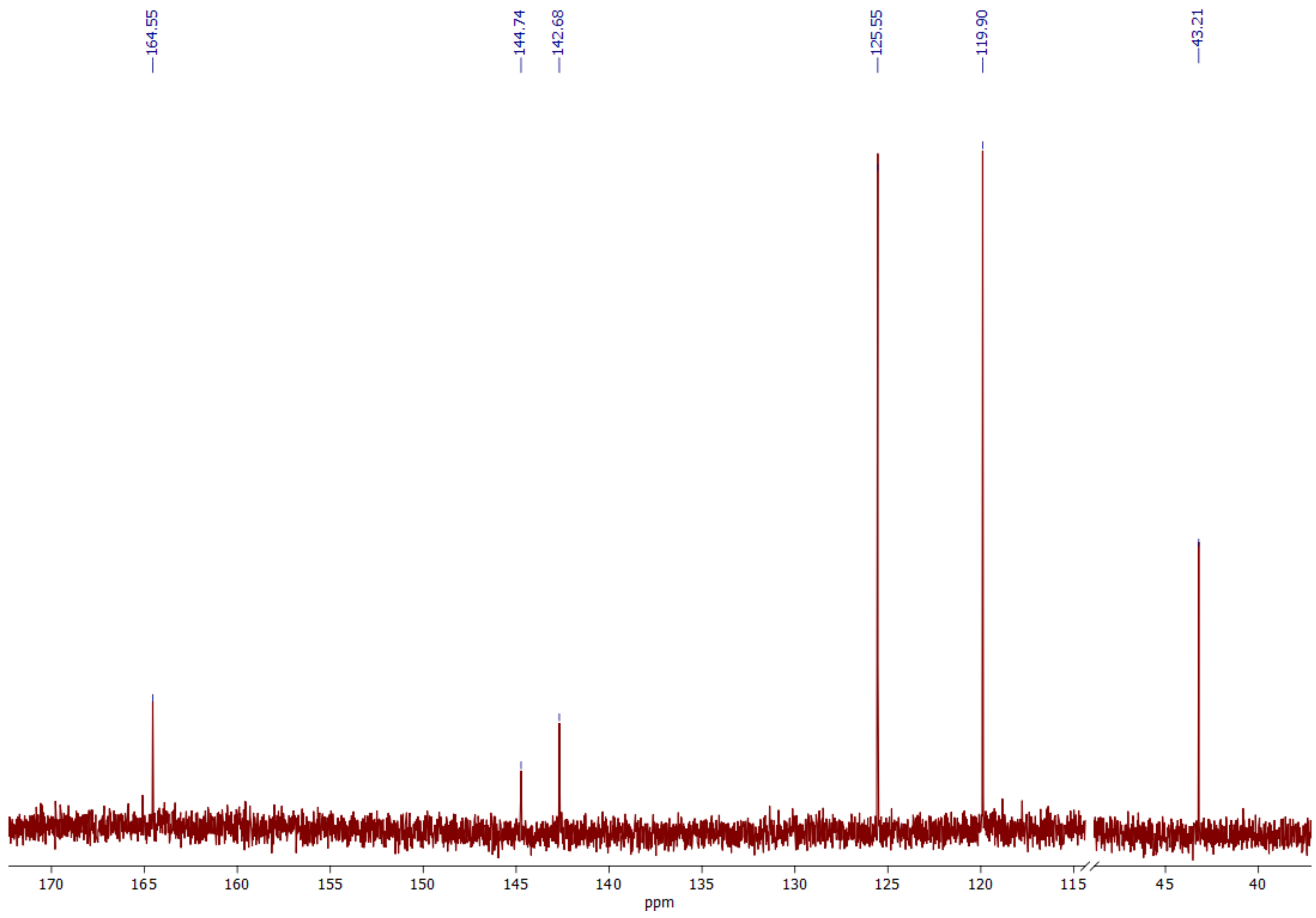

Figure S1.7: ^{13}C NMR spectrum of compound **2**. Spectra between 50 – 115 ppm omitted for clarity.

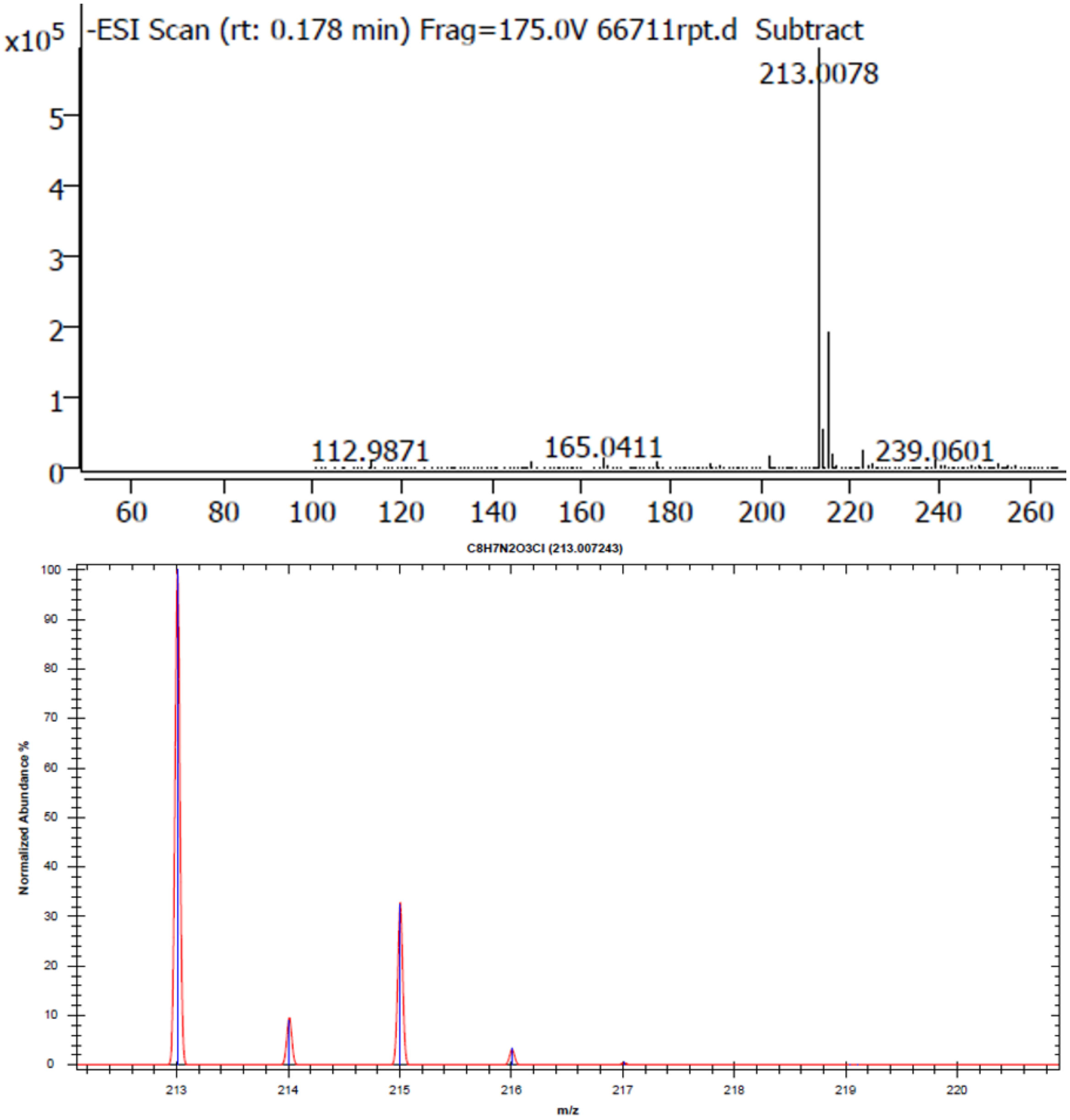

Figure S1.8: Electrospray ionization spectrum of compound **2** (top) and calculated splitting pattern for the [M-H]$^-$ adduct (bottom). Red and blue traces represent the measured and calculated spectrum, respectively.

1.4 10-[1,4,7-tris(tert-butoxycarbonylmethyl)-1,4,7,10-tetraazacyclododecan-1-yl]-N-(4-nitro-phenyl)-acetamide (3)

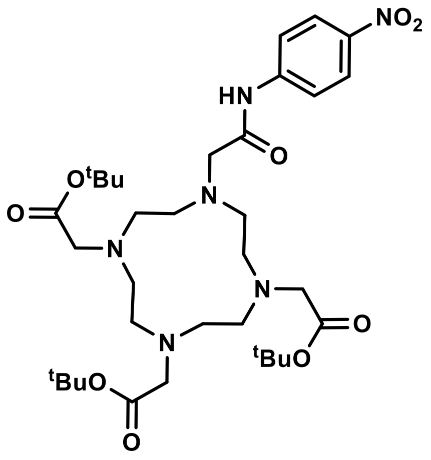

Figure S1.9: Chemical structure of compound **3**.

DO3A-(tris-*tert*-butyl ester) (**1**) (7 g, 13.6 mmol) was dissolved in acetonitrile. Sodium carbonate (3.17 g, 29.9 mmol) was added and the solution stirred at room temperature for 1 hour. 2-chloro-N-(4-nitro-phenyl)-acetamide (**2**) (3.22 g, 15 mmol) in minimal acetonitrile was added dropwise over 4 hours. The pale yellow mixture was stirred at room temperature for 24 hours then heated under reflux for 24 hours. The sodium carbonate was filtered off and the solvent removed under reduced pressure. The yellow residue was dissolved in hot toluene and left in the refrigerator overnight (~ 4°C). The crude product precipitate was filtered off and the filtrate evaporated under reduced pressure to yield another crude yellow oil product. Both residues were purified by column chromatography using a DCM:MeOH gradient. Initial treatment with pure DCM to eluted a pale yellow band, followed by two further colored fractions upon increasing the polarity with MeOH (< 5%). The pure fractions were collected and the solvent removed under reduced pressure to yield a pale yellow powder in 75% yield (7.10 g).

^{1}H NMR (500 MHz, $CDCl_3$) δ 1.48 (s, 27H, ^{t}Bu **H**), 1.90 – 3.63 (m, 16H, N-C**H**$_2$), 3.78 (s, 8H, CO-C**H**$_2$), 8.07 (dt, 2H, $^{3}J_{H\text{-}H}$ = 9.4 Hz, $^{4}J_{H\text{-}H}$ = 2.4 Hz, NH-Ar **H**), 8.12 (qt, 2H, $^{3}J_{H\text{-}H}$ = 9.4 Hz, $^{4}J_{H\text{-}H}$ = 2.4 Hz, NO_2-Ar **H**), 11.37 (br. s, 1H, N**H**). ^{13}C NMR (126 MHz, $CDCl_3$) δ 28.01, 28.04 (^{t}Bu **C**H_3), 48.48, 52.66 (N-**C**H_2), 55.70, 55.82, 57.18 (CO-**C**H_2), 82.25, 82.37 (^{t}Bu **C**), 119.79 (NH-Ar **C**H), 124.41 (NO_2-Ar **C**H), 142.81 (NH-Ar **C**), 145.51 (NO_2-Ar **C**), 172.46 (**C**ONH), 172.61 (**C**OO). EI-MS: *m/z* = 715 $[M+Na]^{+}$ (100%). IR (FT-IR**)** ν (cm^{-1}): 3315 (N-H stretch); 3182, 3137 (sp^2 C-H stretch); 2976, 2753 (sp^3 C-H stretch); 1725 (C=O); 1697 (C=O stretch); 1595, 1560 (C=C stretch); 1508 (N=O asymm. stretch); 1452 (sp^3 C-H bend); 1327 (N=O symm. stretch); 1227 (acyl C-O stretch); 1158 (alkoxy C-O stretch); 1105 (C-O stretch); 857, 734 (sp^2 C-H bend). CHN elemental analysis $C_{34}H_{56}N_6O_9\cdot0.75NaCl$ (%): Expected C 55.43, H 7.66, N 11.41; found C 55.75, H 7.29, N 9.05.

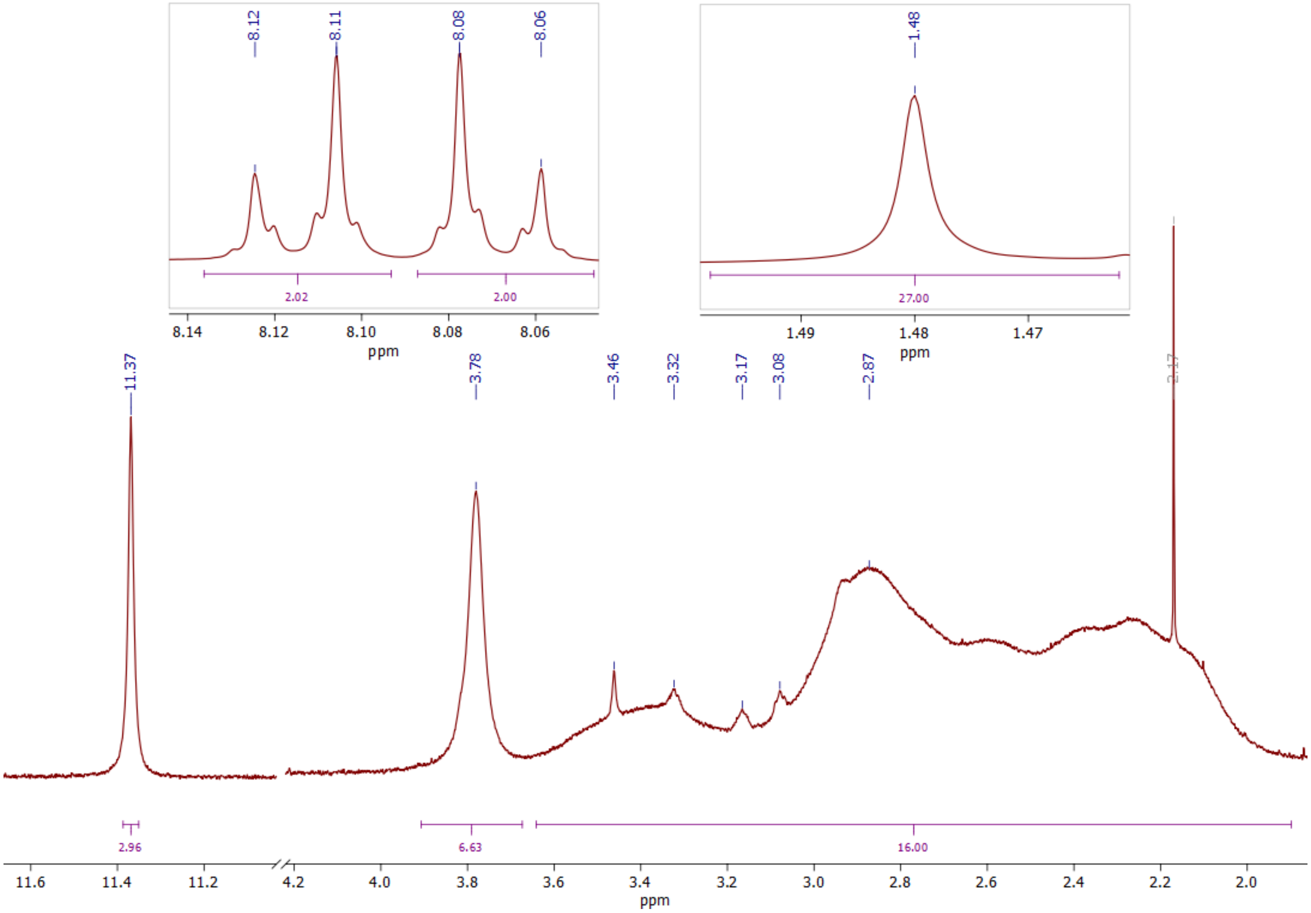

Figure S1.10: ^{1}H NMR spectrum of compound **3**. Region between 4.2 – 11.2 ppm omitted for clarity.

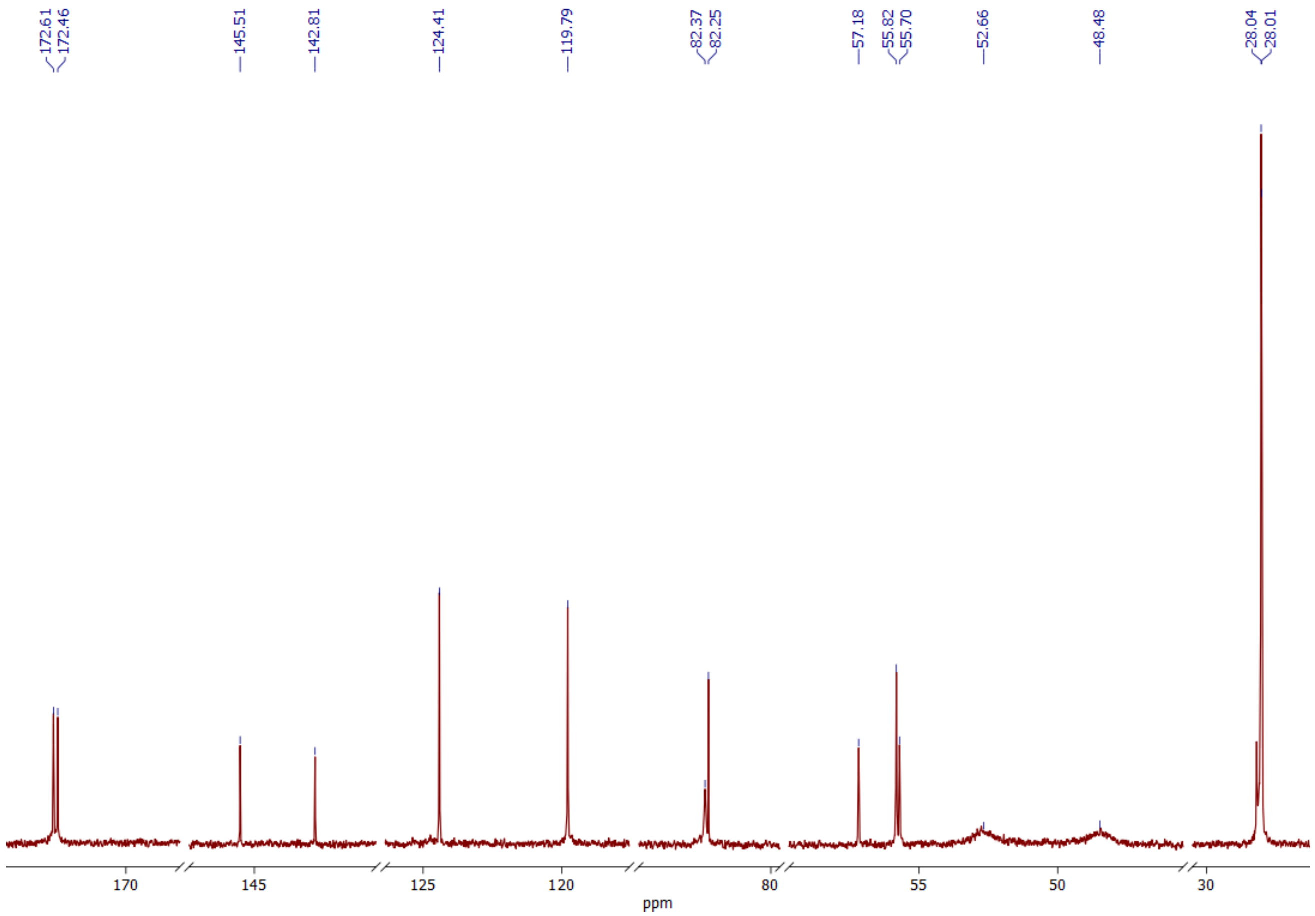

Figure S1.11: ^{13}C NMR spectrum of compound **3**. Region between 30 – 40, 60 – 80, 90 – 120, 125 – 130 and 150 - 170 ppm omitted for clarity.

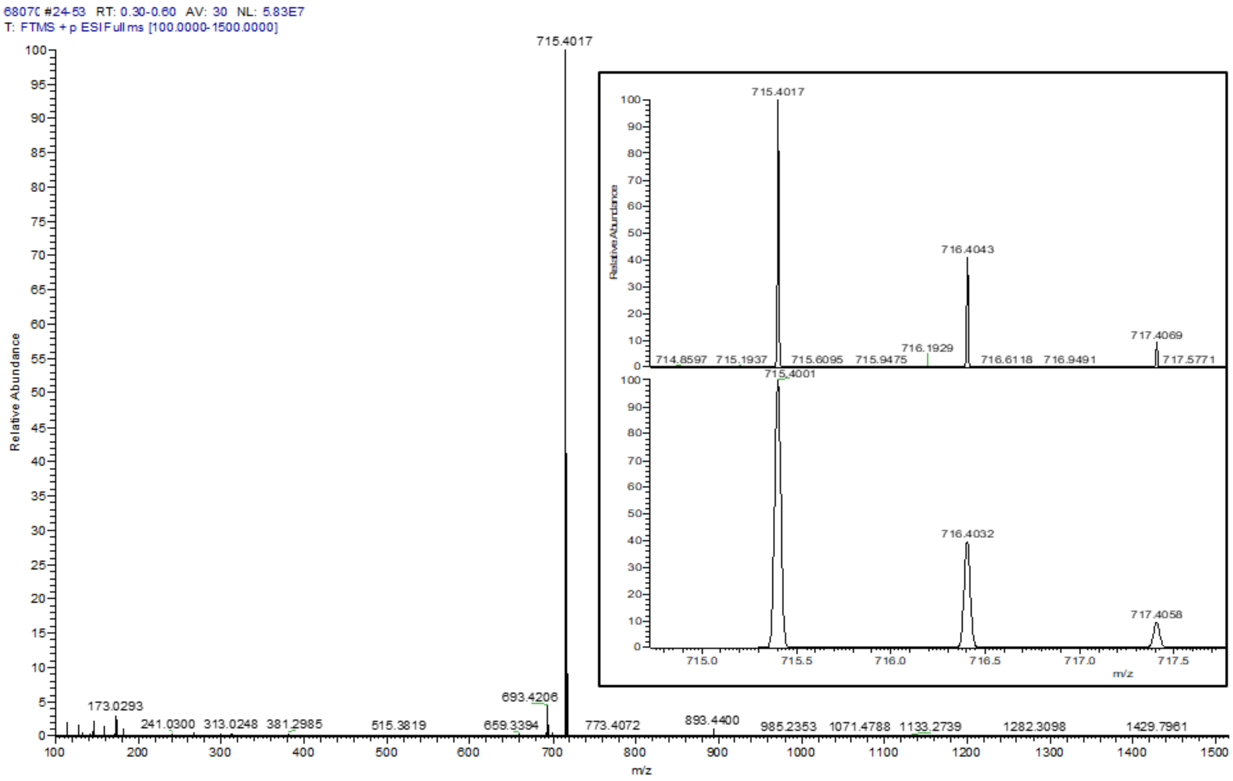

Figure S1.12: Accurate mass spectrum of compound **3**. Inset: measured (top) and calculated (bottom) splitting pattern for the $[M+Na]^+$ adduct.

1.5 10-[1,4,7-tris(tert-butoxycarbonylmethyl)-1,4,7,10-tetraazacyclododecan-1-yl]-N-(4-amino-phenyl)-acetamide (4)

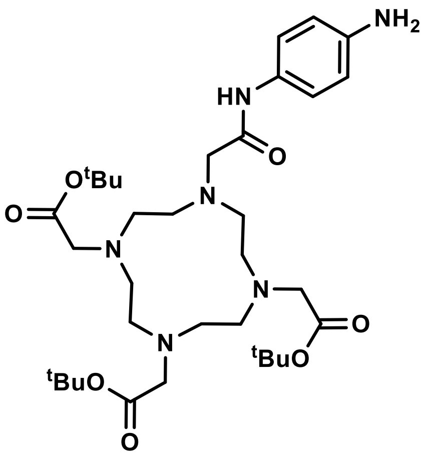

Figure S1.13: Chemical structure of compound **4**.

DO3A-(tris-*tert*-butyl ester)-nitrophenyl acetamide (**3**) (3.53 g, 5.09 mmol) was dissolved in ethanol and warmed to ~78°C. 50-60% hydrazine hydrate solution (14.1 mL) and Pd/C catalyst (wetted with ethanol) were added to the mixture, and the reaction stirred under reflux overnight. The dark solution was filtered into celite and the filtrate evaporated under reduced pressure. The resulting yellow liquid was dissolved in chloroform and extracted with distilled water to remove the hydrazine. The organic portion was dried over anhydrous $MgSO_4$ and filtered. The filtrate was evaporated under reduced pressure to yield a crude yellow oil, which was purified *via* recrystallization. The crude product was dissolved in minimal DCM and hexane added dropwise until the solution was cloudy. The mixture was warmed to dissolve the product then placed in the freezer (-18°C) overnight to crystallise and yield an off-white precipitate. The solution was filtered and the precipitate dried under reduced pressure to yield a beige powder in 48% yield (1.64 g).

^{1}H NMR (500 MHz, $CDCl_3$) δ 1.41 (br.s, 18H, ^{t}Bu **H**), 1.45 (s, 9H, ^{t}Bu **H**), 1.90 – 3.63 (m, N-C**H**$_2$) 2.62 (br. s, 4H, N-C**H**$_2$), 2.77 (br. s, 4H, N-C**H**$_2$), 2.89 (dd, $^{3}J_{H-H}$ = 14.4 Hz, $^{4}J_{H-H}$ = 5.9 Hz, 4H, N-C**H**$_2$), 2.91 (dd, $^{3}J_{H-H}$ = 14.4 Hz, $^{4}J_{H-H}$ = 5.7 Hz 4H, N-C**H**$_2$), 3.15 (s, 4H, CO-C**H**$_2$), 3.29 (s, 2H, CO-C**H**$_2$), 3.50 (br. s, 2H, N**H**$_2$), 3.63 (s, 2H, CONH-C**H**$_2$), 6.53 (dt, 2H, $^{3}J_{H-H}$ = 8.7 Hz $^{4}J_{H-H}$ = 5.1 Hz, NH_2-Ar **H**), 7.68 (dt, 2H, $^{3}J_{H-H}$ = 8.8 Hz, $^{4}J_{H-H}$ = 5.1 Hz NH-Ar **H**), 10.19 (br. s, 1H, N**H**). ^{13}C NMR (126 MHz, $CDCl_3$) δ 28.05, 28.10 (^{t}Bu **C**H_3), 51.91, 52.21 (CO-**C**H_2), 52.65 (CONH-**C**H_2), 54.85, 55.80, 55.86, 56.83, 56.91, 59.37 (N-**C**H_2), 81.08, 81.97, 82.12 (^{t}Bu **C**), 115.08 (NH_2-Ar **C**H), 121.78 (NH-Ar **C**H), 131.28 (NH-Ar **C**), 142.10 (NH_2-Ar **C**), 170.19 (**C**ONH), 170.78, 172.37 (**C**OO). EI-MS: *m/z* = 685 $[M+Na]^{+}$ (100%). IR (FT-IR**)** ν (cm^{-1}): 3182, 3122 (sp^2 C-H stretch); 2976, 2821 (sp^3 C-H stretch); 1725 (C=O stretch); 1664 (C=O stretch); 1546, 1514 (C=C stretch); 1452, 1367 (sp^3 C-H bend); 1224 (acyl C-O stretch); 1156 (alkoxy C-O stretch); 1103 (C-O stretch); 838, 755 (sp^2 C-H bend). CHN elemental analysis $C_{34}H_{58}N_6O_7{\cdot}NaCl{\cdot}2H_2O{\cdot}0.5CH_2Cl_2$ (%): Expected C 51.81, H 7.94, N 10.51; found C 51.85, H 7.75, N 10.94.

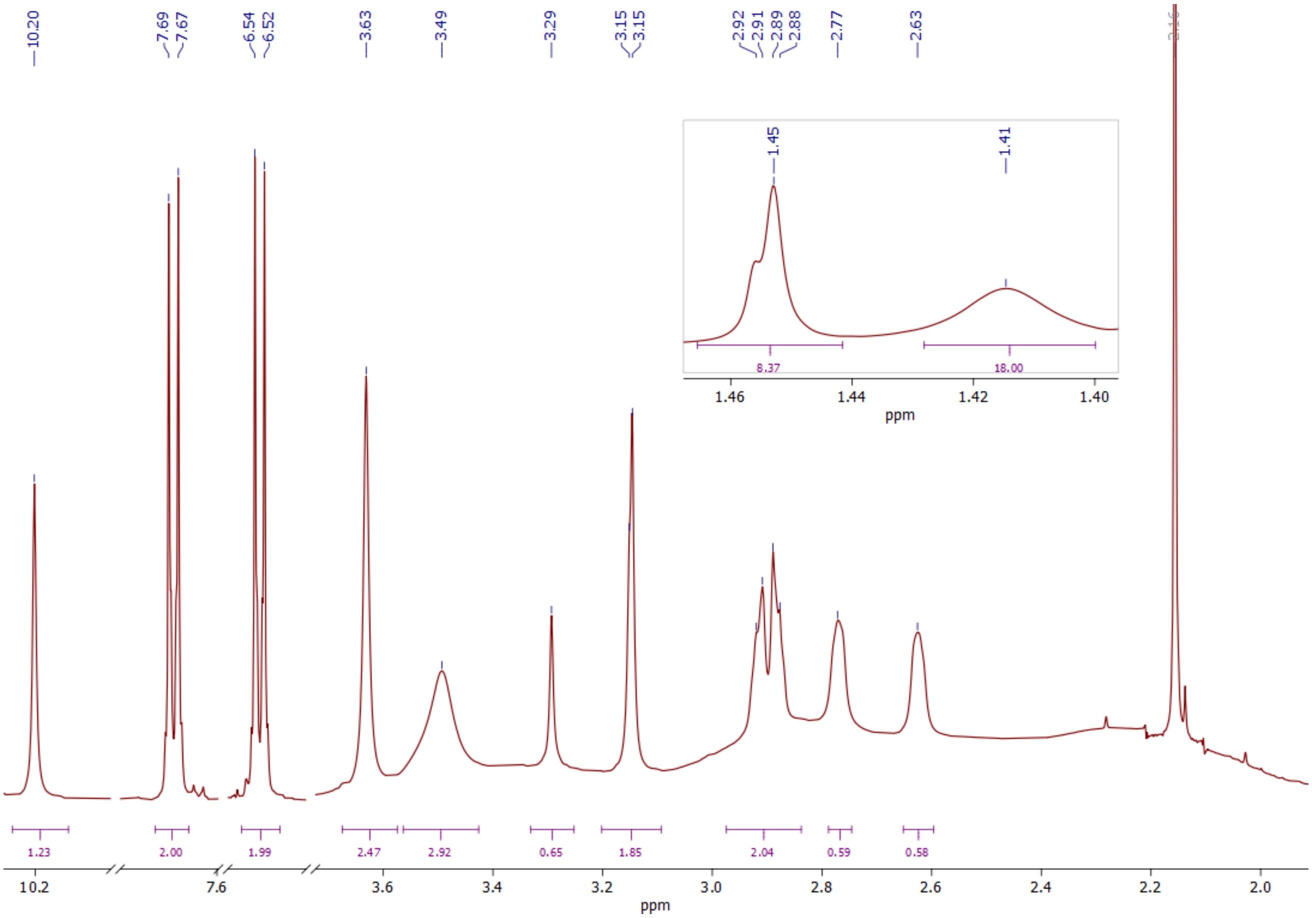
10.20
7.69
7.67
6.54
6.52
3.63
3.49
3.29
3.15
3.15
2.92
2.91
2.89
2.88
2.77
2.63
1.45
1.41
8.37
18.00
1.46
1.44
1.42
1.40
ppm
1.23
2.00
1.99
2.47
2.92
0.65
1.85
2.04
0.59
0.58
10.2
7.6
3.6
3.4
3.2
3.0
2.8
2.6
2.4
2.2
2.0
ppm

Figure S1.14: ^{1}H NMR spectrum of compound **4**. Spectra between 3.8 – 6.4, 6.6 – 7.6 and 7.8 – 10.1 ppm omitted for clarity.

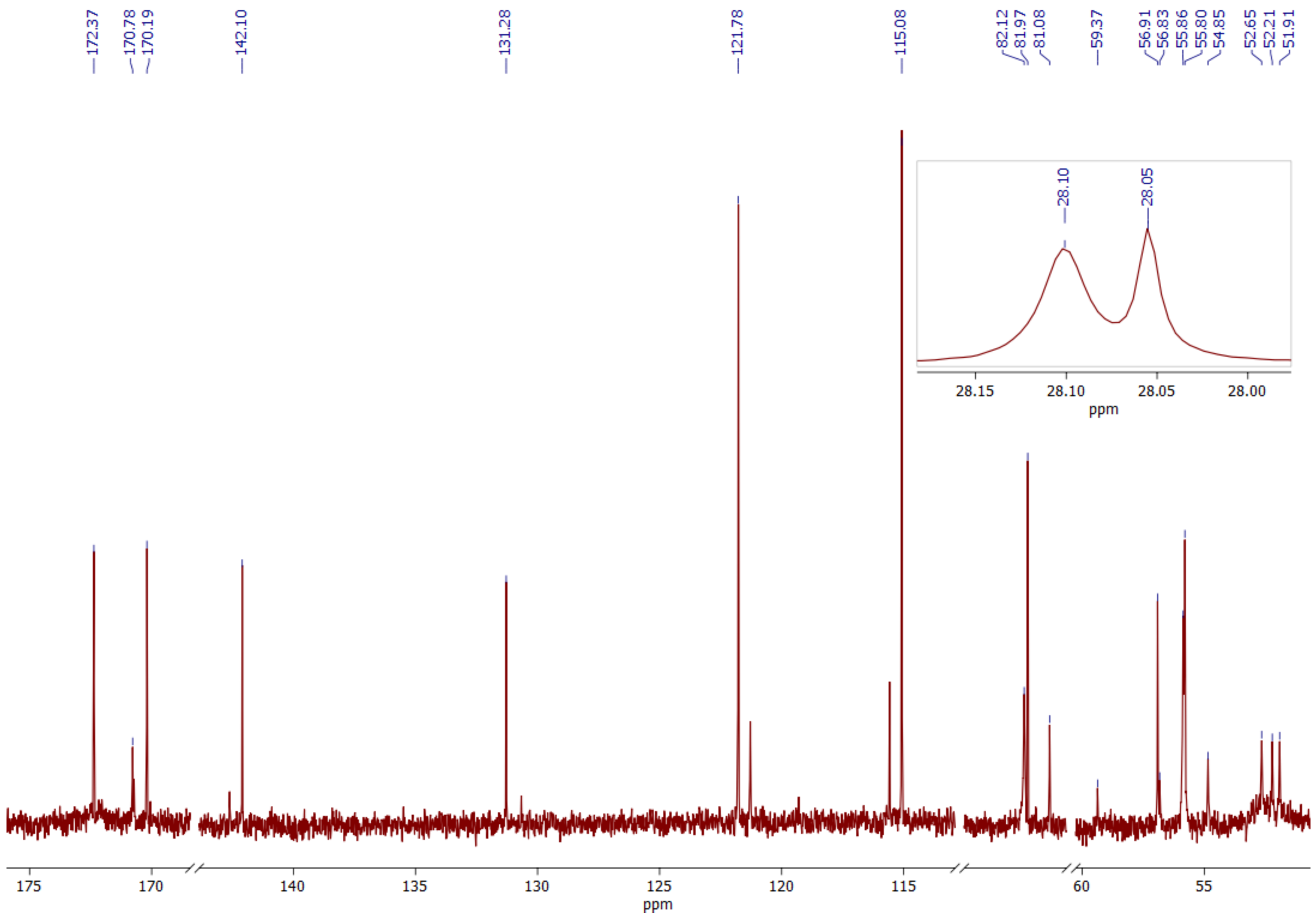

Figure S1.15: ^{13}C NMR spectrum of compound **4**. Spectra between 60 – 80, 83 – 113 and 145 - 168 ppm omitted for clarity.

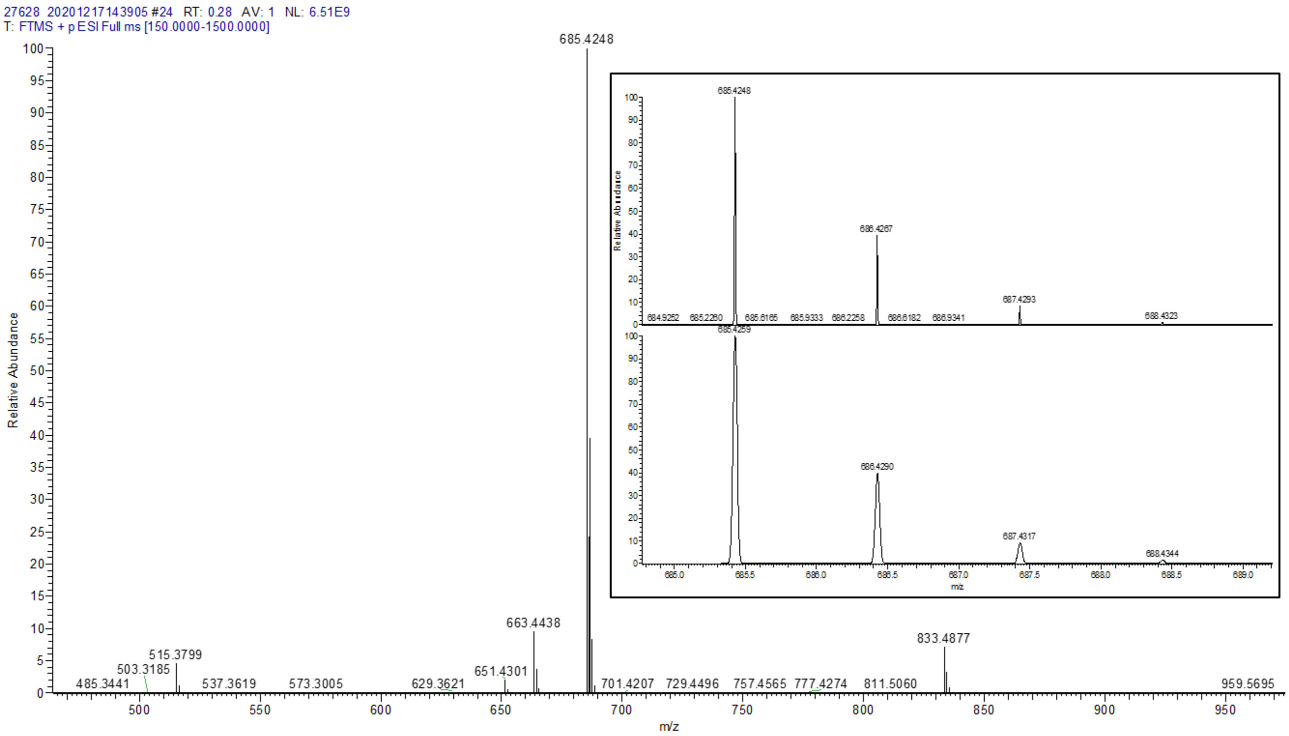

Figure S1.16: Accurate mass spectrum of compound **4**. Inset: measured (top) and calculated (bottom) splitting pattern for the $[M+Na]^+$ adduct.

1.6 2,2',2''-(10-(2-((4-aminophenyl)amino)-2-oxoethyl)-1,4,7,10-tetraazacyclododecane-1,4,7-triyl)triacetic acid (5)

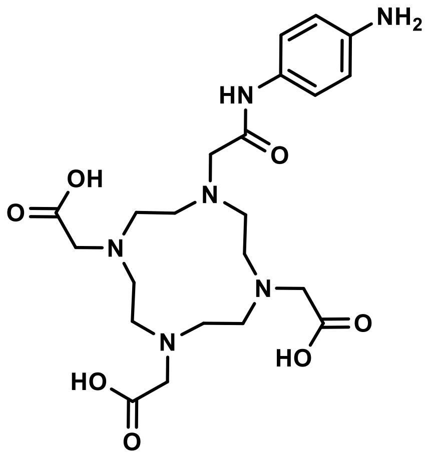

Figure S1.17: Chemical structure of compound **5**.

DO3A-(tris-*tert*-butyl ester)-aminophenyl acetamide (**4**) (1.57 g, 2.37 mmol) was dissolved in dichloromethane and trifluoroacetic acid (1:1 ratio) was added dropwise to the stirring solution. The brown mixture was allowed to stir at room temperature for 24 hours. All volatiles were then removed

under reduced pressure and the residue washed repeatedly with dichloromethane and methanol. The product was slowly precipitated by layering the concentrated methanol solution with diethyl ether and allowed to diffuse for 48 hours at room temperature. The hygroscopic product was filtered, washed with diethyl ether and dried under vacuum to afford a white solid in 74% yield (0.87 g).

^{1}H NMR (500 MHz, D_2O) δ 3.13 (m, 12H, N-C**H**$_2$), 3.46 (s, 2H, CONH-C**H**$_2$), 3.51 (m, 4H, N-C**H**$_2$), 3.65 (s, 2H, CO-C**H**$_2$), 3.83 (s, 2H, CO-C**H**$_2$), 3.84 (s, 2H, CO-C**H**$_2$), 7.17 (d, 2H, $^{3}J_{H\text{-}H}$ = 8.8 Hz, NH_2-Ar **H**), 7.49 (d, 2H, $^{3}J_{H\text{-}H}$ = 8.7 Hz, NH-Ar **H**). ^{13}C NMR (126 MHz, D_2O) δ 48.09, 48.25, 50.92, 51.63 (N-**C**H$_2$), 55.00, 55.91, 56.40 (CO-**C**H$_2$), 115.15 (NH_2-Ar **C**), 117.47 (NH-Ar **C**), 120.95 (NH_2-Ar **C**H), 122.76 (NH-Ar **C**H), 169.80, 170.63 (**C**OO), 176.92 (**C**ONH). MALDI-TOF MS (alpha/MeOH): *m/z* = 517 [M+Na]+ (100%), 533 $[M+K]^+$ (97 %), 495 $[M+H]^+$ (95 %). IR (FT-IR**)** ν (cm^{-1}): 3095 (sp^2 C-H stretch); 2961, 2847 (sp^3 C-H stretch); 1676 (C=O stretch); 1513 (C=C stretch); 1458, 1425 (O-H bend); 1198, 1126 (C-O stretch); 830, 799 720 (sp^2 C-H bend). CHN elemental analysis $C_{22}H_{34}N_6O_7$·2TFA·3H_2O (%): Expected C 40.21, H 5.45, N 10.82; found C 39.95, H 4.90, N 11.03.

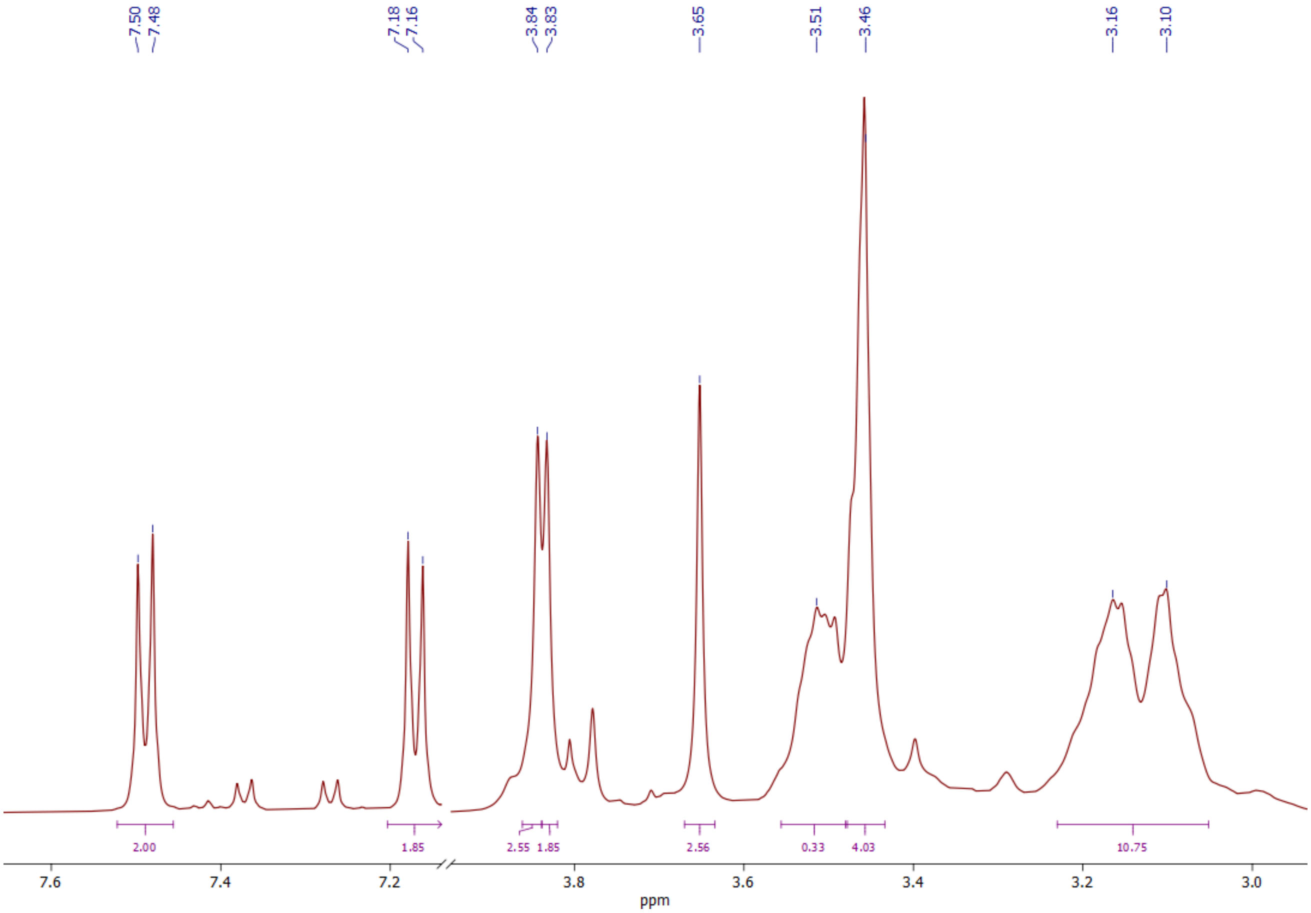

Figure S1.18: ^{1}H NMR spectrum of compound **5**. Region between 4 – 7 ppm omitted for clarity.

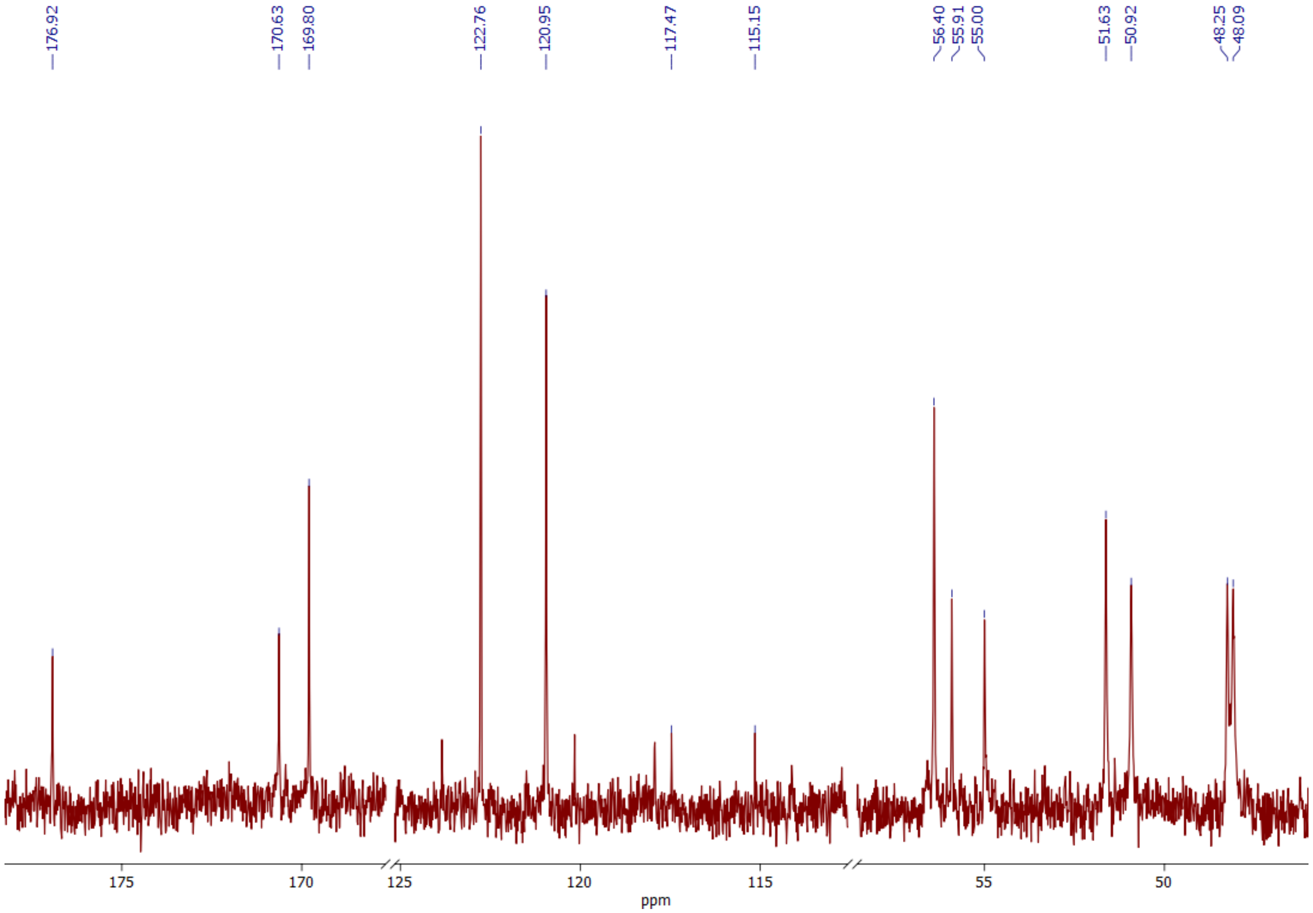

Figure S1.19: ^{13}C NMR spectrum of compound **5**. Region between 60 – 110, 125 – 165 ppm omitted for clarity.

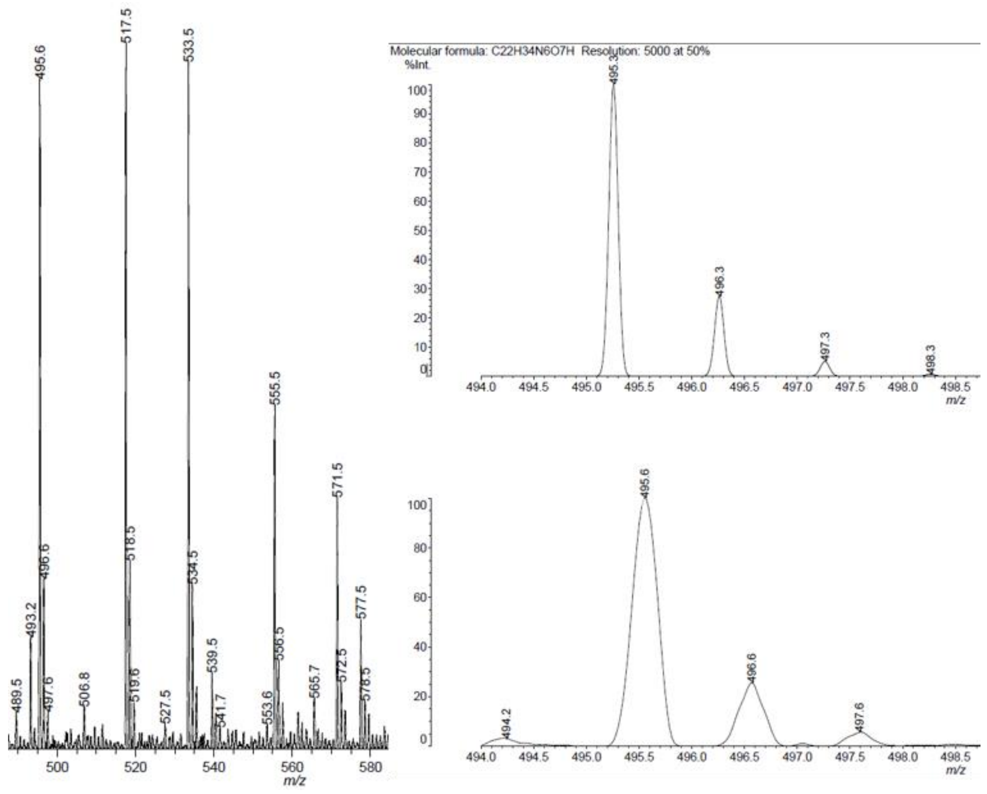

Figure S1.20: Left: MALDI spectrum of compound **5**. Right: calculated (top) and measured (bottom) splitting pattern for the $[M+H]^+$ adduct.

1.7 Yb-(DO3A)-aminophenyl acetamide [Yb]

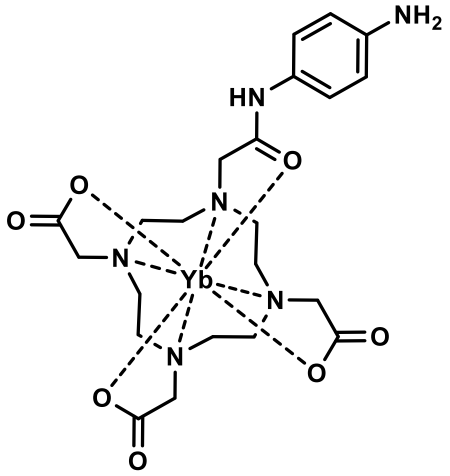

Figure S1.21: Chemical structure of compound **[Yb]**.

Compound **5** (35.4 mg, 0.072 mmol) was reacted with $Yb(OTf)_3$ (49.6 mg, 0.082 mmol) following the general complexation procedure outlined in **1.1** to result in an 83% yield (39 mg).

^{1}H NMR (500 MHz, D_2O) δ -75.18, -74.34, -71.31, -56.82, -54.53, -43.02, -40.07, -28.76, -26.27, -24.39, -20.47, 0.33, 2.22, 8.67, 9.10, 14.58, 15.11, 15.96, 18.11, 19.42, 24.01, 25.80, 31.55, 117.18, 122.05, 128.38. MALDI-TOF MS (alpha/MeOH): *m/z* = 688 $[M+Na]^+$ (100%), 710 [M+2Na-H] (51%), 704 $[M+K]^+$ (35%), 1373 $[2M+K]^+$ (8%), 1351 $[2M+Na]^+$ (4%). IR (FT-IR) ν (cm^{-1}): 3349 (N-H stretch); 2971, 2914, 2867 (sp^3 C-H stretch); 1608 (C=O stretch); 1516 (C=C stretch); 1384 (C-H bend); 1247, 1160 (C-O stretch); 1082, 1029 (C-N stretch); 834, 721 637 (sp^2 C-H bend).

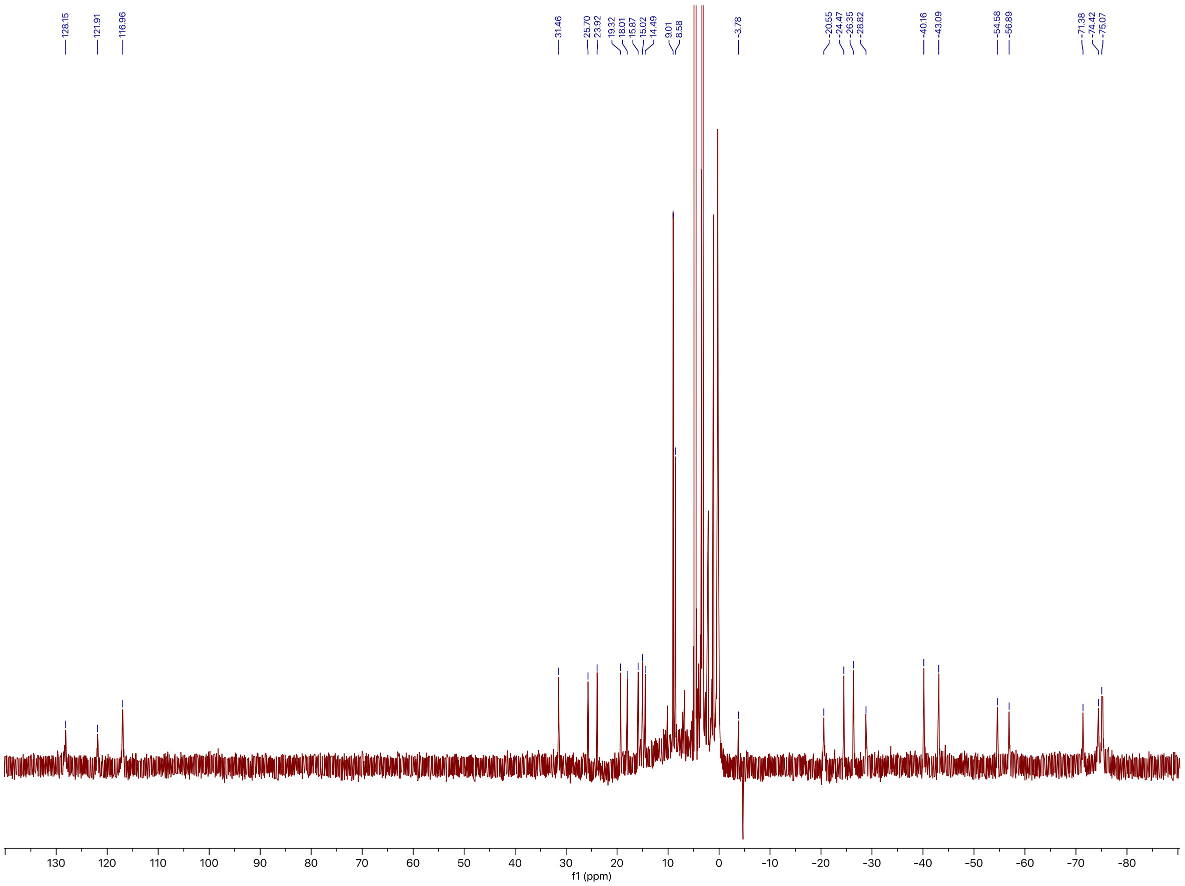

Figure S1.22: Full ^{1}H NMR spectrum of compound **[Yb]**.

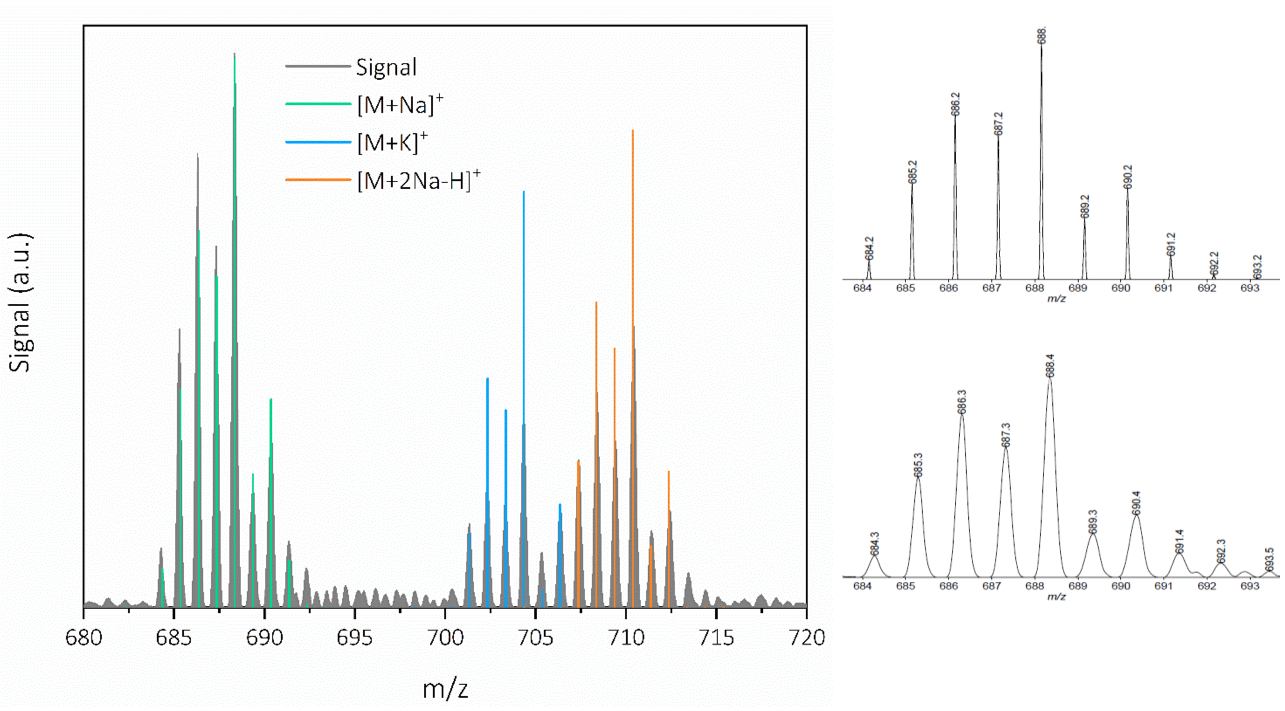

Figure S1.23: Left: MALDI spectrum of compound **[Yb]** showing [M+H]+ (green), [M+K]+ (blue), and [M+2Na-H]+ (orange) adducts. Right: calculated (top) and measured (bottom) splitting pattern for the [M+Na]+ adduct.

1.8 {Yb(DO3A)}$_2$-DTPA [Yb]$_2$DTPA

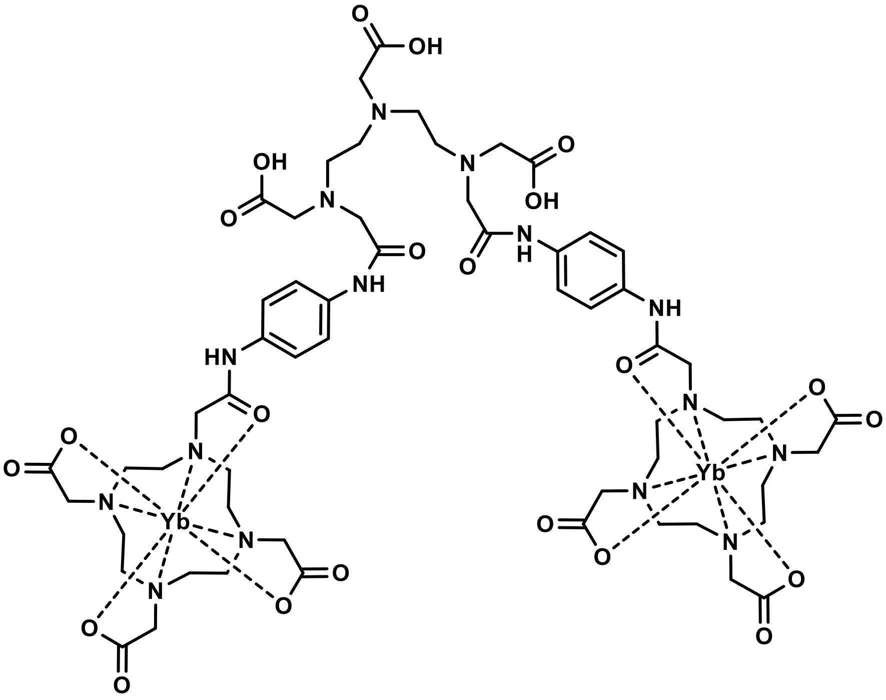

Figure S1.24: Chemical structure of compound **[Yb]$_2$DTPA**.

Under argon, complex **[Yb]** (314 mg, 0.47 mmol) was dissolved in dry dimethylformamide (DMF) and potassium carbonate (4 eq.) was added. Diethylenetriamine pentaacetic acid (DTPA) anhydride (67.5 mg, 0.19 mmol) was dissolved in anhydrous DMF and added to the stirring reaction. The reaction mixture was then heated to 55 °C for 14 days under argon. The solution was opened to air, cooled and filtered. The filtrate was reduced *in vacuo* and the residue re-dissolved in methanol and then precipitated with diethyl ether and placed in a freezer (-18 °C) overnight. The beige solid was decanted, washed with diethyl ether and dried thoroughly under vacuum. The desired complex was isolated as a hygroscopic beige solid (0.091 g, 29 %).

^{1}H NMR (500 MHz, D_2O) δ -74.32, -73.27, -72.61, -69.75, -57.49, -56.44, -43.46, -43.19, -41.09, -40.37, -26.48, -25.97, -24.93, -24.52, -21.69, -15.93, 2.84, 3.00, 3.25, 3.30, 3.34, 3.54, 3.72, 8.45, 9.04, 9.47, 10.30, 10.67, 11.05, 11.44, 11.73, 12.10, 12.95, 13.38, 13.76, 14.16, 14.79, 16.00, 16.33, 17.36, 17.73, 18.11, 19.12, 23.24, 24.19, 25.07, 26.78, 33.23, 113.88, 114.67, 115.72, 119.71, 128.43. MALDI-TOF MS (alpha/MeOH): *m/z* = 1724 $[M+K]^+$ (100%), 1762 $[M+2K-H]^+$ (44%), 1746 $[M+IPA+H]^+$ (36%), 1708 $[M+Na]^+$ (26%), 1686 $[M+H]^+$ (16%). IR (FT-IR**)** ν (cm^{-1}): 3293 (N-H stretch); 2949, 2839 (sp^3 C-H stretch); 1734 (C=O stretch); 1583 (C=C stretch); 1400, 1321 (C-H bend); 1254 (C-O stretch), 1160, 1031 (N-H bend); 840, 717, 638 (sp^2 C-H bend).

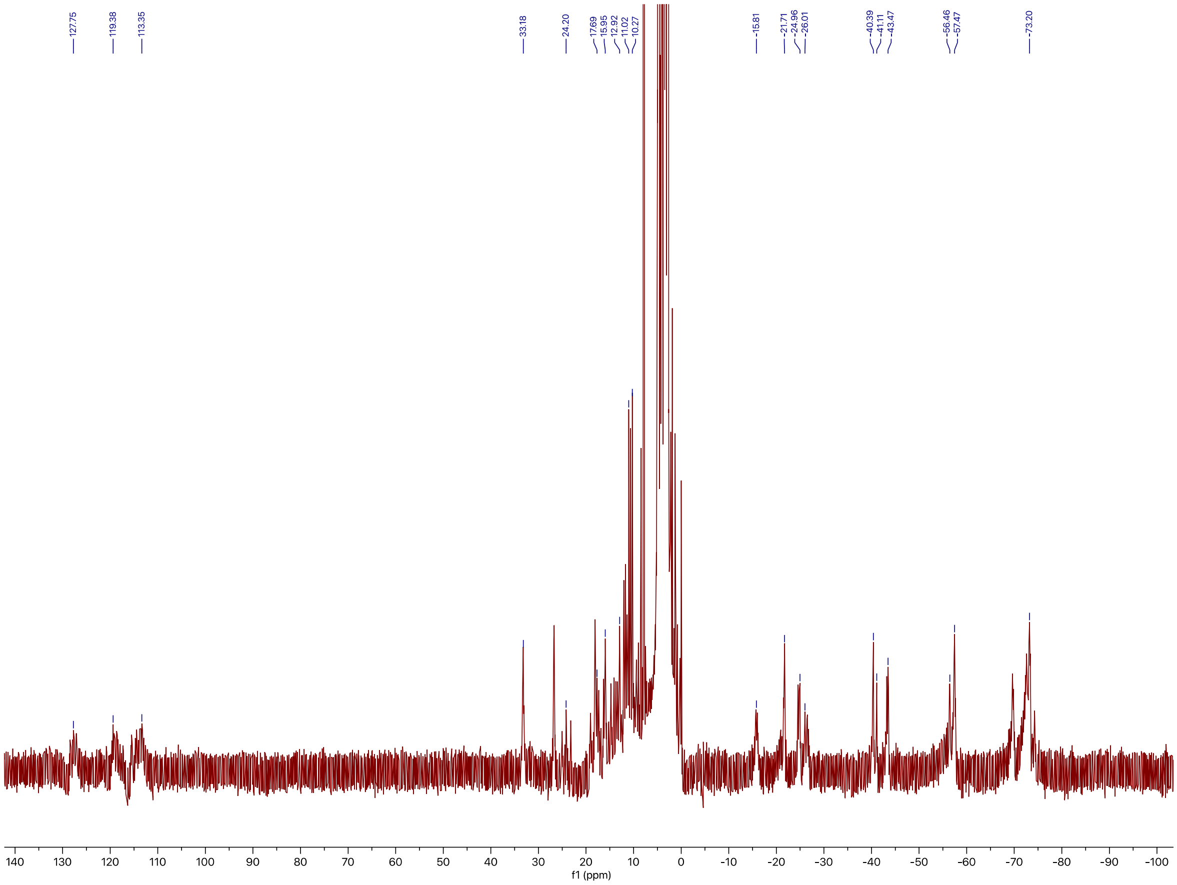

Figure S1.25: Full ^{1}H NMR spectrum of compound **$[Yb]_2$DTPA**.

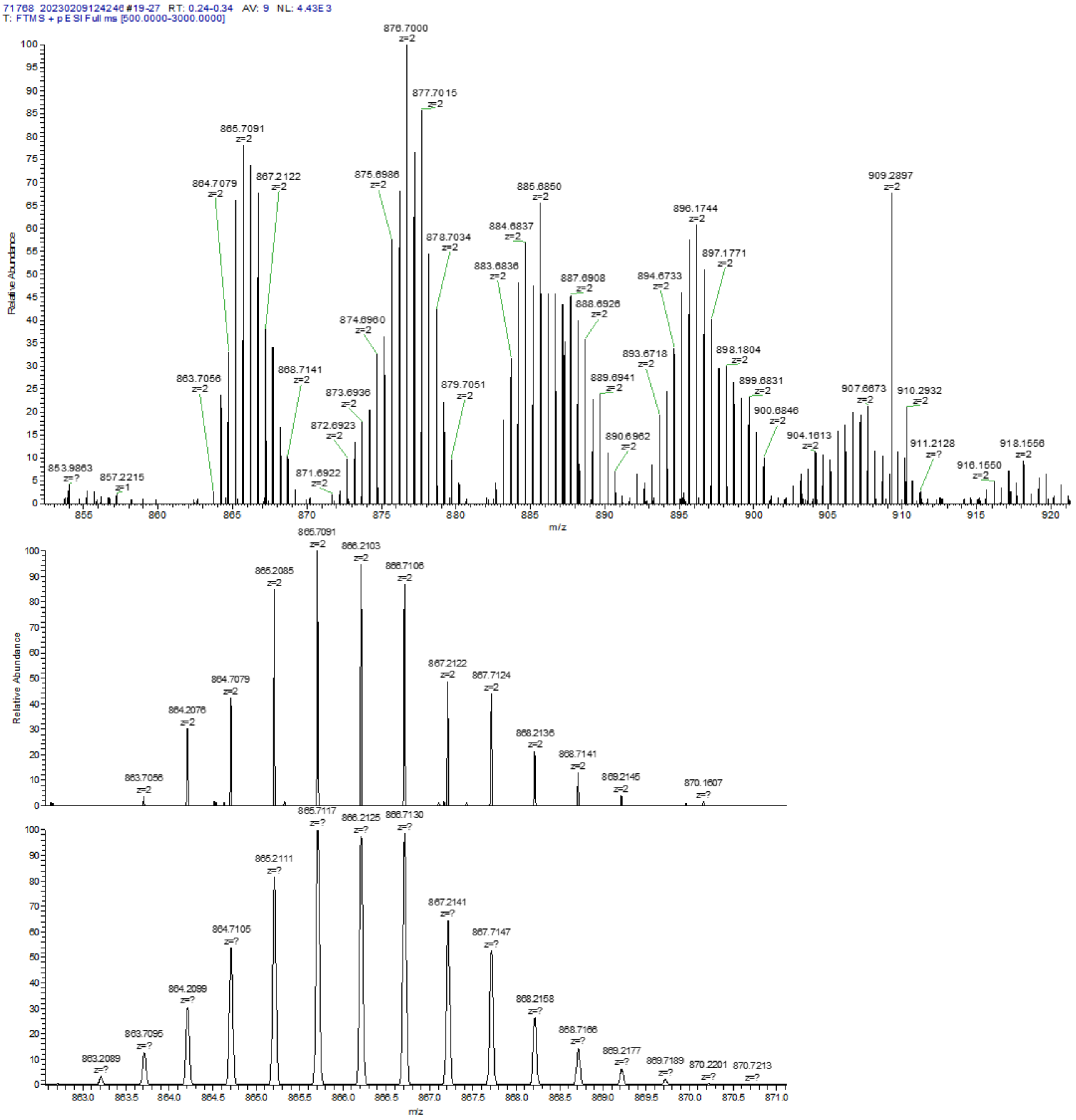

Figure S1.26: Above: Accurate mass spectrum of compound **[Yb]₂DTPA**. Below: measured (top) and calculated (bottom) splitting pattern for the $[M+2Na]^{2+}$ adduct.

1.9 {Yb(DO3A)2-{Tb(DTPA)} [Yb2Tb]

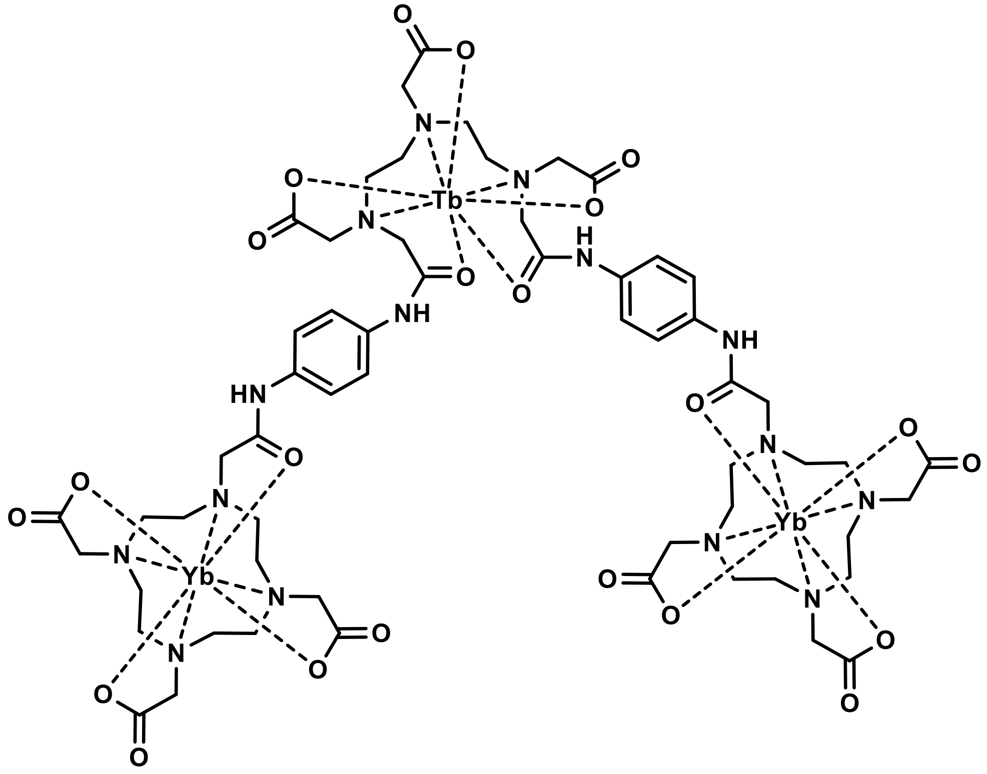

Figure S1.27: Chemical structure of compound **[Yb2Tb]**.

[Yb]2DTPA (74.2 mg, 0.044 mmol) was reacted with $Tb(OTf)_3$ (31.7 mg, 0.052 mmol) following the general complexation procedure outlined in **1.1** to result in a 53% yield (43 mg).

^{1}H NMR (500 MHz, MeOD) δ -98.41, -87.54, -86.12, -85.42, -81.11, -68.47, -64.43, -63.88, -51.79, - -48.85, -45.64, -43.33, -30.18, -28.66, -24.68, -16.70, -0.97, 0.09, 0.89, 1.16, 1.17, 1.19, 1.28, 1.38, 1.51, 10.13, 11.24, 14.85, 16.53, 18.95, 19.77, 21.23, 23.40, 25.90, 28.37, 31.16, 38.22, 125.80, 129.18, 134.34, 143.32. EI-MS: *m/z* = 1882 $[M+K]^+$ (100%), 1844 $[M+H]^+$ (78%), 1866 $[M+Na]^+$ (67%), 952 $[M+Na+K]^{2+}$, 944 $[M+2Na]^{2+}$. IR (FT-IR) ν (cm^{-1}): 3376 (N-H stretch); 2977, 2872 (sp^3 C-H stretch); 1599, 1515 (C=C stretch); 1340, 1321 (C-N stretch); 1086, 1031 (C-O stretch); 932, 844, 718, 639 (sp^2 C-H bend).

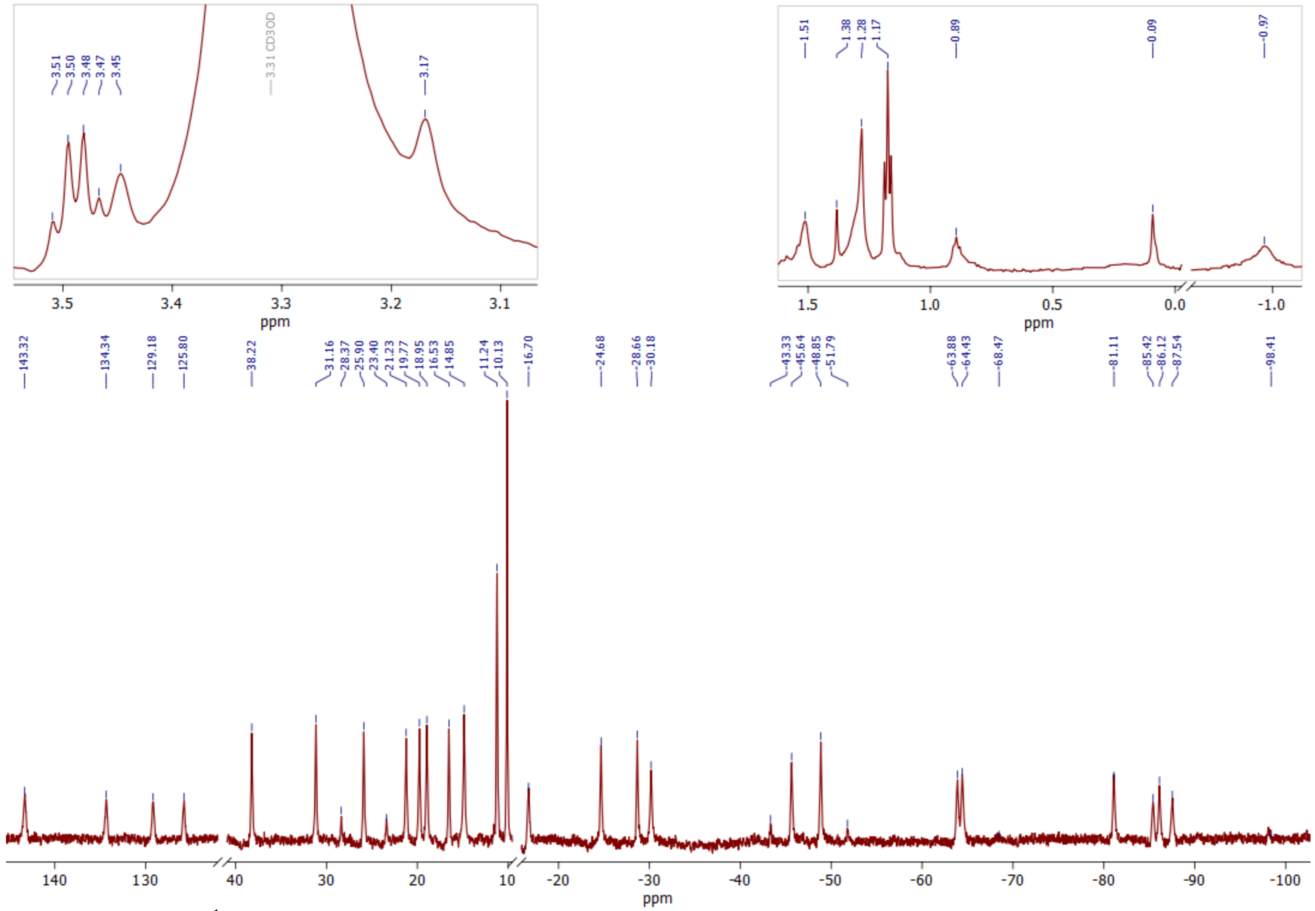

Figure S1.28a: ^{1}H NMR spectrum of compound **[Yb$_2$Tb]**. Region between -15 – 10 and 40 - 120 ppm omitted for clarity.

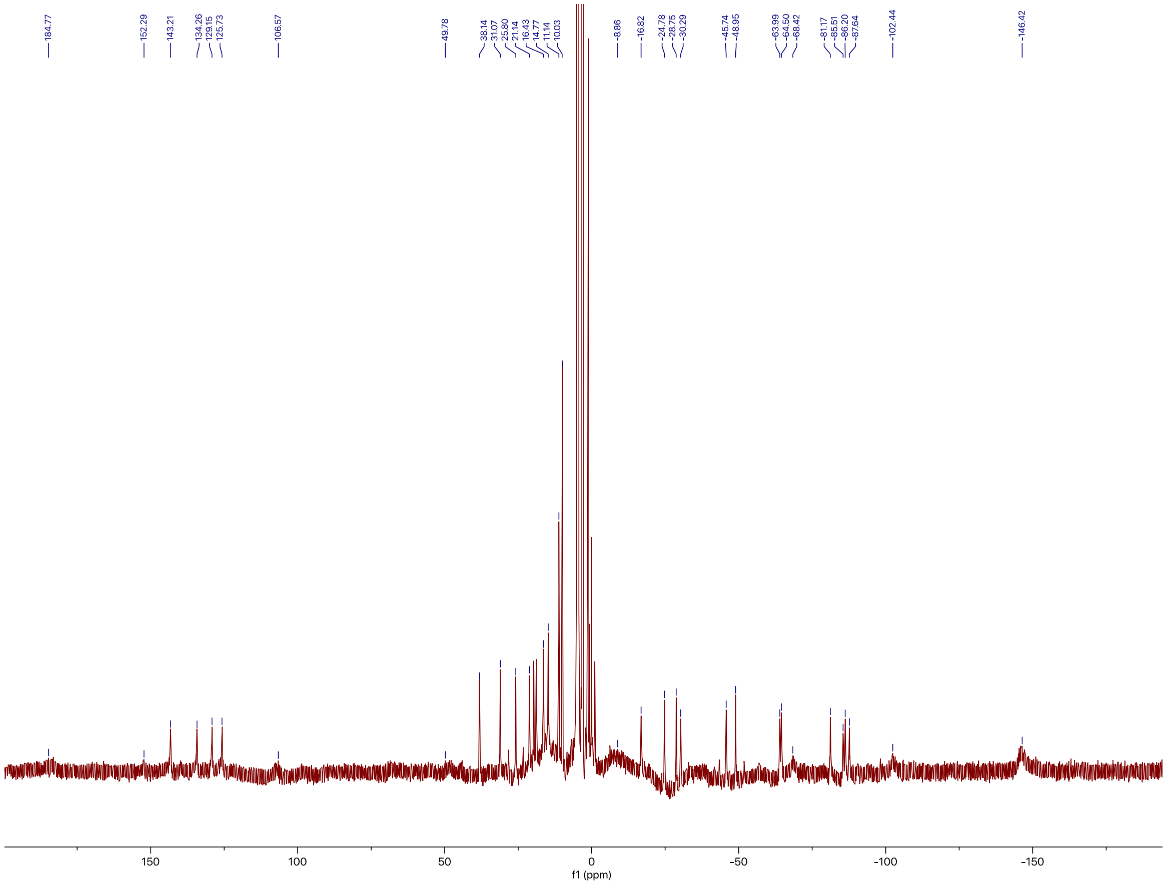

Figure S1.28b: Full ^{1}H NMR spectrum of compound **[Yb$_2$Tb]**. Note the broader resonances at higher and lower field due to [Tb(III)].

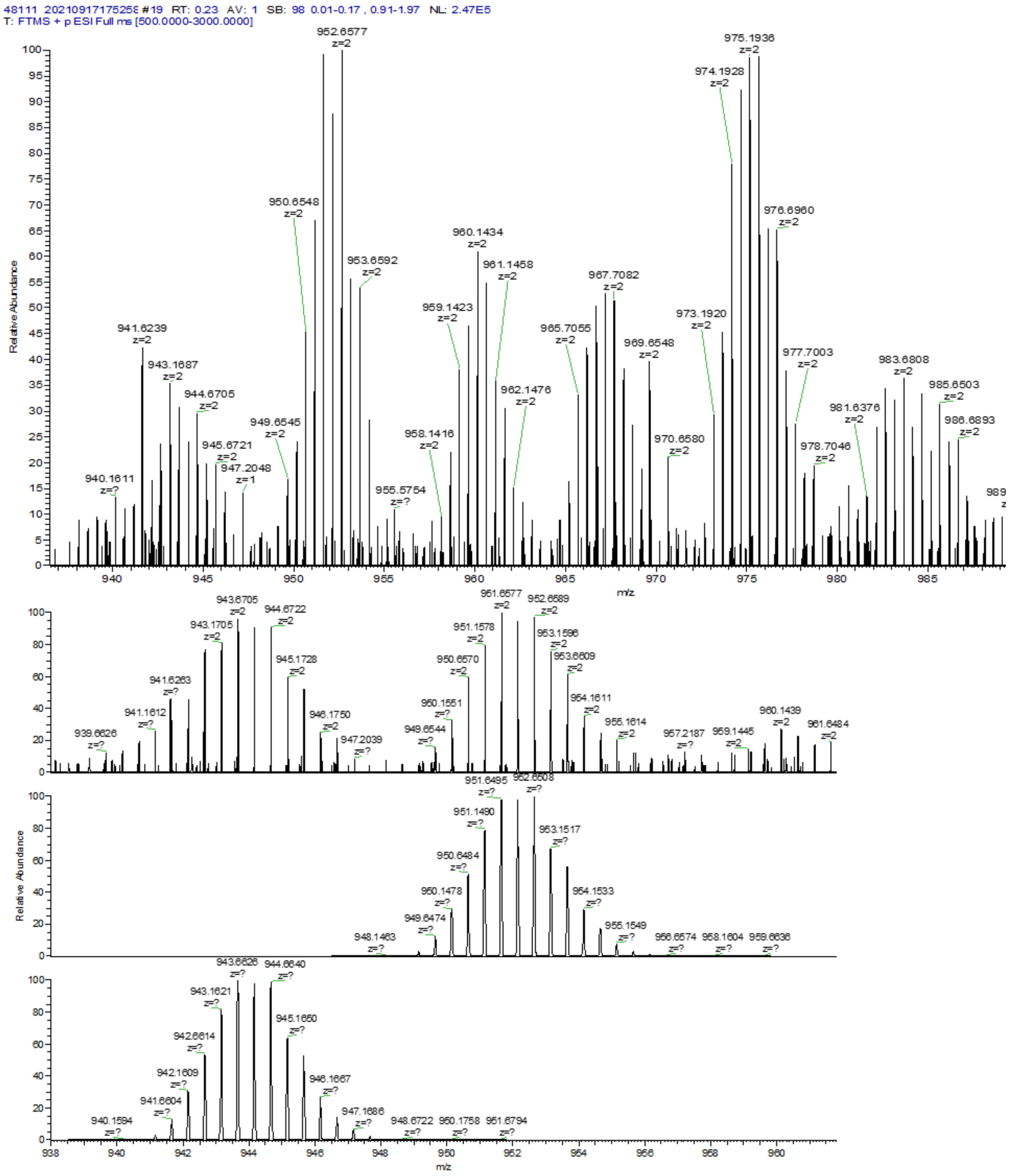

Figure S1.29: Above: Accurate mass spectrum of compound **[Yb$_2$Tb]**. Below: measured (top) and calculated splitting patterns for the [M+Na+K]$^{2+}$ (middle) and [M+2Na]$^{2+}$ (bottom) adducts.

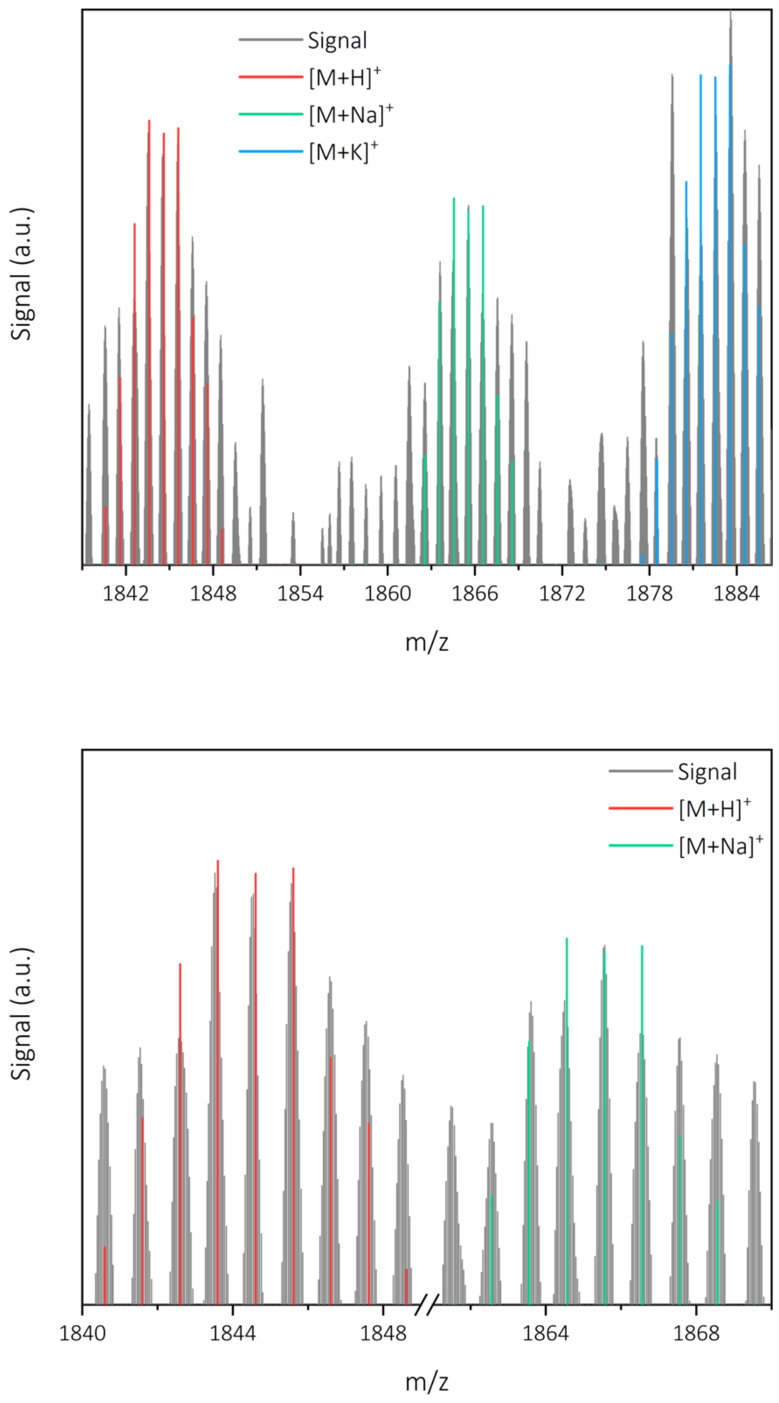

Figure S1.30: Above: Accurate mass spectrum of compound **[Yb$_2$Tb]** with calculated splitting patterns of $[M+H]^+$ (red), $[M+Na]^+$ (green) and $[M+K]^+$ (blue) adducts. Below: cropped spectra highlighting overlap between calculated and experimental data.

1.10 Eu-(DO3A)-aminophenyl acetamide [Eu]

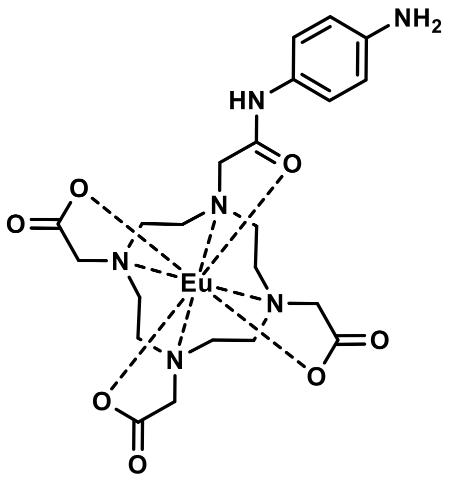

Figure S1.31: Chemical structure of compound **[Eu]**.

Compound **5** (302 mg, 0.61 mmol) was reacted with $Eu(OTf)_3$ (403 mg, 0.67 mmol) following the general complexation procedure outlined in **1.1** to result in an 77% yield (305 mg).

^{1}H NMR (500 MHz, D_2O) δ -16.58, -15.62, -14.84, -14.26, -12.44, -11.59, -11.19, -10.91, -7.80, -7.68, -7.13, -5.58, -4.42, -3.17, -2.36, -0.42, 0.32, 1.32, 1.91, 2.23, 2.86, 3.02, 3.55, 3.56, 3.57, 3.64, 3.66, 6.94, 7.30, 8.45, 30.73, 31.74, 33.42. MALDI-TOF MS (alpha/MeOH): *m/z* = 668 $[M+Na]^+$ (100%), 684 $[M+K]^+$ (45%). IR (FT-IR**)** ν (cm^{-1}): 3368 (O-H stretch); 2993, 2869 (sp^3 C-H stretch); 1602 (C=O stretch); 1516 (C=C stretch); 1390 (C-H bend); 1275, 1248, 1164 (C-O stretch); 1084, 1030 (C-N stretch); 839, 718 638 (sp^2 C-H bend).

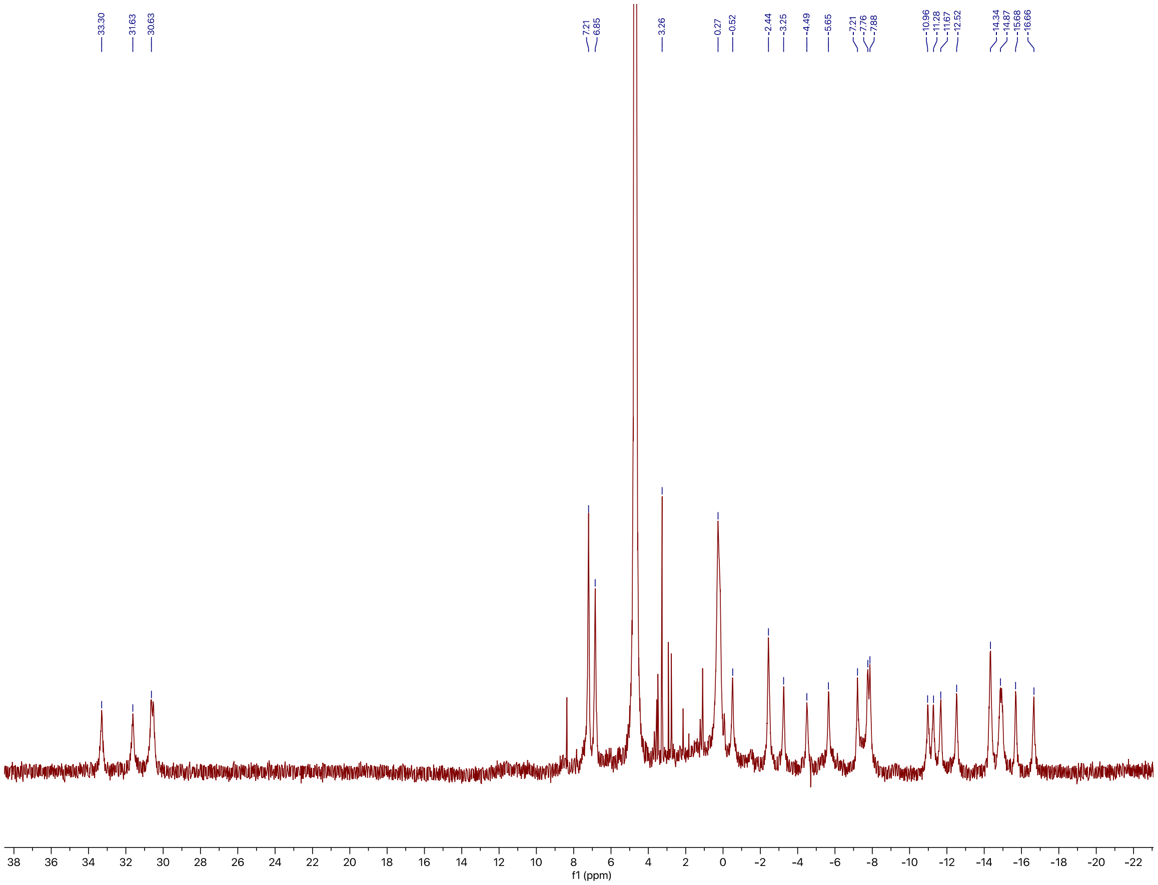

Figure S1.32: Full ^{1}H NMR spectrum of compound **[Eu]**.

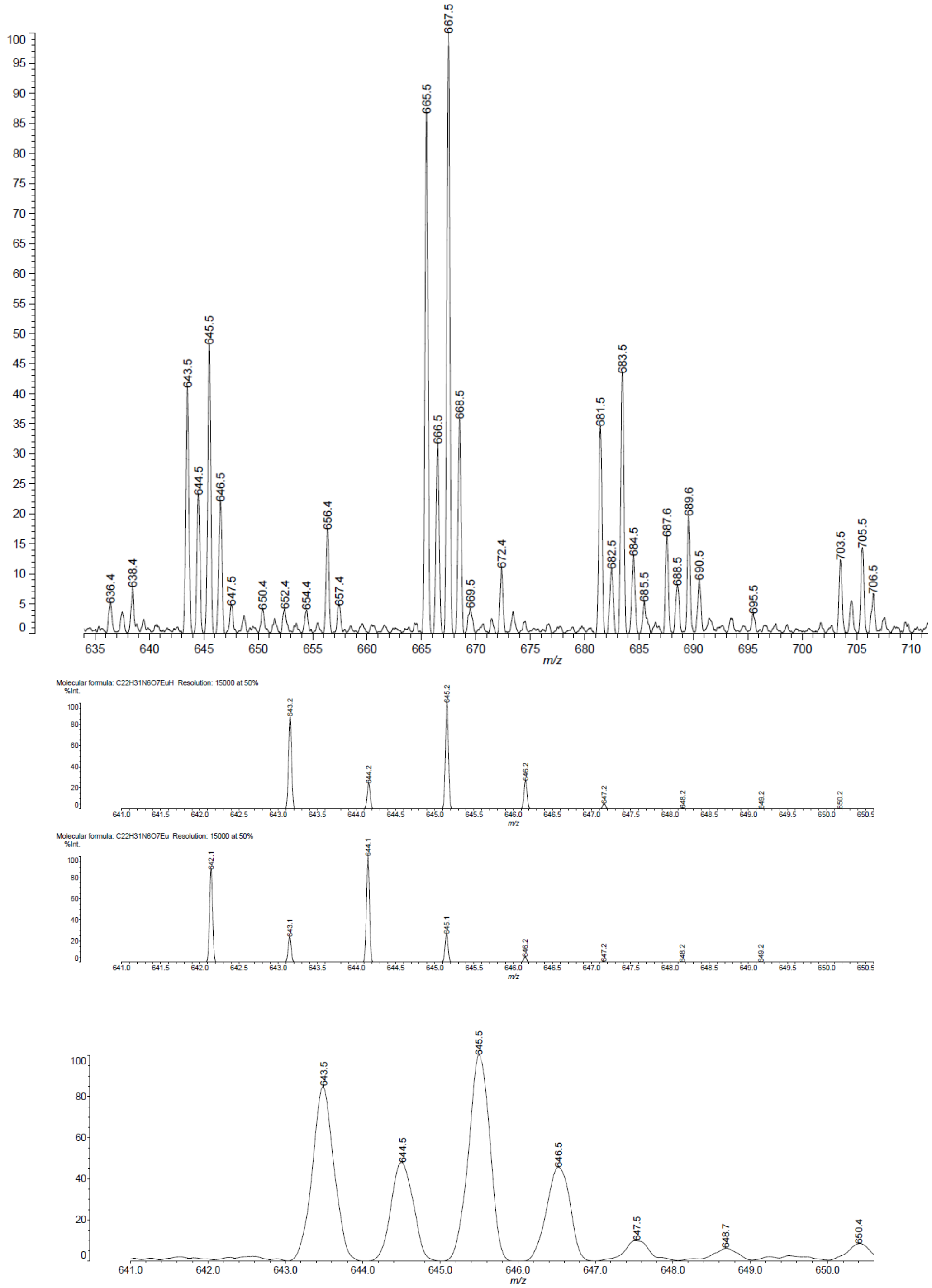

Figure S1.33: Above: MALDI spectra of compound **[Eu]**. Below: Calculated splitting patterns for the [M+H^{+}] (top) and [M]$^{+}$ (middle) adducts compared to the measured spectrum (bottom).

1.11 {Eu(DO3A)}2-DTPA [Eu]2DTPA

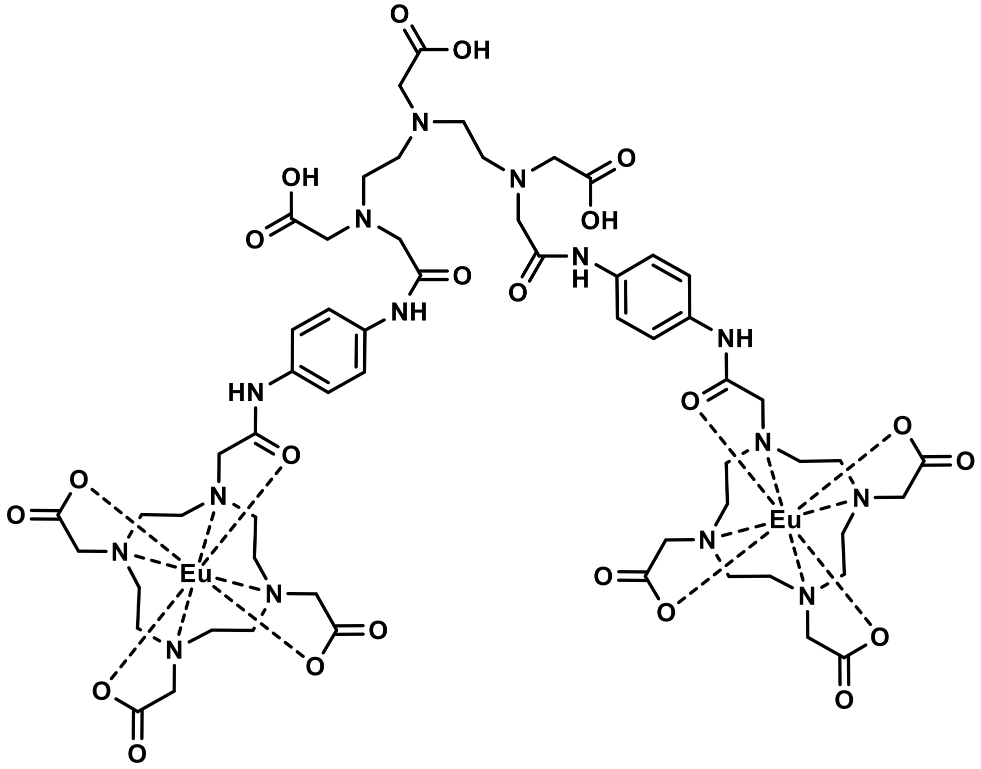

Figure S1.34: Chemical structure of compound **[Eu]2DTPA**.

Under argon, complex **[Eu]** (87.2 mg, 0.14 mmol) was dissolved in dry dimethylformamide (DMF) and potassium carbonate (4 eq.) was added. Diethylenetriamine pentaacetic acid (DTPA) anhydride (19.4 mg, 0.054 mmol) was dissolved in anhydrous DMF and added to the stirring reaction. The reaction mixture was then heated to 55 °C for 14 days under argon. The solution was opened to air, cooled and filtered. The filtrate was reduced *in vacuo* and the residue re-dissolved in methanol and then precipitated with diethyl ether and placed in a freezer (-18 °C) overnight. The beige solid was decanted, washed with diethyl ether and dried thoroughly under vacuum. The desired complex was isolated as a hygroscopic beige solid (31.2 mg, 35 %).

^{1}H NMR (500 MHz, D_2O) δ -16.78, -15.73, -14.63, -13.82, -12.20, -11.32, -11.04, -7.82, -7.48, -5.43, -4.00, -3.48, -2.76, -1.42, -0.51, 0.30, 0.84, 1.24, 2.86, 2.92, 3.04, 3.26, 3.33, 3.51, 3.56, 3.73, 7.78, 8.21, 30.09, 31.10, 33.34. MALDI-TOF MS (alpha/MeOH): *m/z* = 1705 [M+IPA+H] (100%), 1683 $[M+K]^+$ (94%), 1667 $[M+Na]^+$ (68%), 1645 $[M+H]^+$ (47%). IR (FT-IR**)** ν (cm^{-1}): 3184 (O-H stretch); 1584 (C=O stretch); 1442, 1347 (C-H bend); 1237 (C-O stretch), 1186, 1060, 1010 (N-H bend); 840, 835, 689 (sp^2 C-H bend).

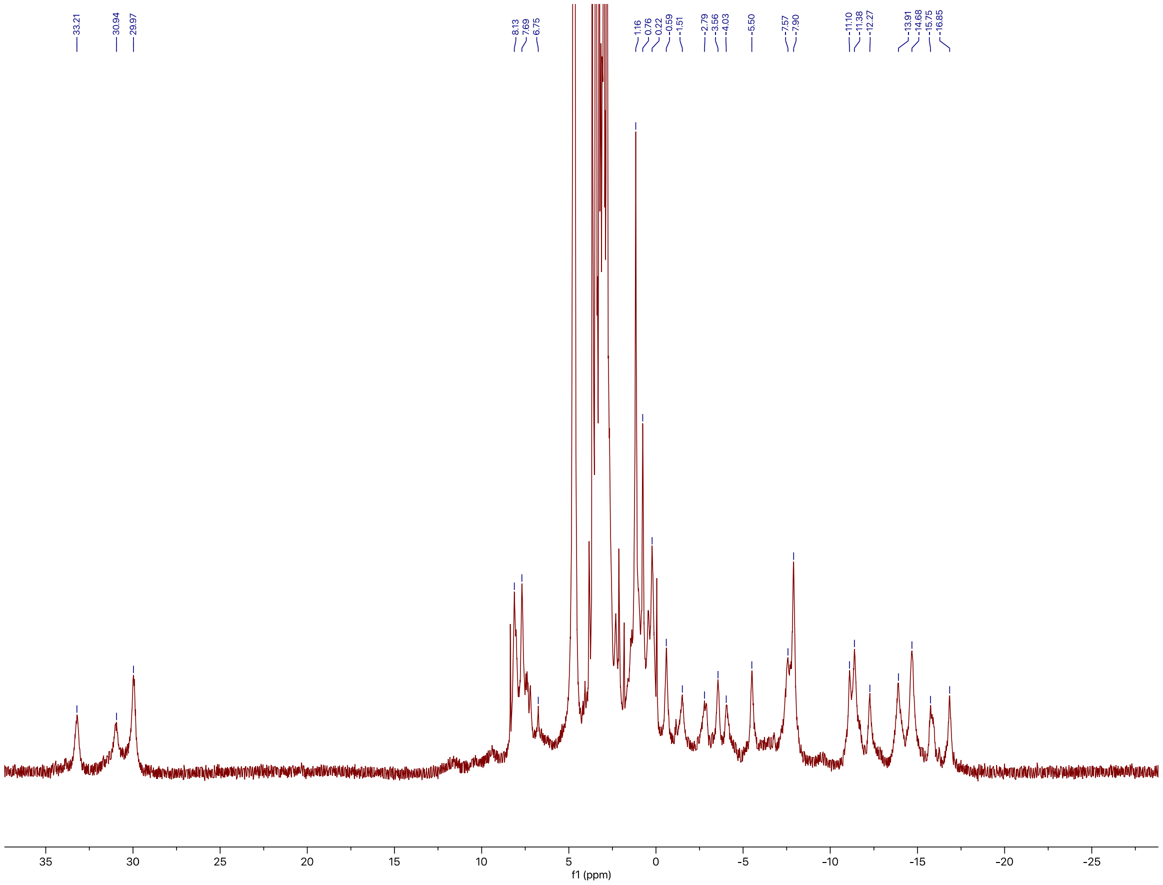

Figure S1.35: Full ^{1}H NMR spectrum of compound **[Eu]$_2$DTPA**.

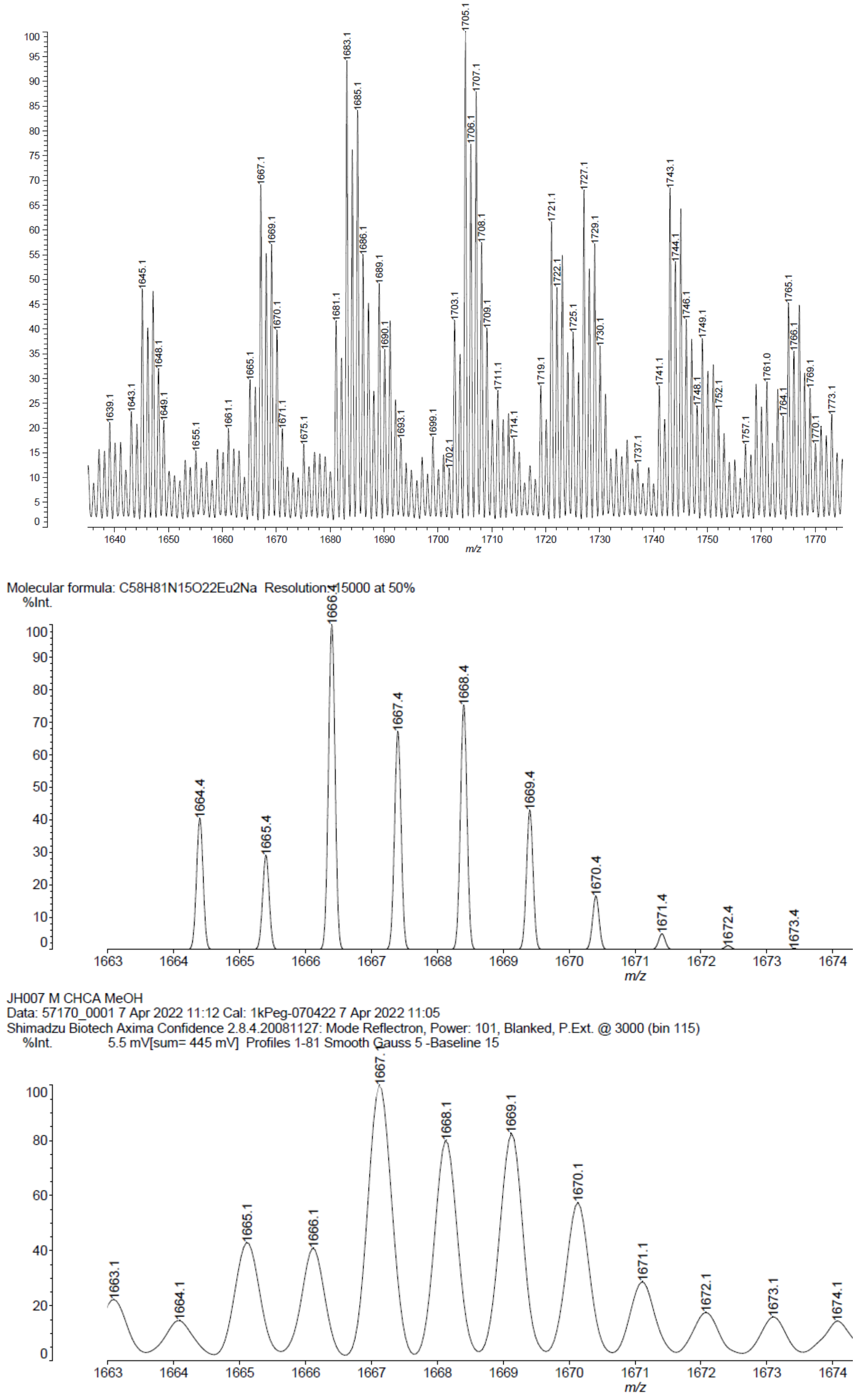

Figure S1.36: Above: MALDI spectrum of compound **[Eu]$_2$DTPA**. Below: calculated (top) and measured (bottom) splitting pattern for the $[M+Na]^+$ adduct.

1.12 {Eu(DO3A)}2-{Tb(DTPA)} [Eu2Tb]

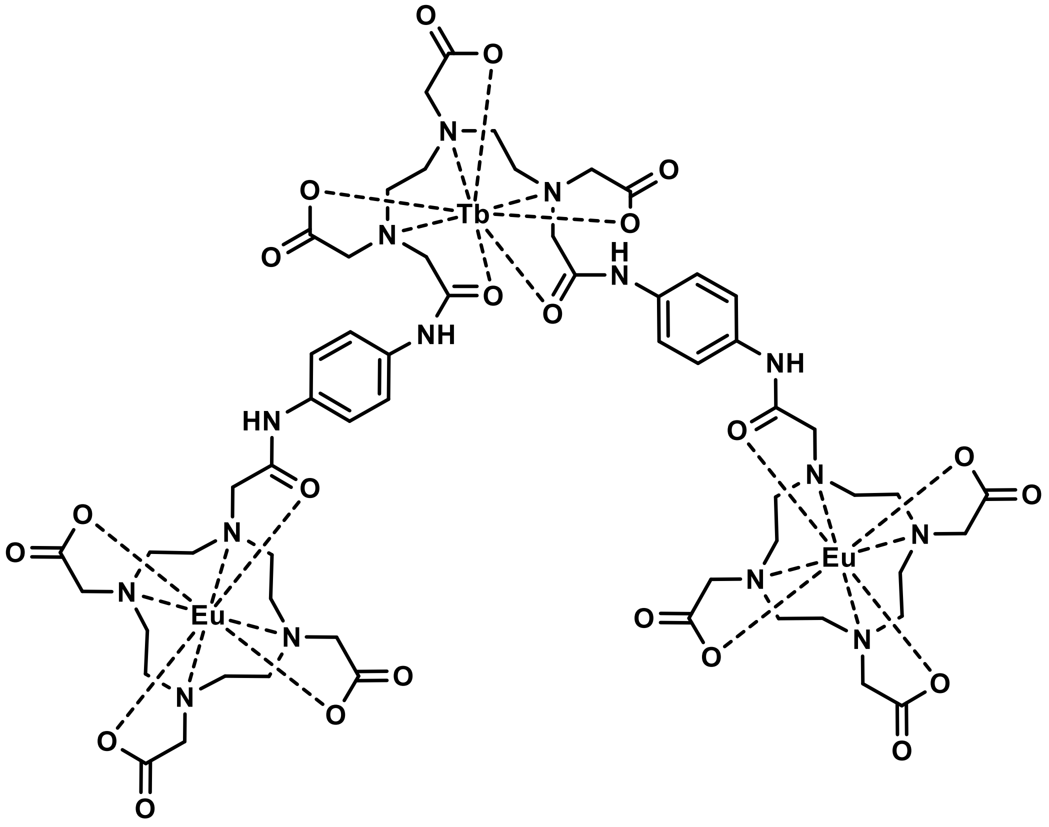

Figure S1.37: Chemical structure of compound **[Eu2Tb]**.

[Eu]2DTPA (41.1 mg, 0.025 mmol) was reacted with $Tb(OTf)_3$ (16.4 mg, 0.027 mmol) following the general complexation procedure outlined in **1.1** to result in a 42% yield (19 mg).

^1H NMR (500 MHz, MeOD) δ -20.32, -18.27, -17.93, -17.70, -16.76, -15.53, -13.84, -13.45, -12.77, -11.20, -9.55, -6.44, -5.09, -3.44, -2.41, -1.66, -1.24, -0.53, 33.93, 34.75, 35.95, 38.68, 39.11. MALDI-TOF: *m/z* = 1862 $[M+Na+K-H]^+$ (100%), 1839 $[M+K]^+$ (88%), 1823 $[M+Na]^+$ (85%), 1801 $[M+H]^+$ (64%), 1845 $[M+2Na-H]^+$ (53%). IR (FT-IR) ν (cm^{-1}): 3256 (O-H stretch); 2987, 2777 (sp^3 C-H stretch); 1598 (C=O stretch); 1515 (C=C stretch); 1356, 1322, 1256 (C-N stretch); 1163, 1086, 1030 (C-O stretch); 936, 842, 638 (sp^2 C-H bend).

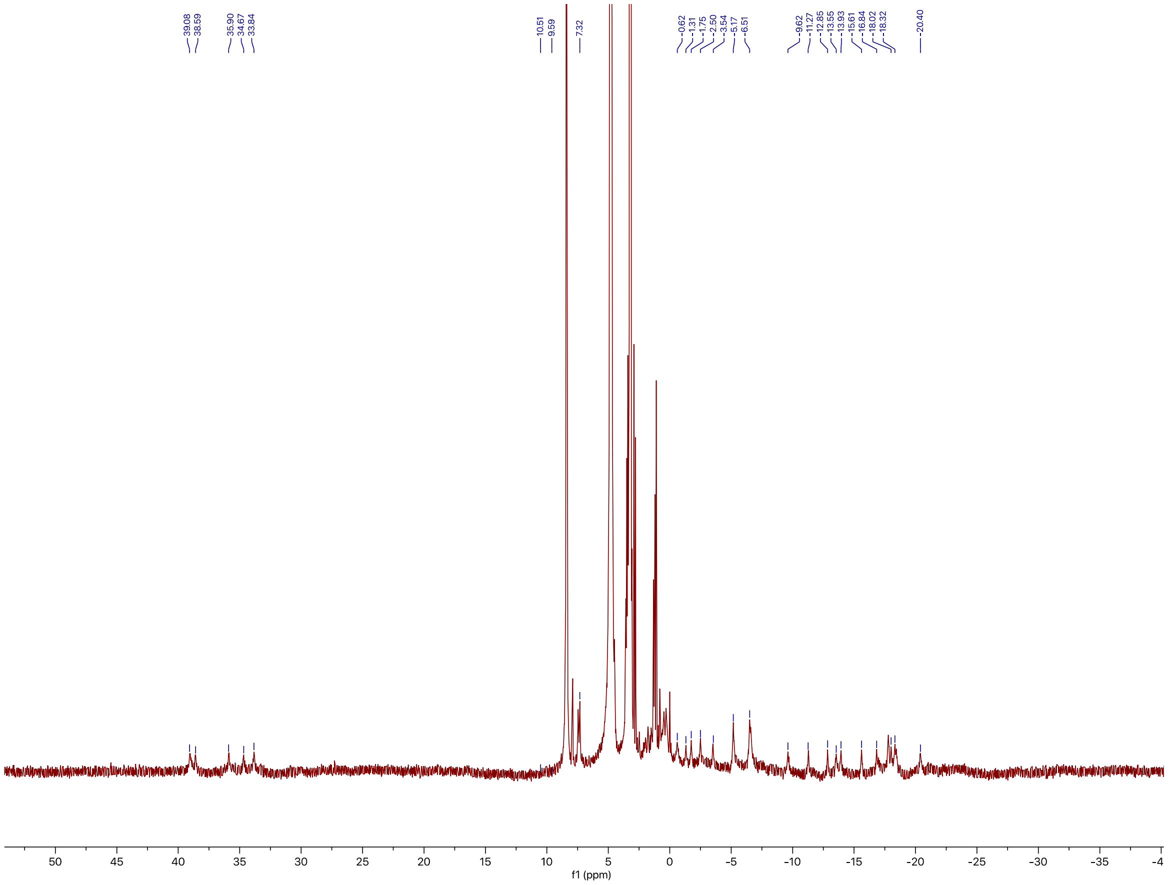

Figure S1.38: Full ^{1}H NMR spectrum of compound **[Eu2Tb]**. Note that the majority of the Tb(III) resonances are too broad to be identified at this and larger spectral widths.

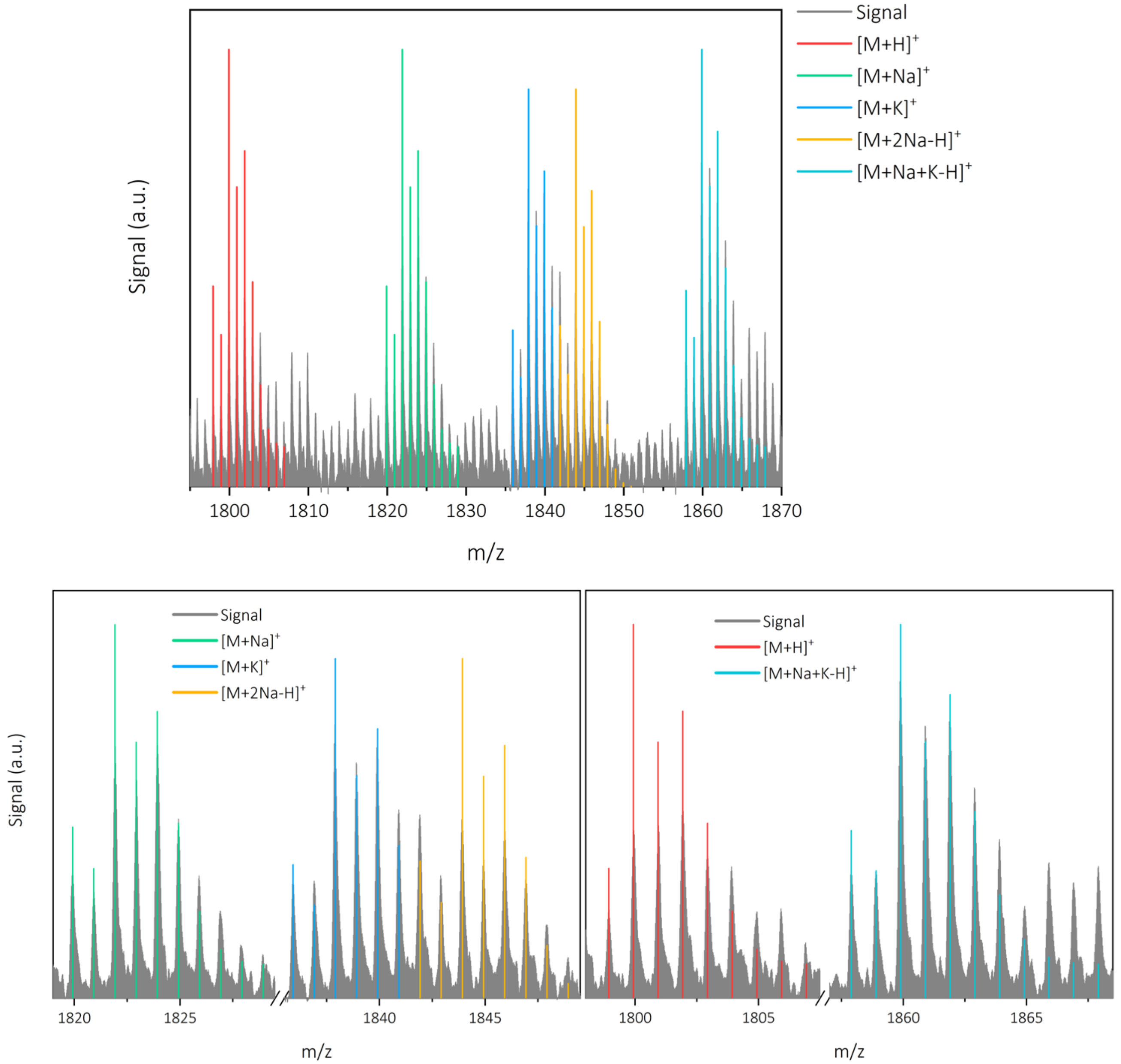

Figure S1.39: Above: MALDI spectrum of compound **[Eu2Tb]** with calculated splitting patterns of $[M+H]^+$ (red), $[M+Na]^+$ (green), $[M+K]^+$ (blue), $[M+2Na-H]^+$ (orange) and $[M+Na+K-H]^+$ (teal) adducts. Below: cropped spectra highlighting overlap between calculated and experimental data.

1.13 {Yb(DO3A)}$_2$-{Eu(DTPA)} [Yb$_2$Eu]

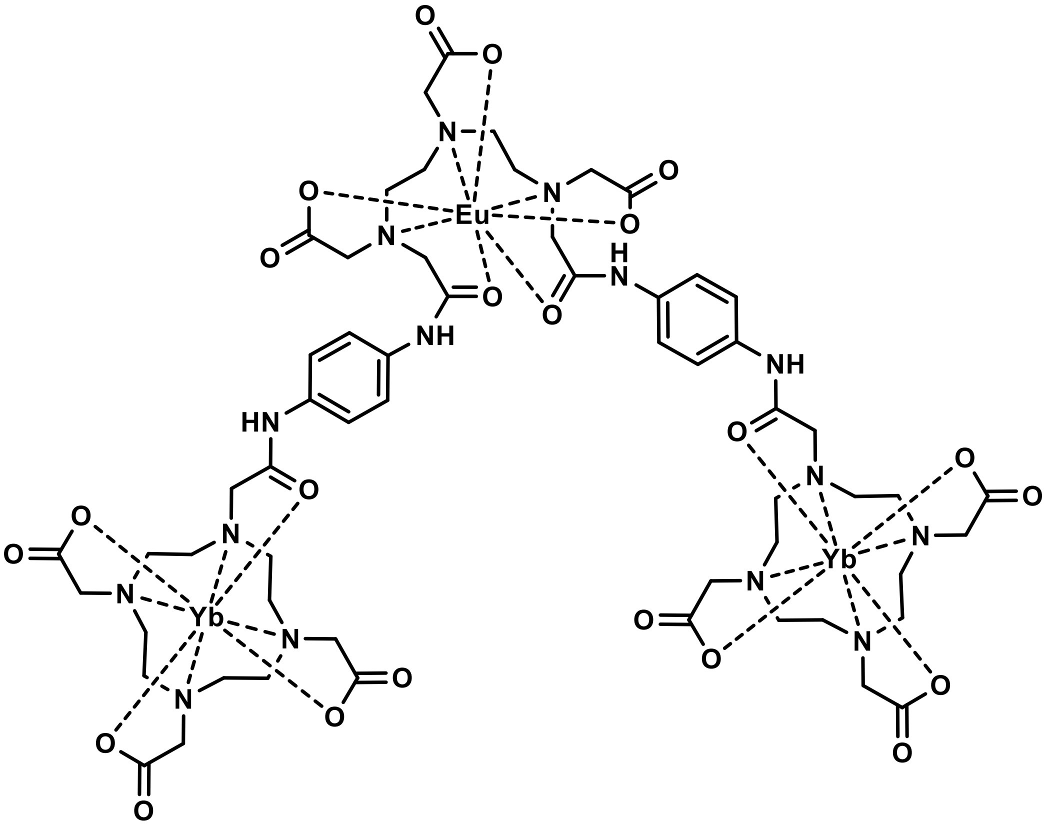

Figure S1.40: Chemical structure of compound **[Yb$_2$Eu]**.

[Yb]$_2$DTPA (42.8 mg, 0.025 mmol) was reacted with Eu(OTf)$_3$ (16.8 mg, 0.028 mmol) following the general complexation procedure outlined in **1.1** to result in a 48% yield (22 mg).

^{1}H NMR (500 MHz, MeOD) δ -97.44, -85.57, -83.41, -66.71, -51.16, -49.70, -46.35, -42.87, -17.21, -16.70, -16.01, -13.70, -12.13, -9.70, -5.41, -3.40, -2.62, -0.72, 7.59, 8.58, 10.17, 13.08, 14.76, 17.17, 18.74, 19.74, 23.27, 25.56, 28.20, 30.01, 33.03, 36.09, 151.16.

MALDI-TOF: *m/z* = 1858 [M+Na]$^+$ (100%), 1875 [M+K]$^+$ (77%), 1837 [M+H]$^+$ (75%), 1896 [M+Na+K-H]$^+$ (74%), 1880 [M+2Na-H]$^+$ (58%). IR (FT-IR) ν (cm^{-1}): 32378 (O-H stretch); 2976, 2922 (sp^3 C-H stretch); 1593 (C=O stretch); 1515 (C=C stretch); 1401, 1323, 1253 (C-N stretch); 1164, 1088, 1030 (C-O stretch); 931, 718, 638 (sp^2 C-H bend).

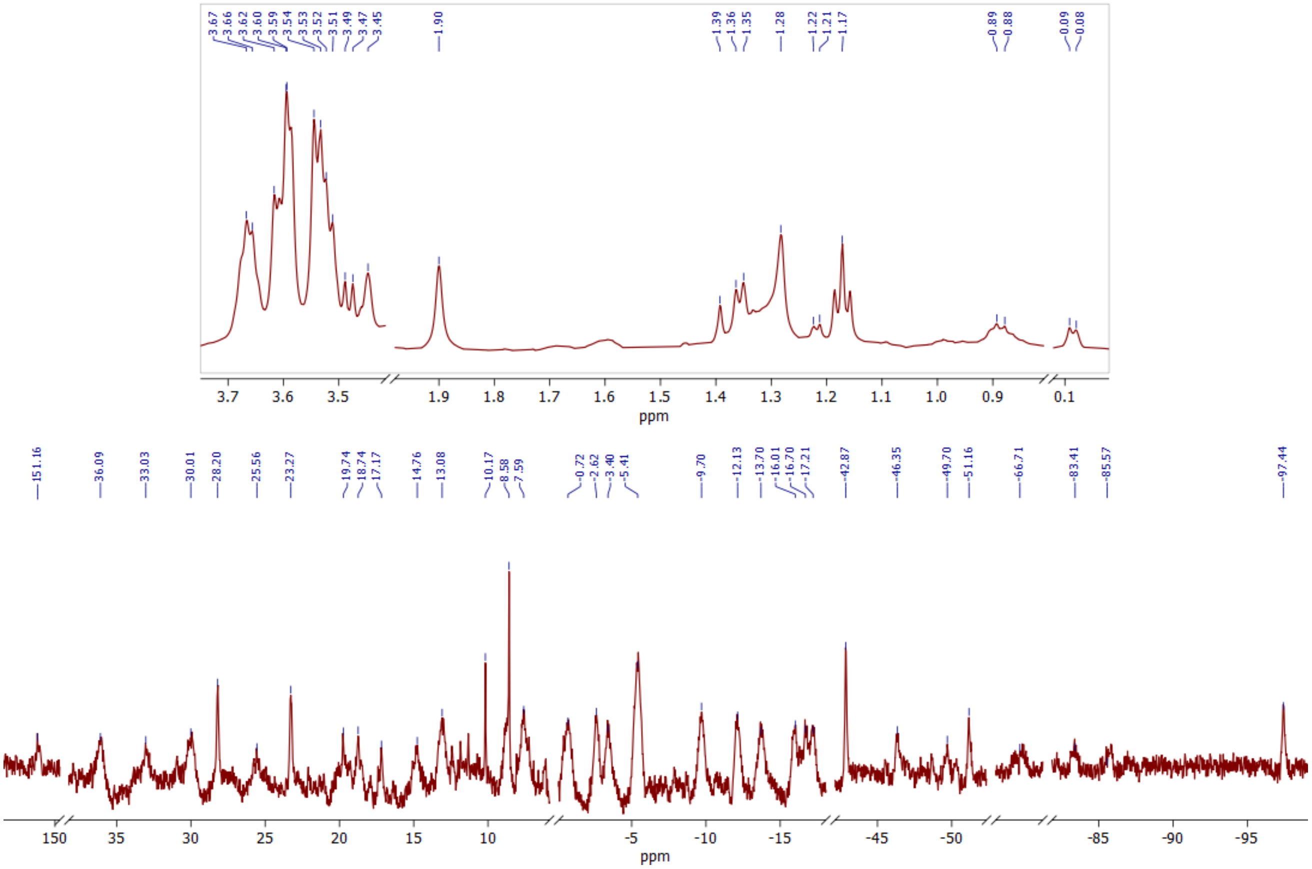

Figure S1.41a: ^{1}H NMR spectrum of compound **[Yb$_2$Eu]**. Regions between -80 – -70, -65 – -52, -40 – -18, 0 – 6 and 38 – 150 ppm omitted for clarity.

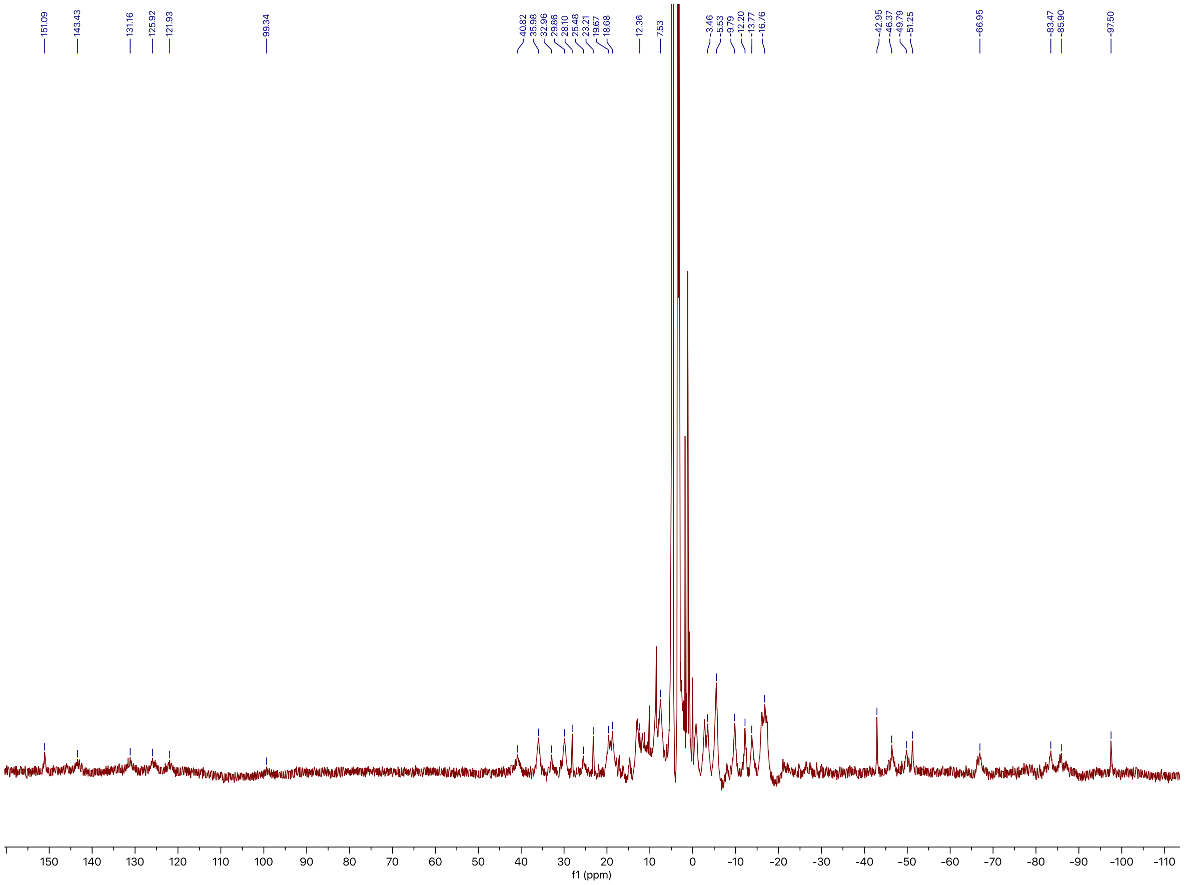

Figure S1.41b: Full ^{1}H NMR spectrum of compound **[Yb_2Eu]**.

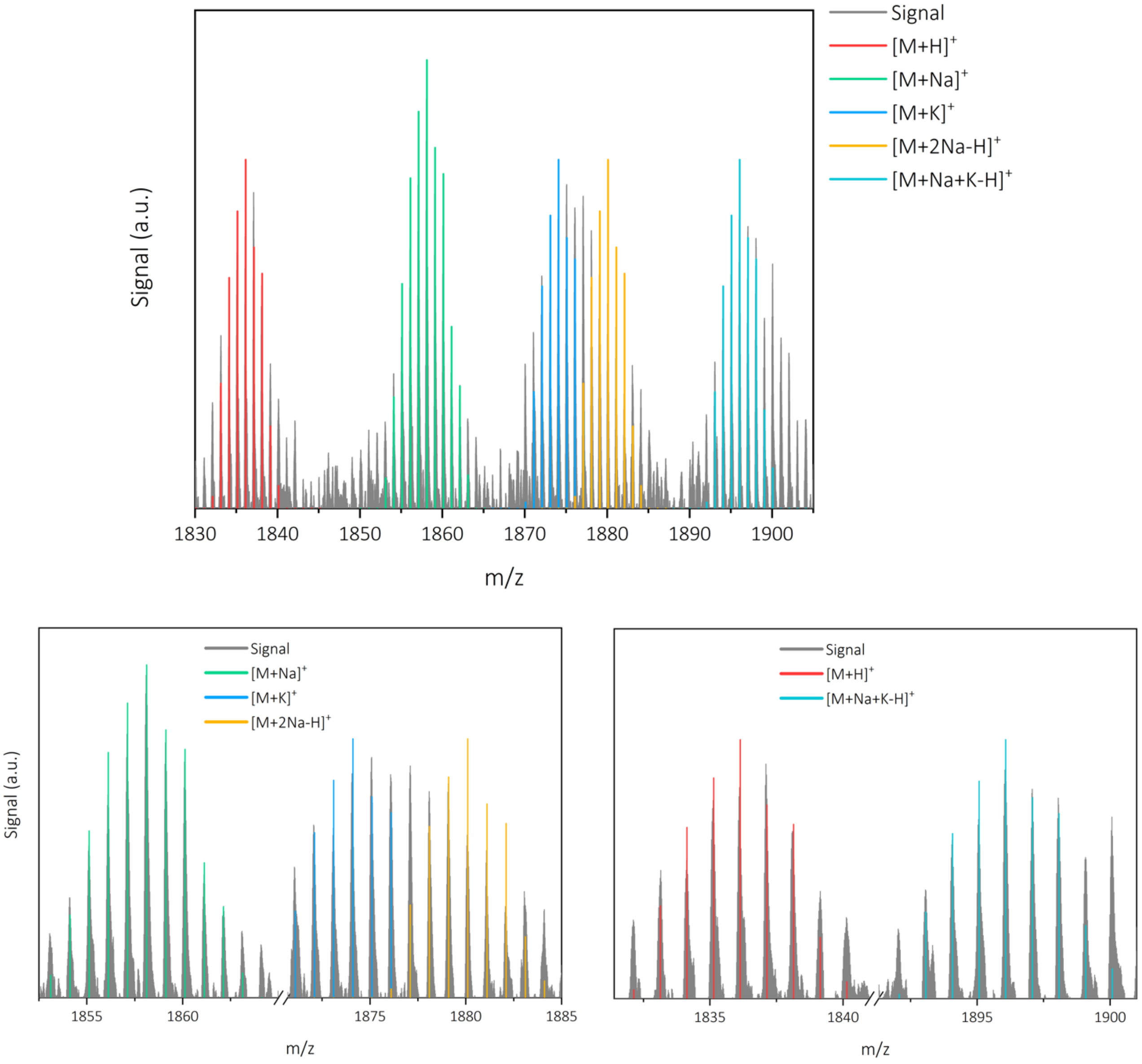

Figure S1.42: Above: MALDI spectrum of compound **[Yb$_2$Eu]** with calculated splitting patterns of $[M+H]^+$ (red), $[M+Na]^+$ (green), $[M+K]^+$ (blue), $[M+2Na\text{-}H]^+$ (orange) and $[M+Na+K\text{-}H]^+$ (teal) adducts. Below: cropped spectra highlighting overlap between calculated and experimental data.

2 Luminescence data

2.1 Equations

$$\tau_{avg} = \sum_{i=1}^{n} \frac{A_i {\tau_i}^2}{A_i \tau_i}$$

Equation S1: Calculation of the average lifetime τ_{avg} for multi-exponential lifetimes using the relative amplitude A and lifetime τ for each component i.

$$\%\, contribution = \left(\frac{A_i \tau_i}{\sum_{i=1}^{n} A_i \tau_i} \right) x\ 100$$

Equation S2: Calculation of the relative percentage contribution of each decay component in a multi-exponential decay curve, used to analyze lifetime data.

$$q = A'\left(k_{H_2O} - k_{D_2O} - (B + n_{N-H})\right)$$

Equation S3: Calculation of the number of inner sphere solvent molecules q where k is the radiative rate constant ($1/\tau$) in the respective solvent. The proportionality constant A' and correction factor B for each Ln^{III} is summarized in table S1. The additional term for quenching by amide N-H oscillators is required for Eu^{III} only.

Table S1: Values employed in the calculation of inner sphere hydration number q (Beeby *et al.*, 1999). The number of amide N-H oscillators n = 1 for the DO3A binding site and 2 for the DTPA site.

Ln^{III}	A'	B	N-H
**Eu**	1.2 ms	0.25 ms^{-1}	0.075 ms^{-1}
**Tb**	5 ms	0.06 ms^{-1}	-
**Yb**	1 µs	0.20 $µs^{-1}$	-

2.2 [Yb_2Tb] spectra

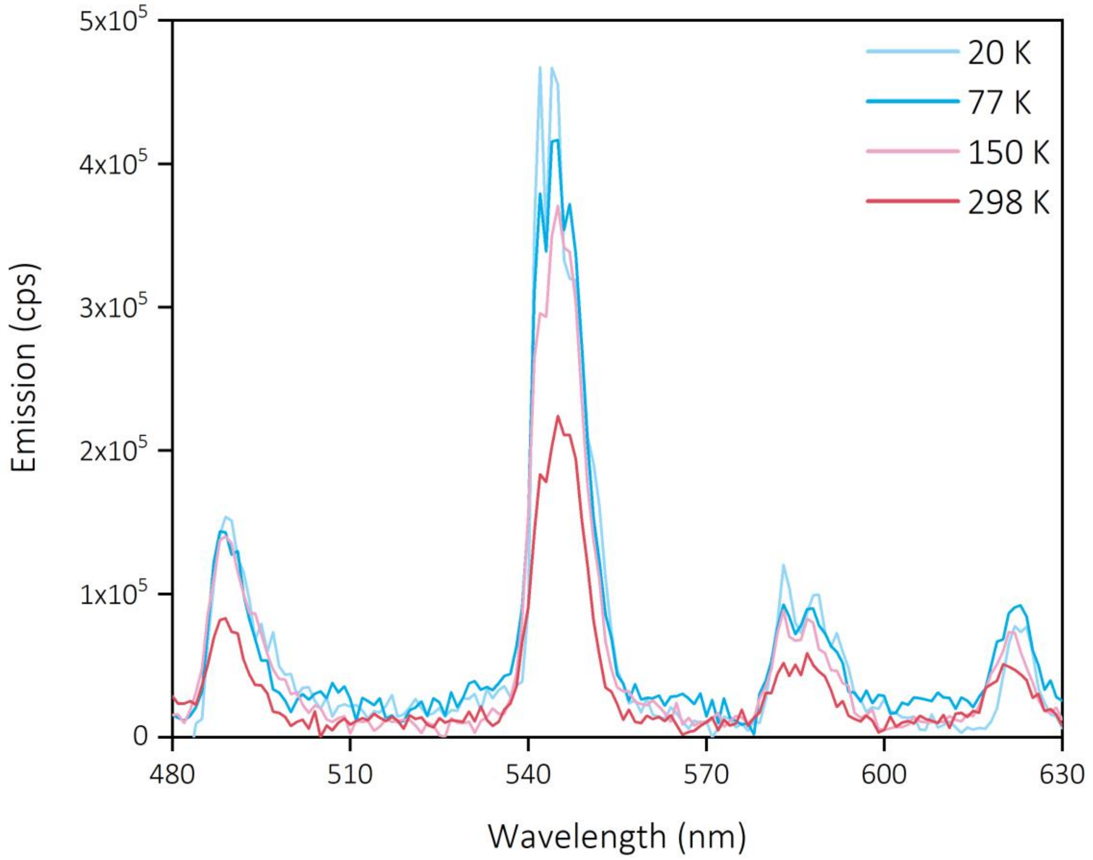

Figure S2.1: Solid-state variable temperature emission spectra of Tb^{III} in [Yb_2Tb] at 20 K (light blue), 77 K (dark blue), 150 K (light red) and 298 K (dark red) (λ_{ex} = 366 nm).

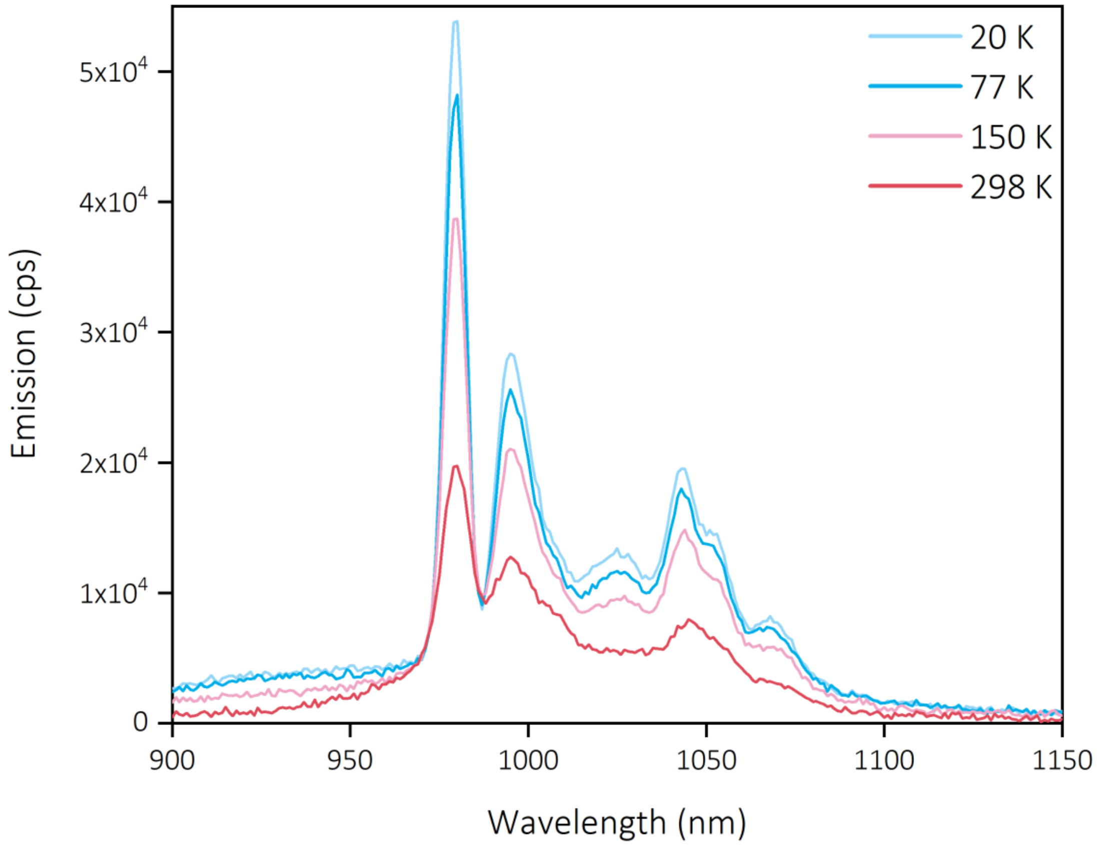

Figure S2.2: Solid-state variable temperature emission spectra of Yb^{III} in [Yb_2Tb] at 20 K (light blue), 77 K (dark blue), 150 K (light red) and 298 K (dark red) (λ_{ex} = 366 nm).

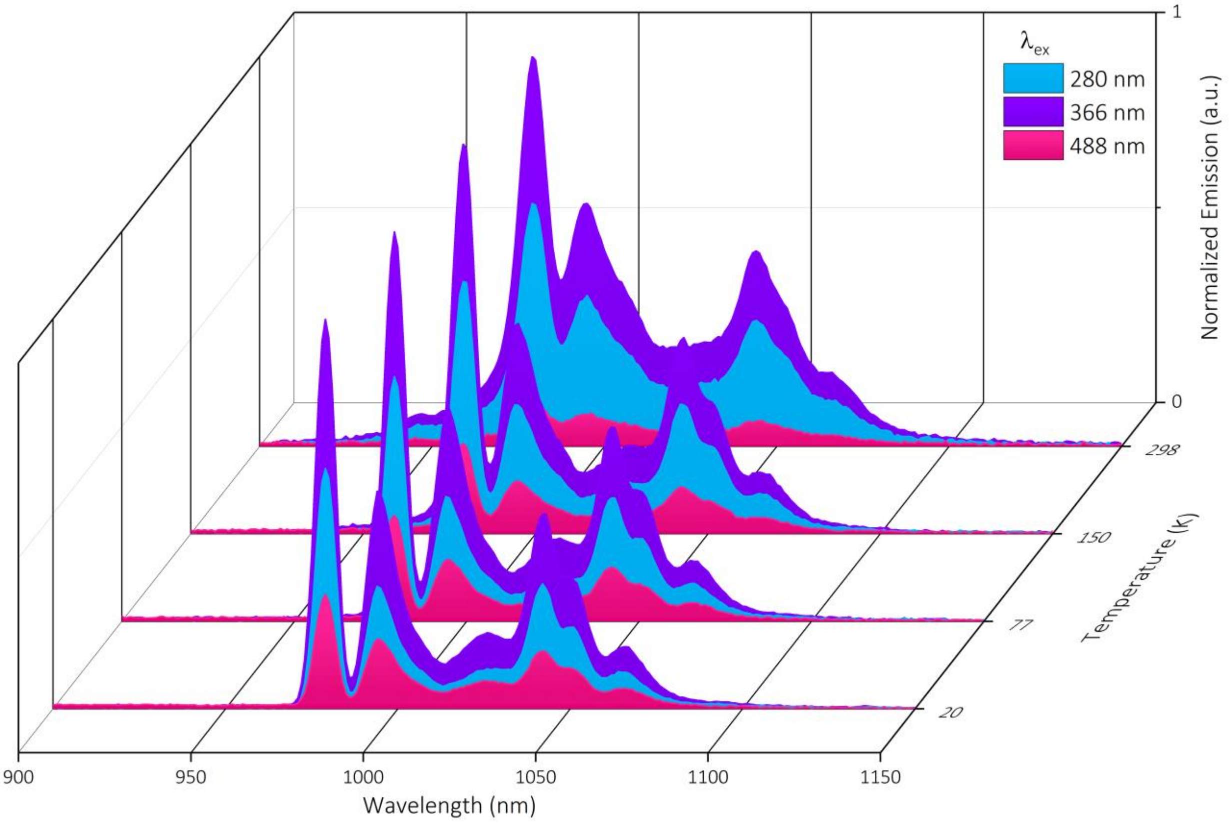

Figure S2.3: Normalized solid-state emission spectra of DO3A complex [Yb] under 280 nm (blue), 366 nm (purple) and 488 nm (pink) excitation. Data are normalized at each temperature relative to the emission maximum (~980 nm @ λ_{ex} = 366 nm).

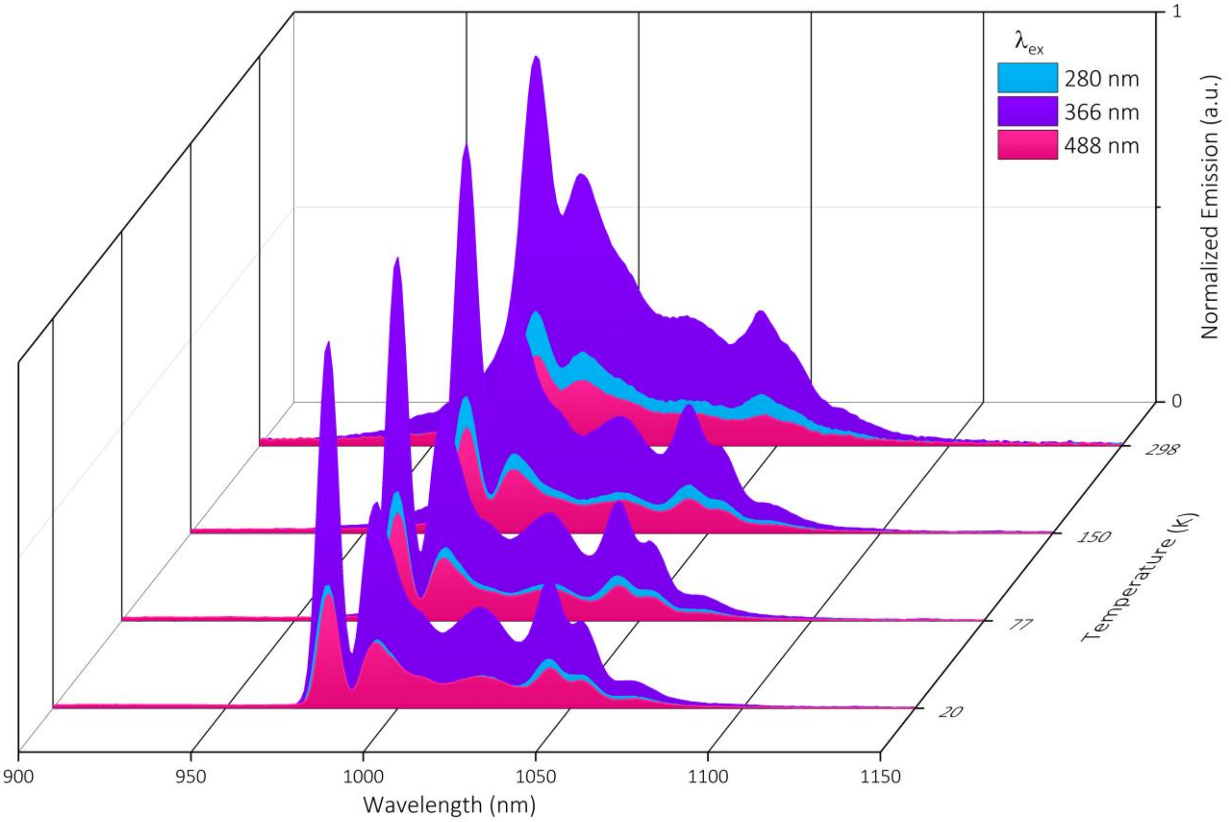

Figure S2.4: Normalized solid-state emission spectra of DTPA complex $[Yb]_2DTPA$ under 280 nm (blue), 366 nm (purple) and 488 nm (pink) excitation. Data are normalized at each temperature relative to the emission maximum (~980 nm @ λ_{ex} = 366 nm).

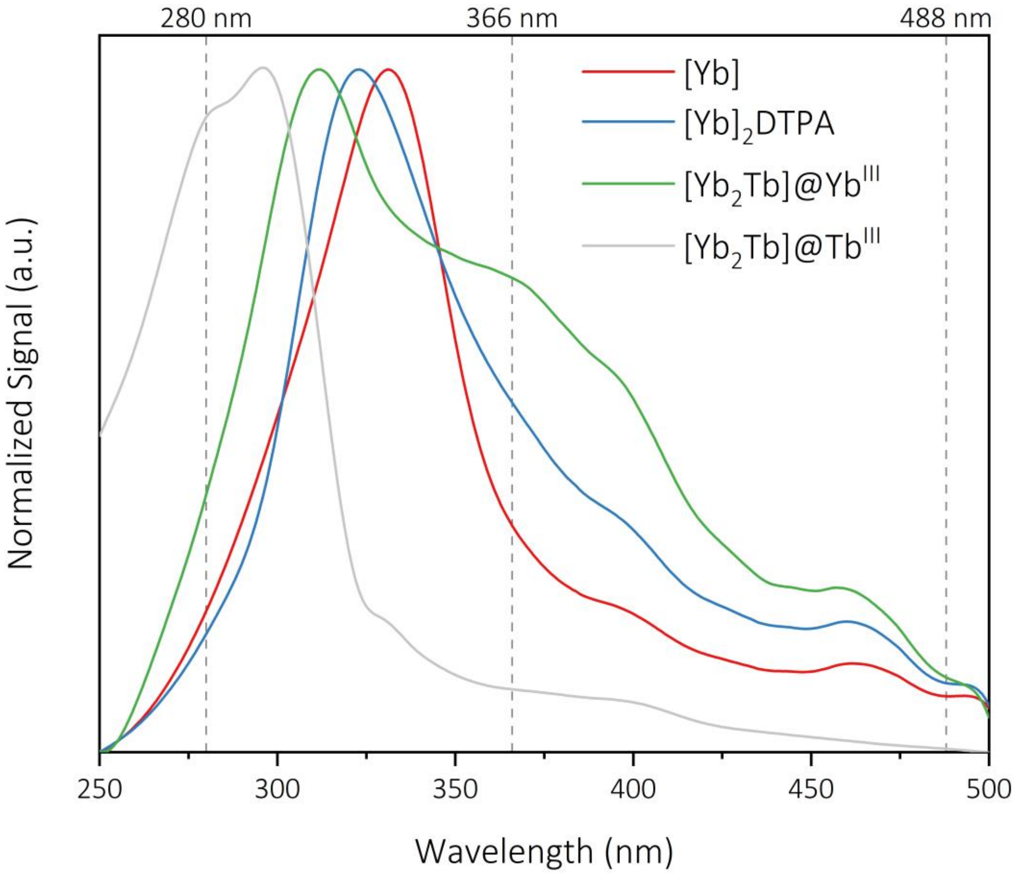

Figure S2.5: Normalized solid-state excitation spectra of Yb^{III} complexes [Yb] (red), $[Yb]_2$DTPA (blue) and target trinuclear bimetallic [Yb_2Tb] (green) at 298 K, λ_{em} = 980 nm. The excitation trace of Tb^{III} from [Yb_2Tb] (λ_{em} = 545 nm, grey) is included for comparison.

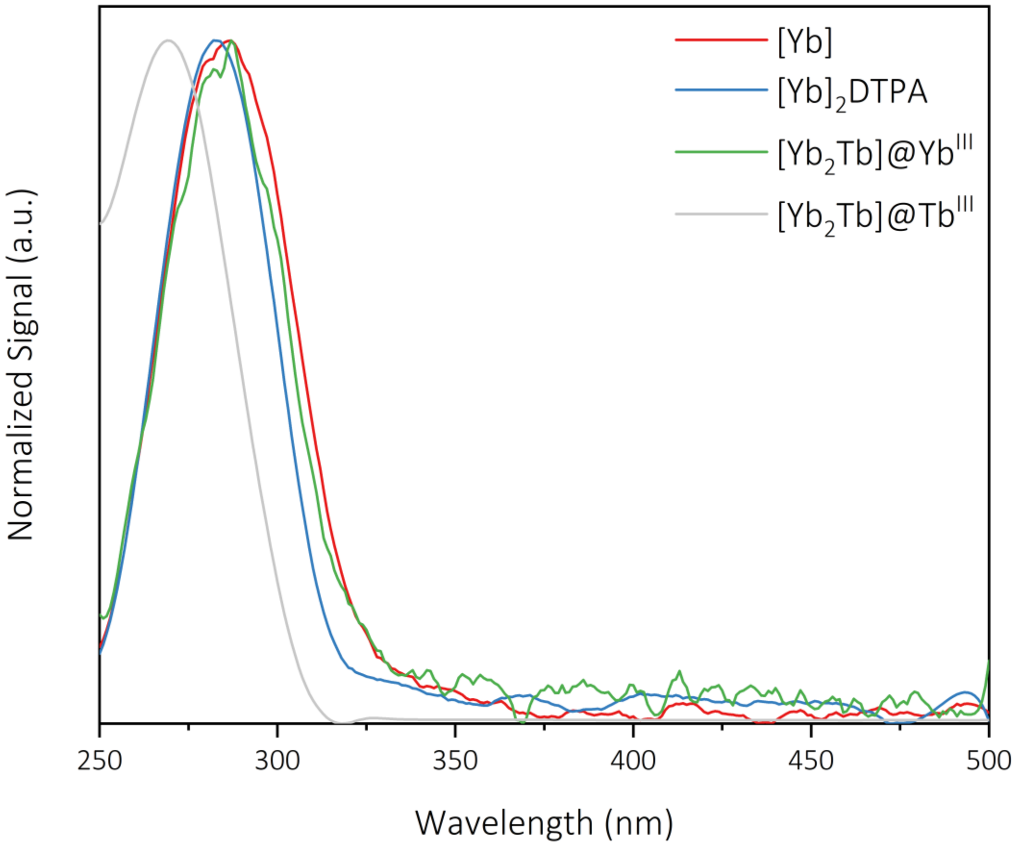

Figure S2.6: Normalized solution-state excitation spectra of Yb^{III} complexes [Yb] (red), $[Yb]_2$DTPA (blue) and target trinuclear bimetallic $[Yb_2Tb]$ (green).

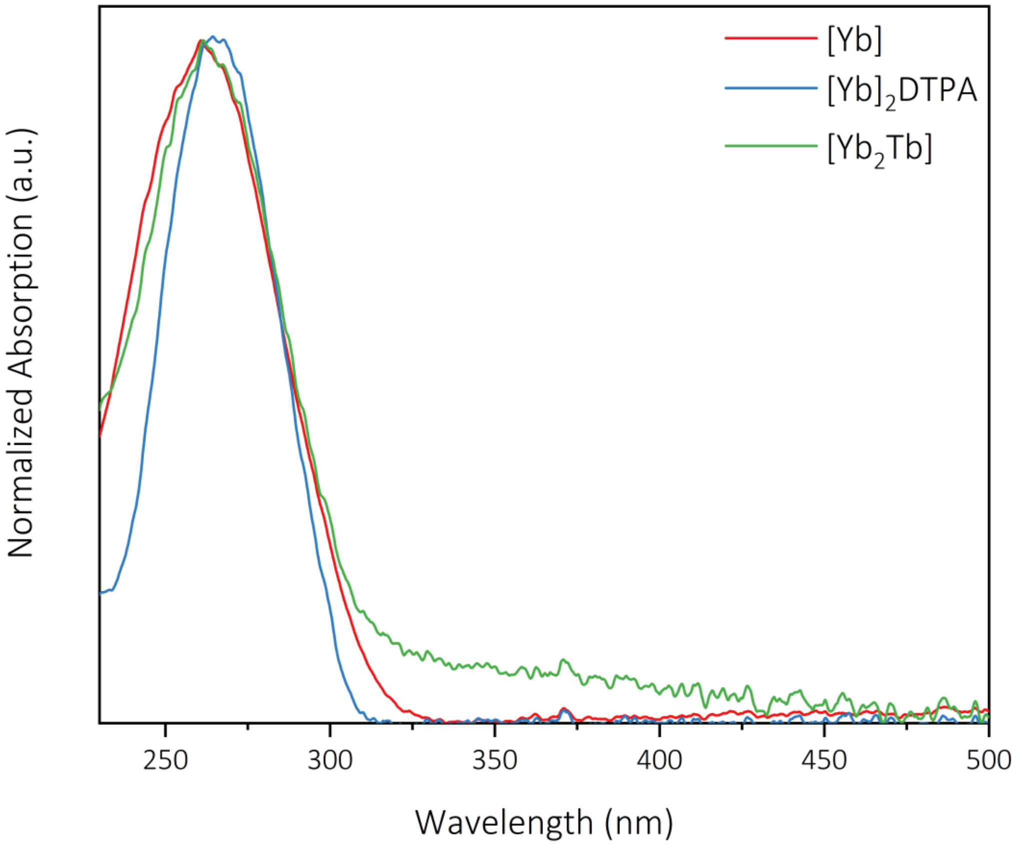

Figure S2.7: UV-VIS absorption spectra of Yb^{III} complexes [Yb] (red), $[Yb]_2DTPA$ (blue) and target trinuclear bimetallic $[Yb_2Tb]$ (green) recorded in D_2O, A = 0.1.

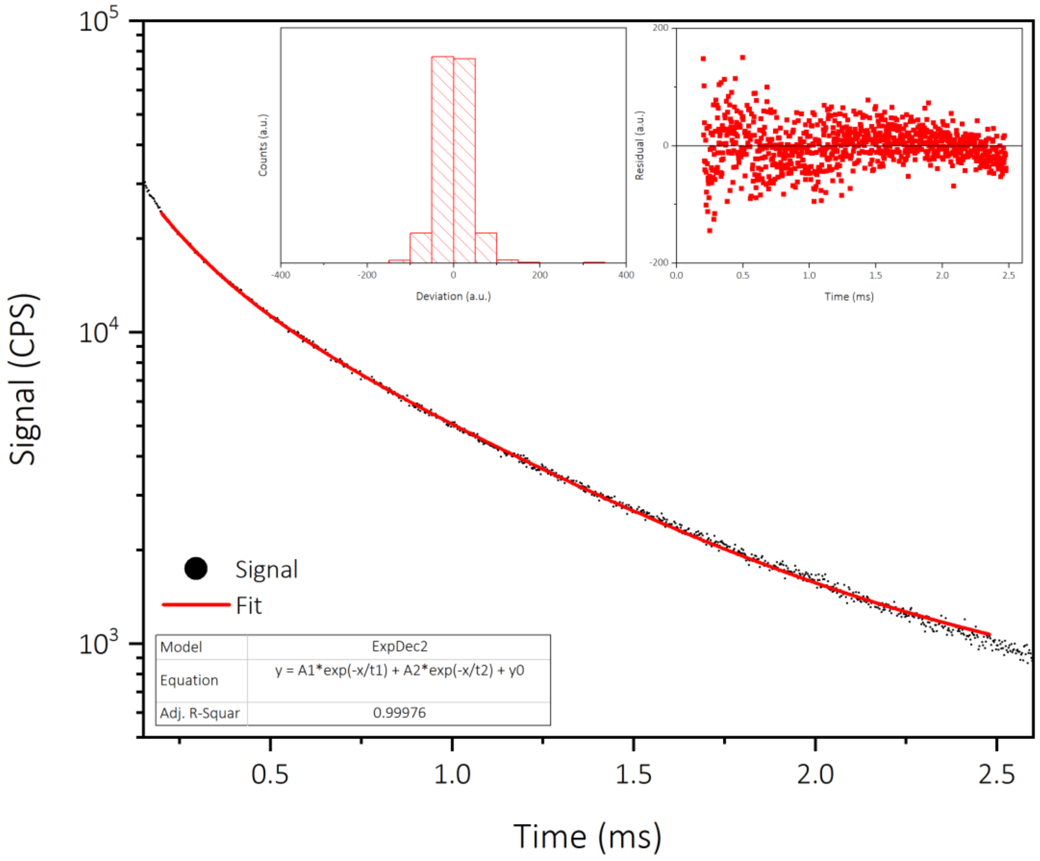

Figure S2.8: Solid-state luminescence decay profile (black) of the Tb^{III} signal in [Yb_2Tb] with corresponding bi-exponential decay fit (red) (λ_{ex} = 280 nm, λ_{em} = 545 nm T = 20 K). Inset: associated residuals of the fit showing both normal distribution (left) and expected error variance (right).

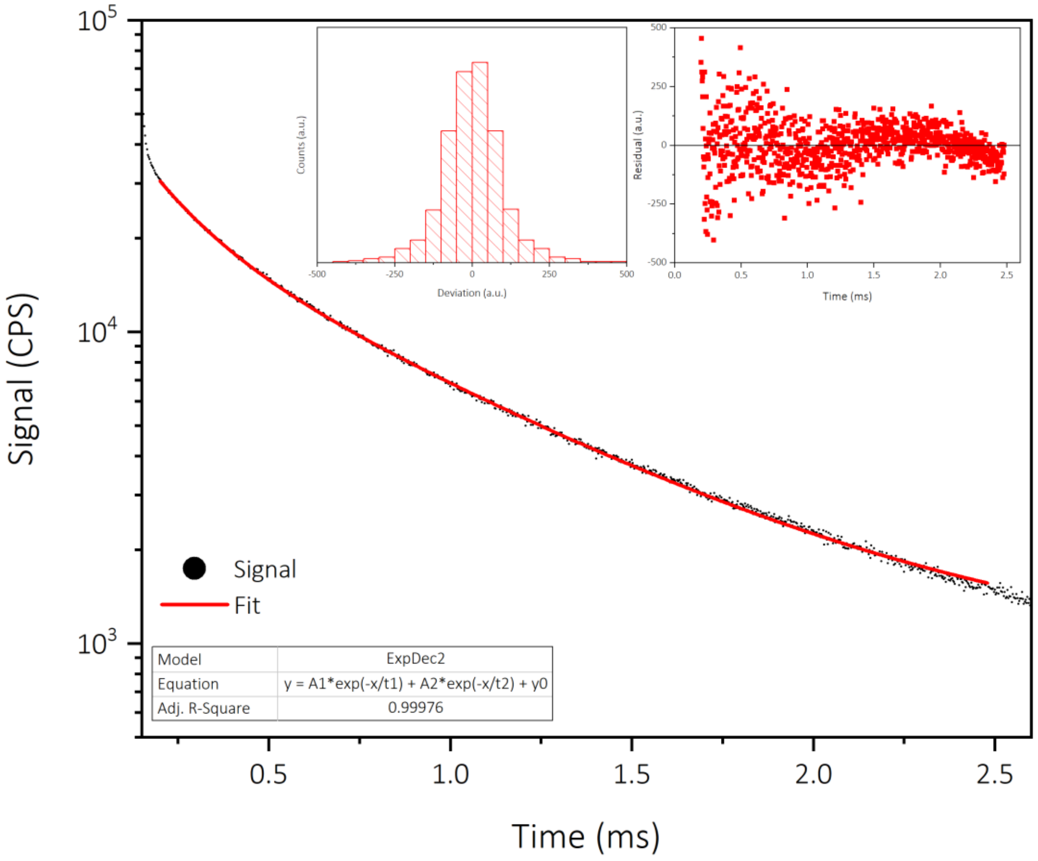

Figure S2.9: Solid-state luminescence decay profile (black) of the Tb^{III} signal in [Yb_2Tb] with corresponding bi-exponential decay fit (red) (λ_{ex} = 366 nm, λ_{em} = 545 nm T = 20 K). Inset: associated residuals of the fit showing both normal distribution (left) and expected error variance (right).

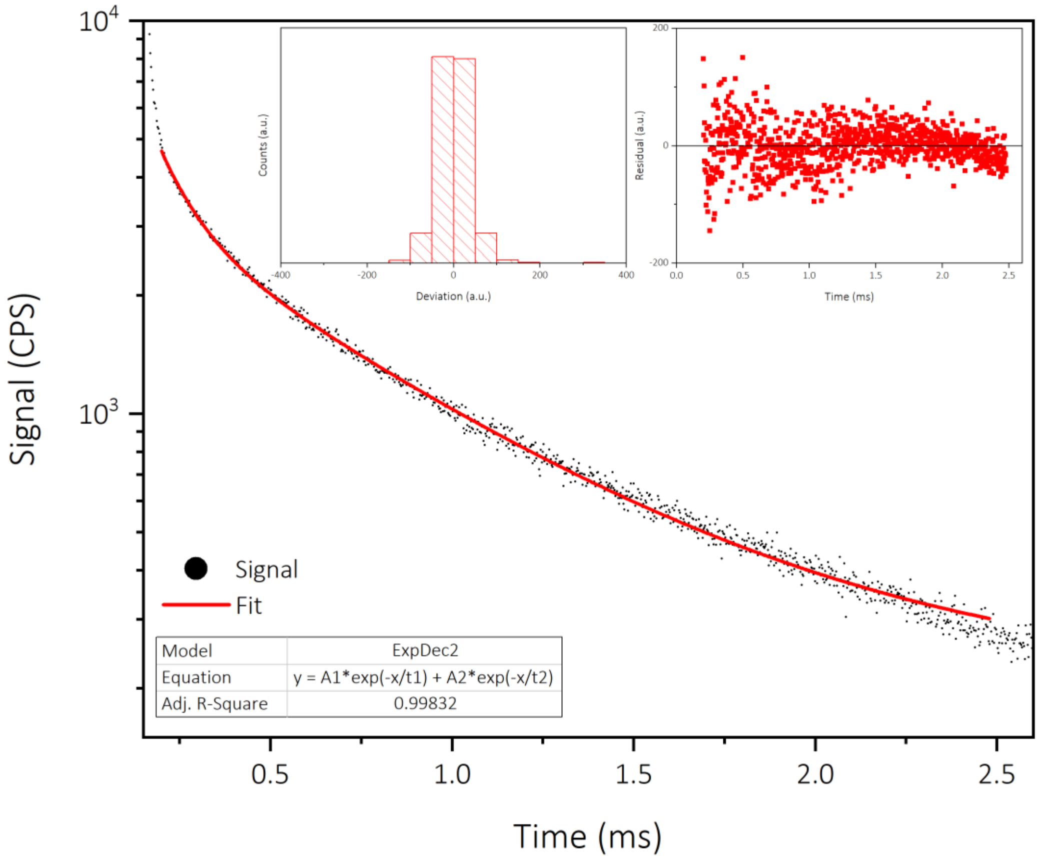

Figure S2.10: Solid-state luminescence decay profile (black) of the Tb^{III} signal in [Yb_2Tb] with corresponding bi-exponential decay fit (red) (λ_{ex} = 488 nm, λ_{em} = 545 nm T = 20 K). Inset: associated residuals of the fit showing both normal distribution (left) and expected error variance (right).

Table S2: List of individual decay components τ_n and their relative contribution (%) towards a bi-exponential decay in solid-state [Yb_2Tb] lifetimes (Tb^{III} λ_{em} = 545 nm), across variable excitation and temperature ranges. Percentages are color-scaled from high (green) to low (red) and highlight the dominance of the long-lived τ_2 component throughout.

	**Tb^{III} lifetimes (ms)**	**20 K**		**77 K**		**150 K**		**298 K**	
		τ_1	τ_2	τ_1	τ_2	τ_1	τ_2	τ_1	τ_2
**λ_{ex} (nm)**	**280**	0.15	0.65	0.17	0.7	0.17	0.73	0.17	0.72
		27%	73%	25%	75%	23%	77%	22%	78%
	**366**	0.15	0.68	0.16	0.69	0.15	0.69	0.14	0.67
		25%	75%	26%	74%	28%	72%	31%	69%
	**488**	0.097	0.66	0.12	0.74	0.12	0.75	0.12	0.75
		35%	65%	30%	70%	28%	72%	27%	73%

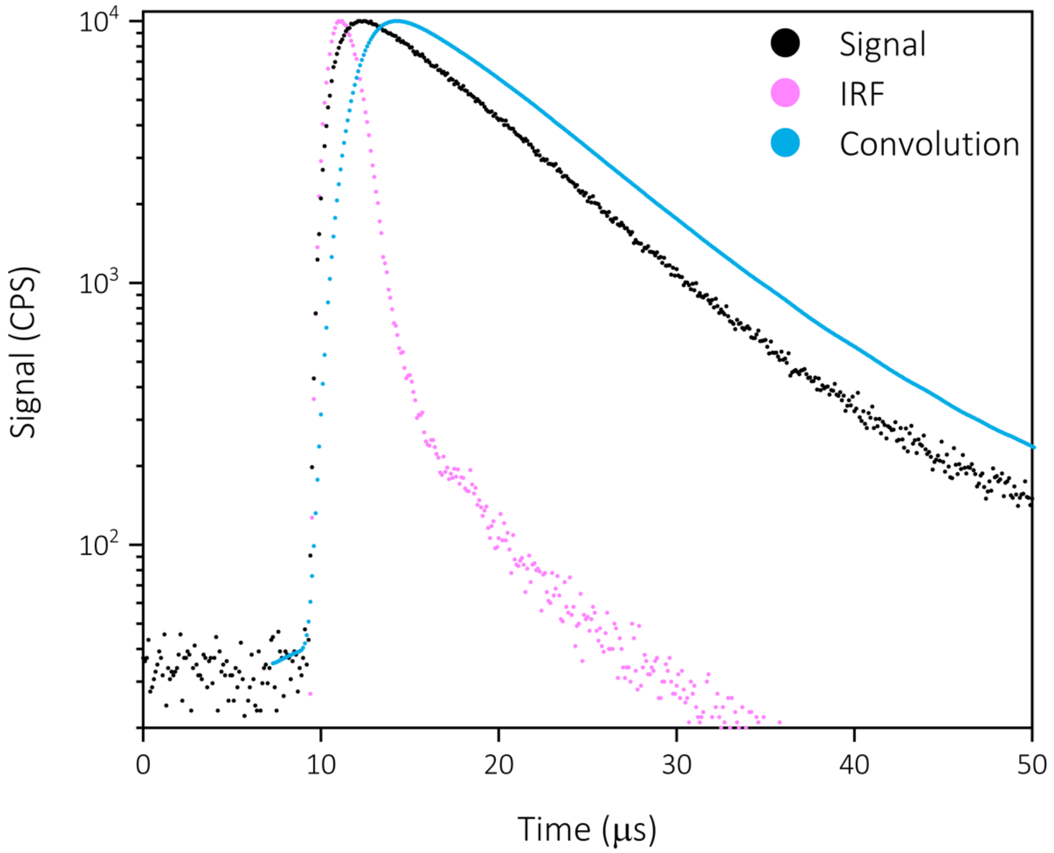

Figure S2.11: Example solid-state luminescence decay profile (black) of a Yb^{III} signal in [Yb_2Tb], compared to the IRF of the microsecond flash lamp (@100 Hz, pink) (λ_{ex} = 280 nm, λ_{em} = 980 nm T = 20 K). These data undergo convolution to produce a signal appropriate for exponential decay fitting (blue).

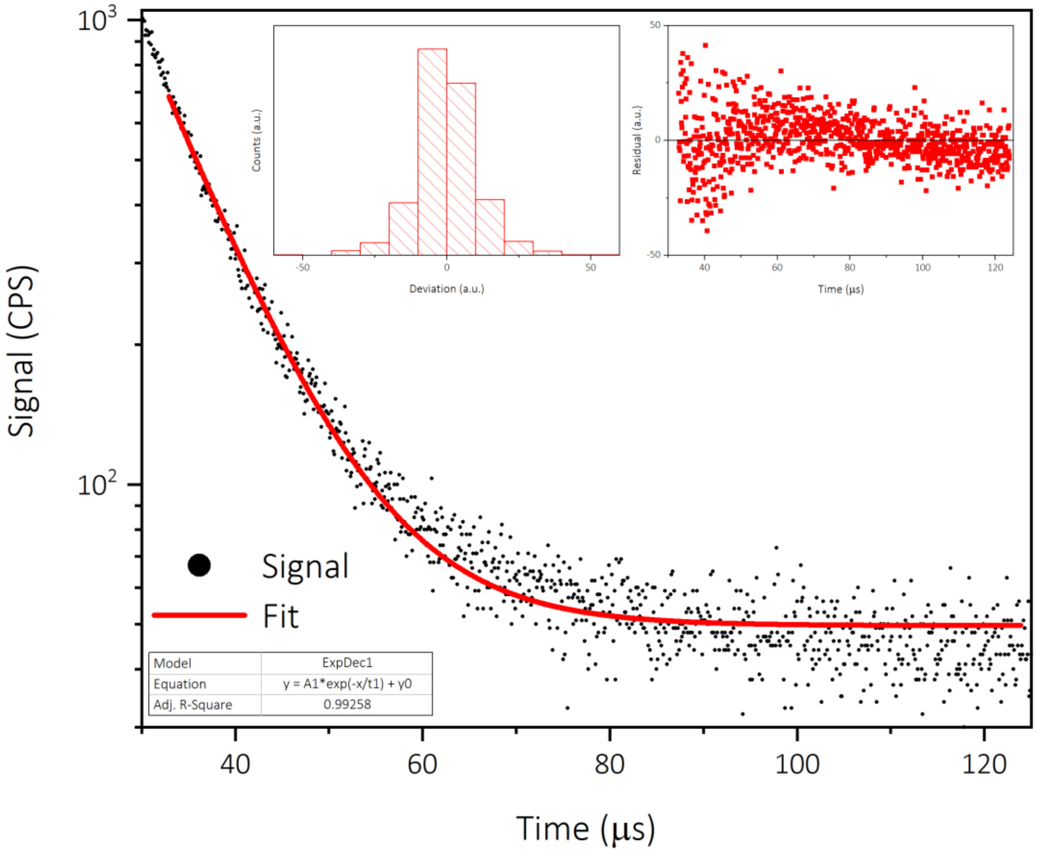

Figure S2.12: Solid-state luminescence decay profile (black) of the Yb^{III} signal in $[Yb_2Tb]$ with corresponding mono-exponential decay fit (red) (λ_{ex} = 280 nm, λ_{em} = 980 nm T = 20 K). Inset: associated residuals of the fit showing both normal distribution (left) and expected error variance (right).

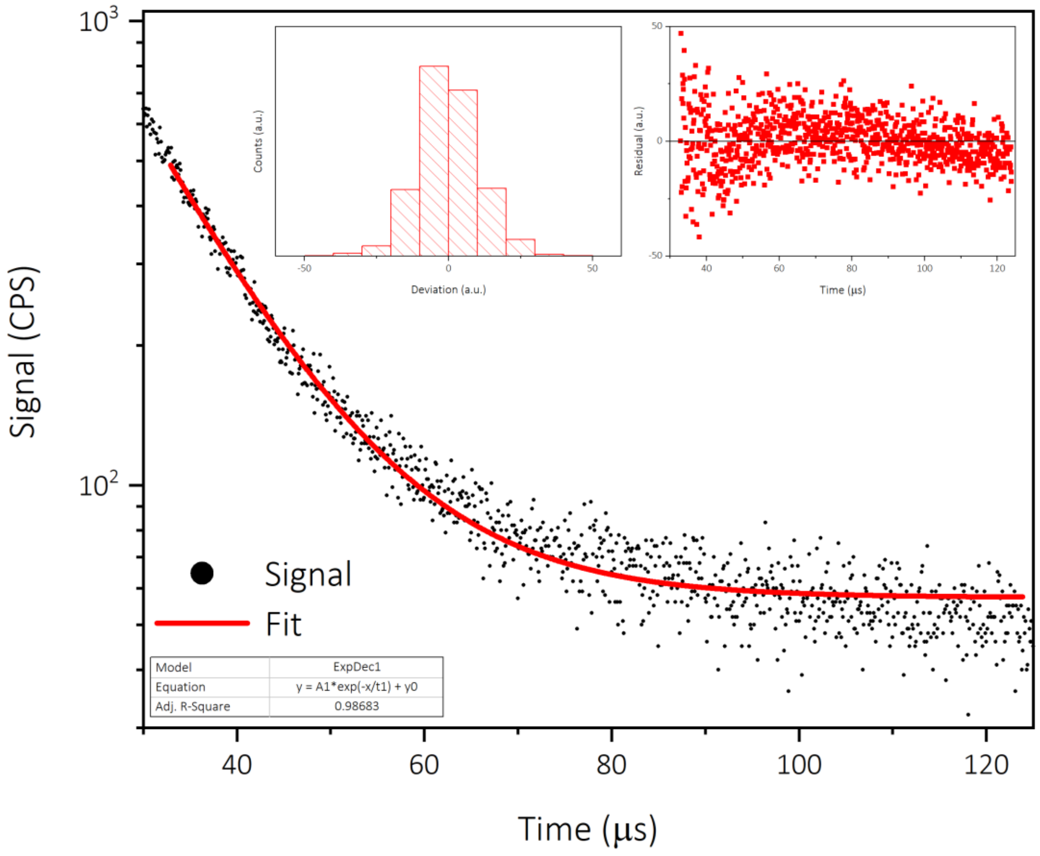

Figure S2.13: Solid-state luminescence decay profile (black) of the Yb^{III} signal in [Yb_2Tb] with corresponding mono-exponential decay fit (red) (λ_{ex} = 366 nm, λ_{em} = 980 nm T = 20 K). Inset: associated residuals of the fit showing both normal distribution (left) and expected error variance (right).

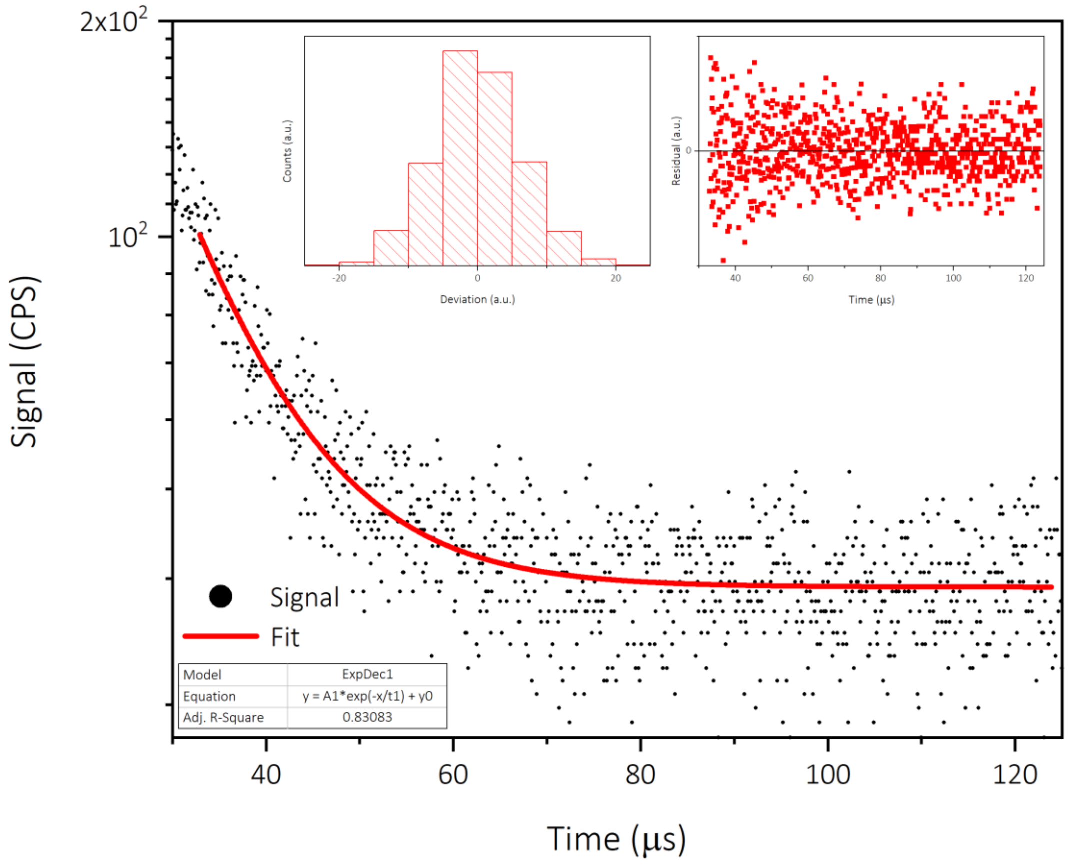

Figure S2.14: Solid-state luminescence decay profile (black) of the Yb^{III} signal in $[Yb_2Tb]$ with corresponding mono-exponential decay fit (red) (λ_{ex} = 488 nm, λ_{em} = 980 nm T = 20 K). Inset: associated residuals of the fit showing both normal distribution (left) and expected error variance (right).

2.3 [Eu_2Tb] spectra

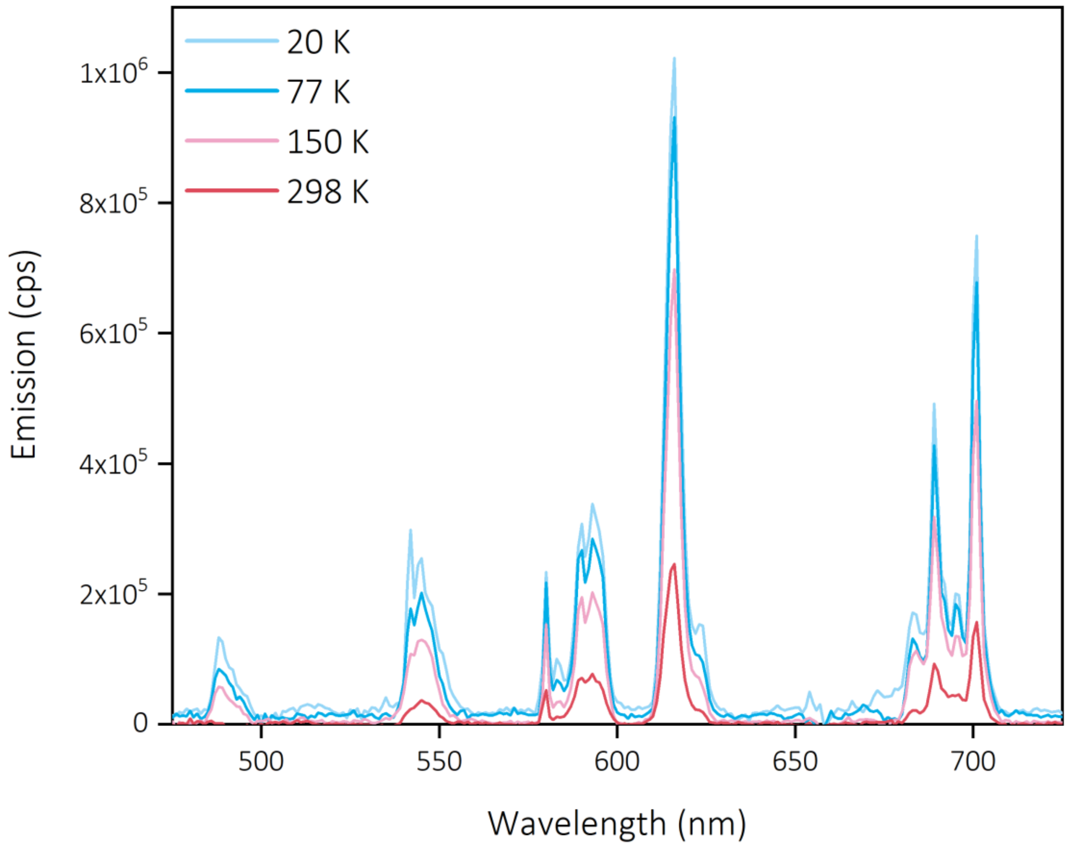

Figure S2.15: Solid-state variable temperature emission spectra of Eu^{III} and Tb^{III} in [Eu_2Tb] at 20 K (light blue), 77 K (dark blue), 150 K (light red) and 298 K (dark red) (λ_{ex} = 366 nm).

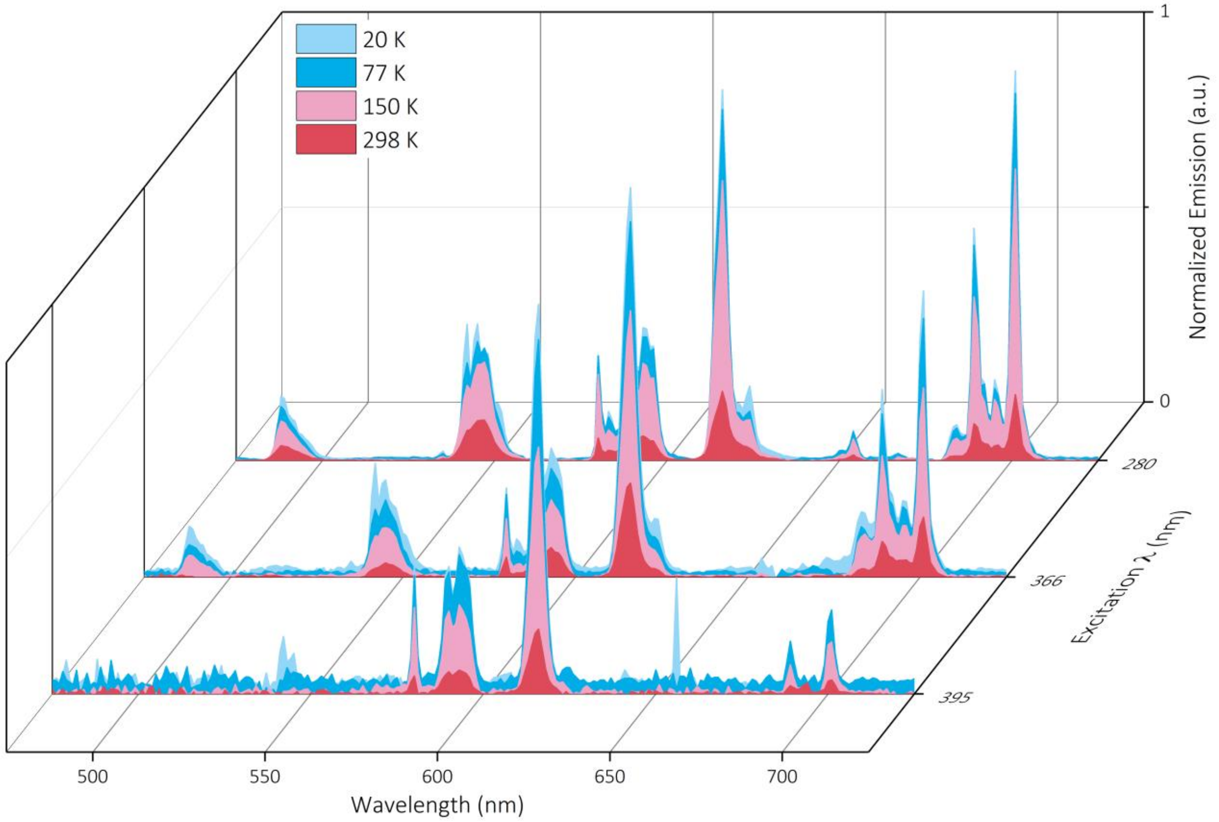

Figure S2.16: Normalized solid-state emission spectra of [Eu_2Tb] at 20 K (light blue), 77 K (dark blue), 150 K (light red) and 298 K (dark red) across multiple excitation wavelengths. Data are normalized at each λ_{ex} relative to the emission maximum (~615 nm @ T = 20 K).

Table S3: Comparison of solid-state average lifetime τ_{avg} and long-lived τ_2 component for Tb^{III} centers in both [Yb_2Tb] and [Eu_2Tb] trinuclear bimetallics across multiple temperatures (298 K: dark red, 150 K: light red, 77 K: dark blue, 20 K: light blue). Data is averaged across multiple λ_{ex} for clarity due to the negligible effect on lifetime values.

**Lifetime**	**Tb^{III} τ_{avg} (ms)**		**Tb^{III} τ_2 (ms)**	
**Complex**	[Yb_2Tb]	[Eu_2Tb]	[Yb_2Tb]	[Eu_2Tb]
**Temperature (K)** **298**	0.56	0.23	0.66	0.47
**150**	0.57	0.35	0.71	0.59
**77**	0.56	0.34	0.72	0.58
**20**	0.51	0.38	0.71	0.61

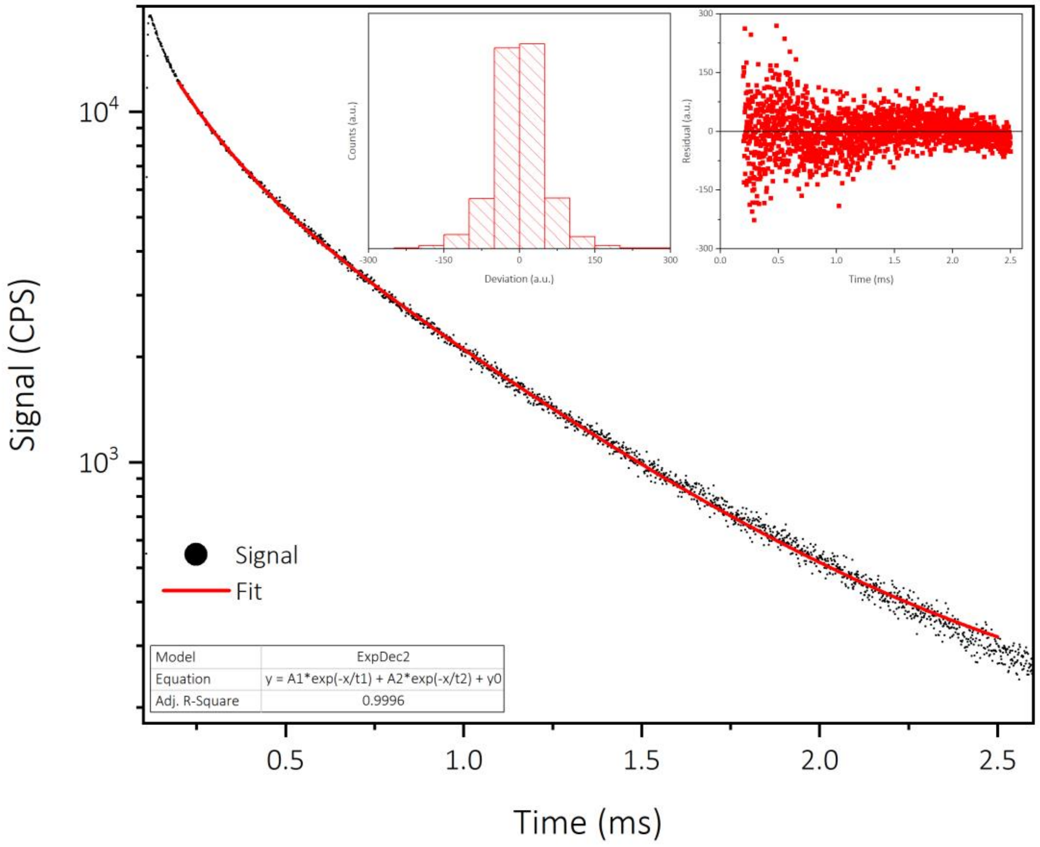

Figure S2.17: Solid-state luminescence decay profile (black) of the Eu^{III} signal in [Eu_2Tb] with corresponding bi-exponential decay fit (red) (λ_{ex} = 280 nm, λ_{em} = 615 nm T = 20 K). Inset: associated residuals of the fit showing both normal distribution (left) and expected error variance (right).

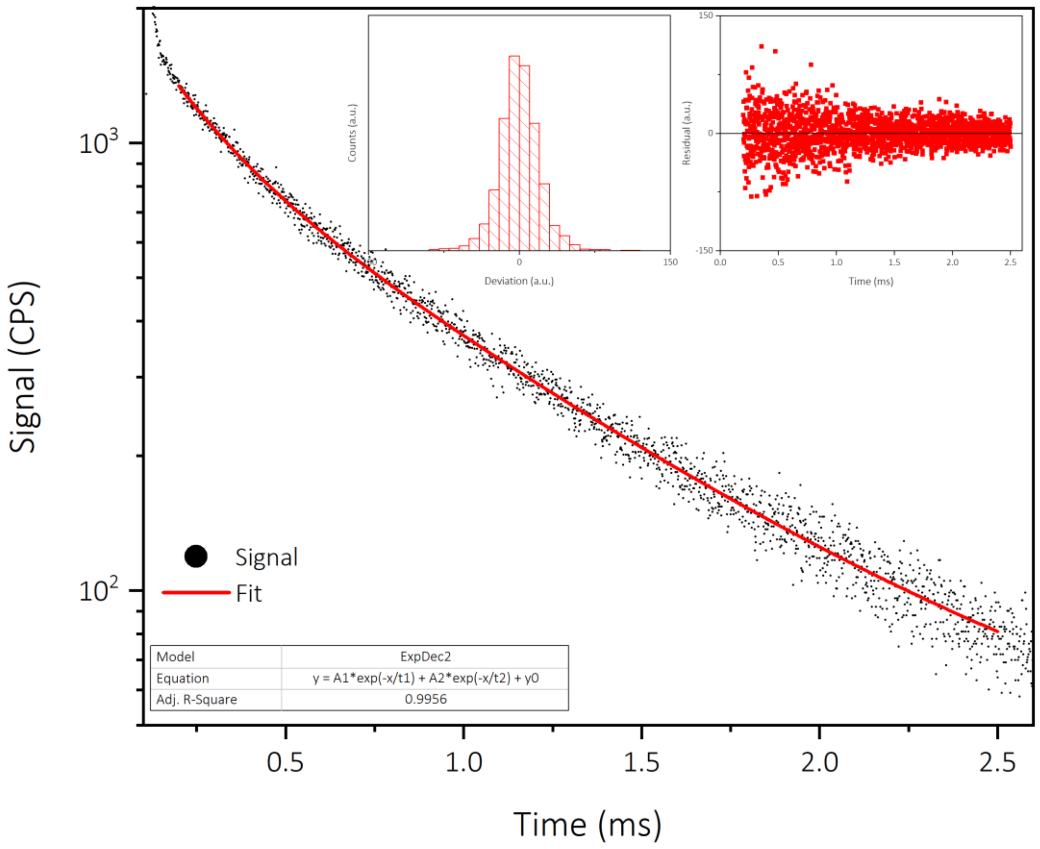

Figure S2.18: Solid-state luminescence decay profile (black) of the Eu^{III} signal in [Eu_2Tb] with corresponding bi-exponential decay fit (red) (λ_{ex} = 366 nm, λ_{em} = 615 nm T = 20 K). Inset: associated residuals of the fit showing both normal distribution (left) and expected error variance (right).

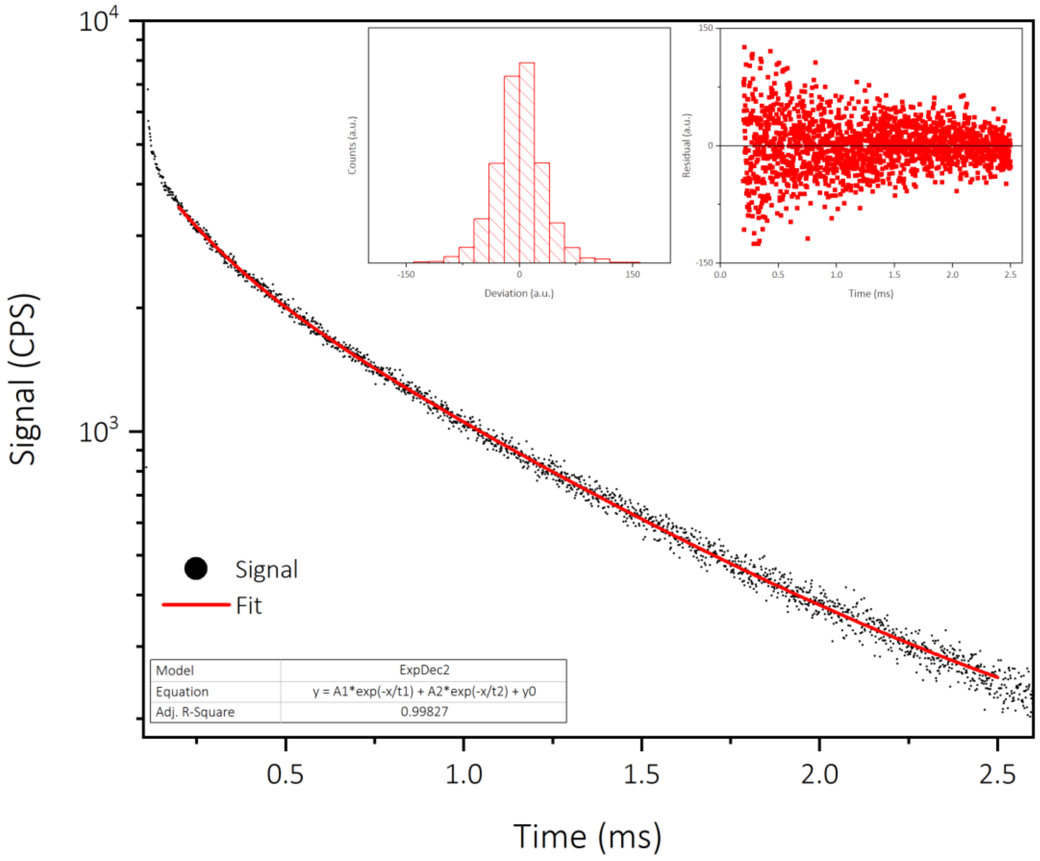

Figure S2.19: Solid-state luminescence decay profile (black) of the Eu^{III} signal in [Eu_2Tb] with corresponding bi-exponential decay fit (red) (λ_{ex} = 395 nm, λ_{em} = 615 nm T = 20 K). Inset: associated residuals of the fit showing both normal distribution (left) and expected error variance (right).

Table S4: List of individual decay components τ_n and their relative contribution (%) towards a bi-exponential decay in solid-state [Eu_2Tb] lifetimes (Eu^{III} λ_{em} = 615 nm), across variable excitation and temperature ranges. Percentages are color-scaled from high (green) to low (red) and highlight an even distribution between both components, with a notable dominance of τ_1 at higher temperatures.

**Eu^{III} lifetimes (ms)**		**20 K**		**77 K**		**150 K**		**298 K**	
		τ_1	τ_2	τ_1	τ_2	τ_1	τ_2	τ_1	τ_2
**λ_{ex} (nm)**	**280**	0.09	0.41	0.09	0.4	0.086	0.39	0.077	0.31
		49%	51%	50%	50%	53%	47%	70%	30%
	**366**	0.086	0.39	0.085	0.38	0.084	0.38	0.076	0.31
		51%	49%	52%	48%	54%	46%	65%	35%
	**395**	0.085	0.39	0.077	0.36	0.078	0.35	0.072	0.3
		55%	45%	56%	44%	57%	43%	66%	34%

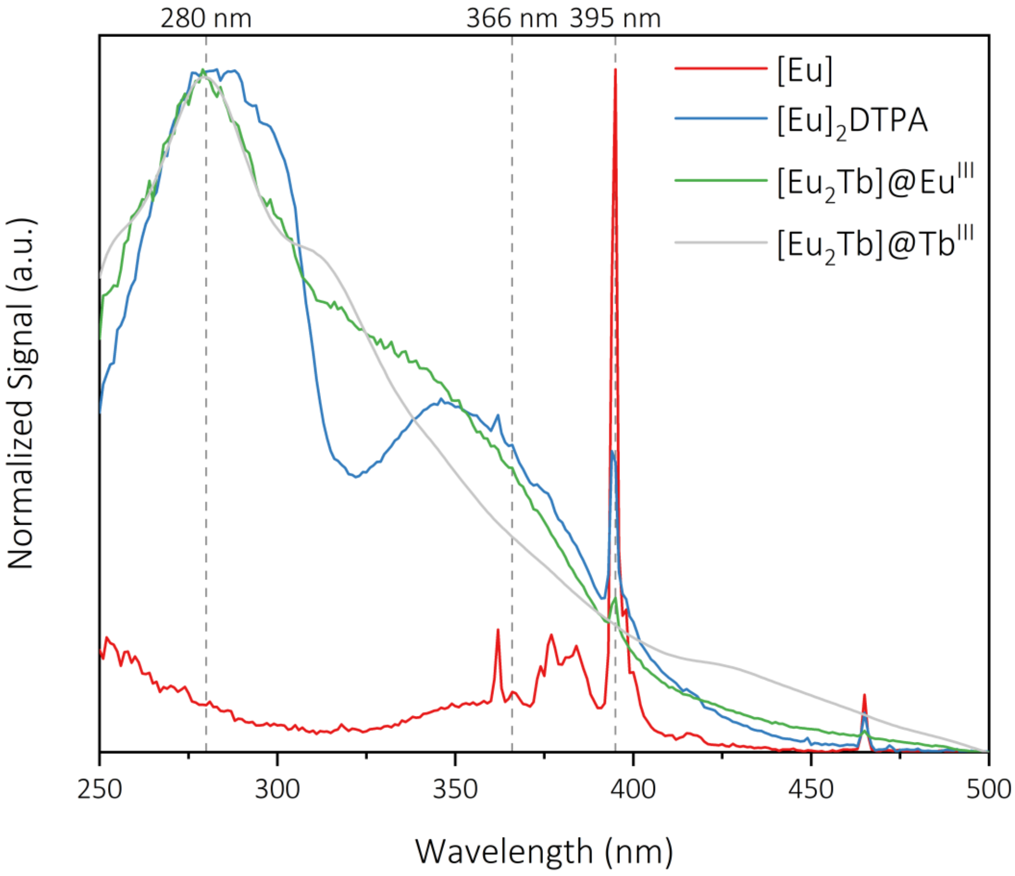

Figure S2.20: Normalized solid-state excitation spectra of Eu^{III} complexes [Eu] (red), $[Eu]_2DTPA$ (blue) and target heterometallic complex $[Eu_2Tb]$ (green) at 298 K, λ_{em} = 615 nm. The excitation trace of Tb^{III} from $[Eu_2Tb]$ (λ_{em} = 545 nm, grey) is included for comparison.

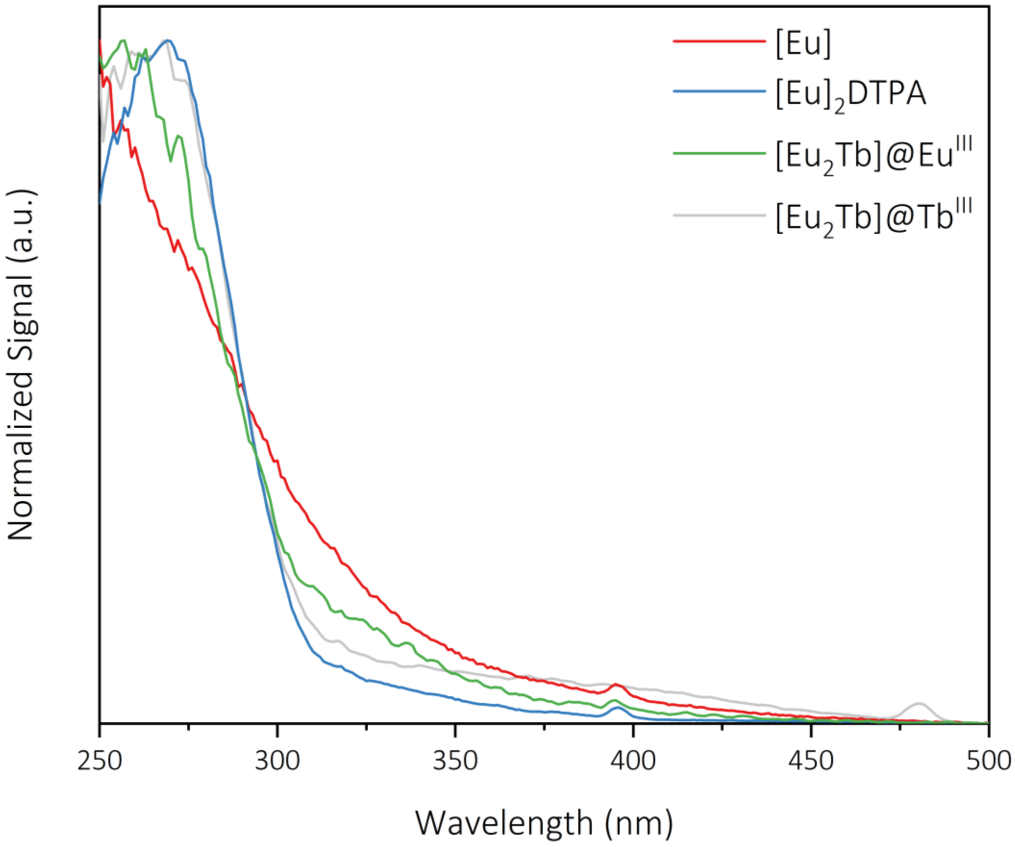

Figure S2.21: Normalized solution-state excitation spectra of Eu^{III} complexes [Eu] (red), $[Eu]_2DTPA$ (blue) and target trinuclear bimetallic $[Eu_2Tb]$ (green).

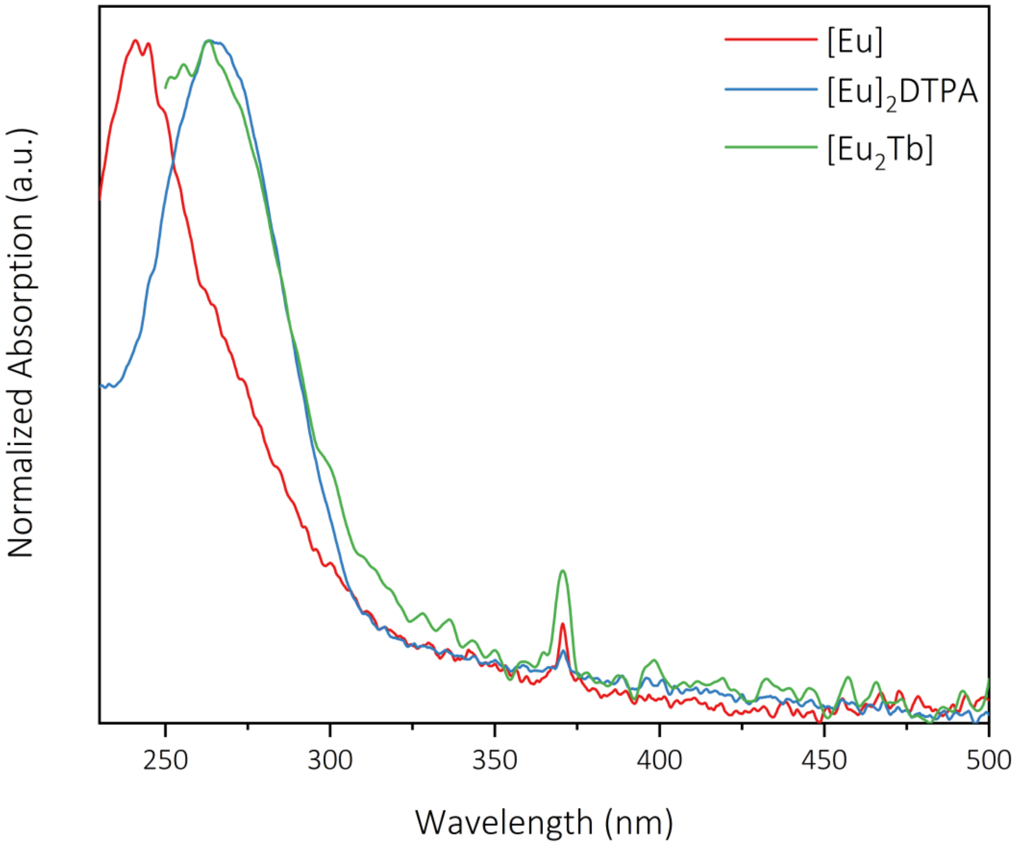

Figure S2.22: UV-VIS absorption spectra of Eu^{III} complexes [Eu] (red), $[Eu]_2DTPA$ (blue) and target heterometallic $[Eu_2Tb]$ (green) recorded in D_2O, A = 0.1.

2.4 [Yb_2Eu] spectra

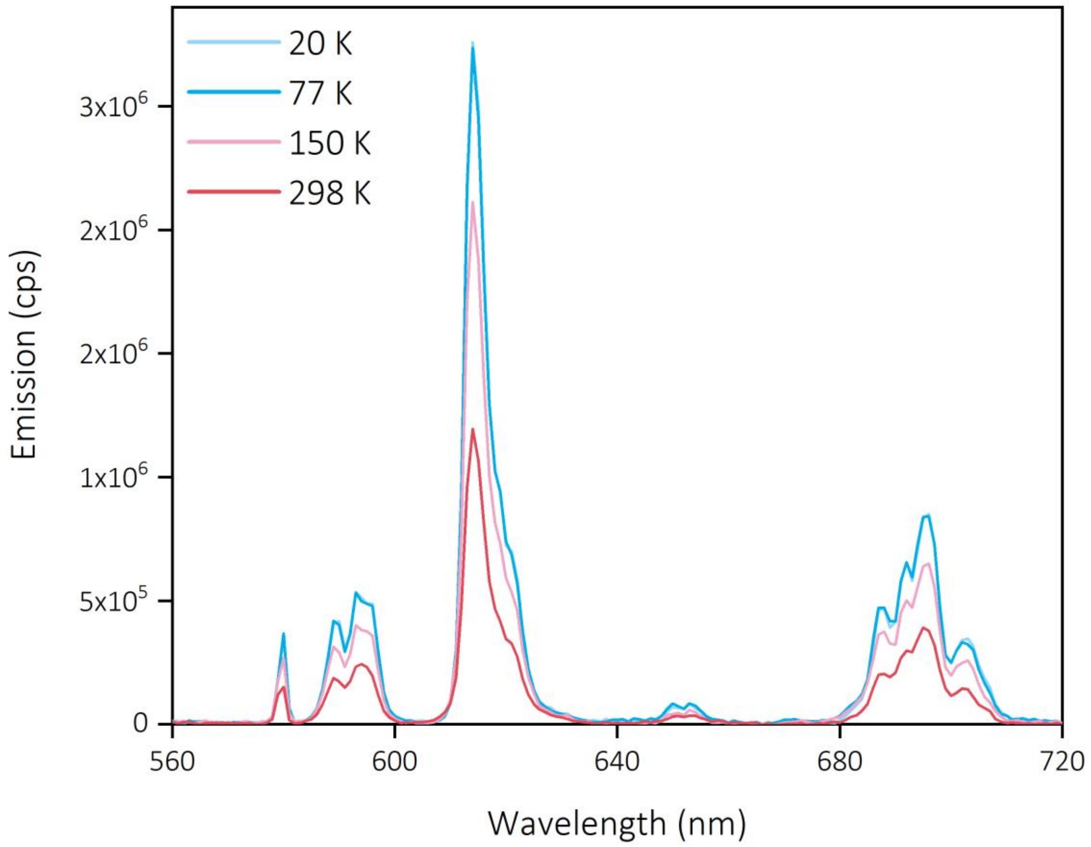

Figure S2.23: Solid-state variable temperature emission spectra of Eu^{III} in [Yb_2Eu] at 20 K (light blue), 77 K (dark blue), 150 K (light red) and 298 K (dark red) (λ_{ex} = 366 nm).

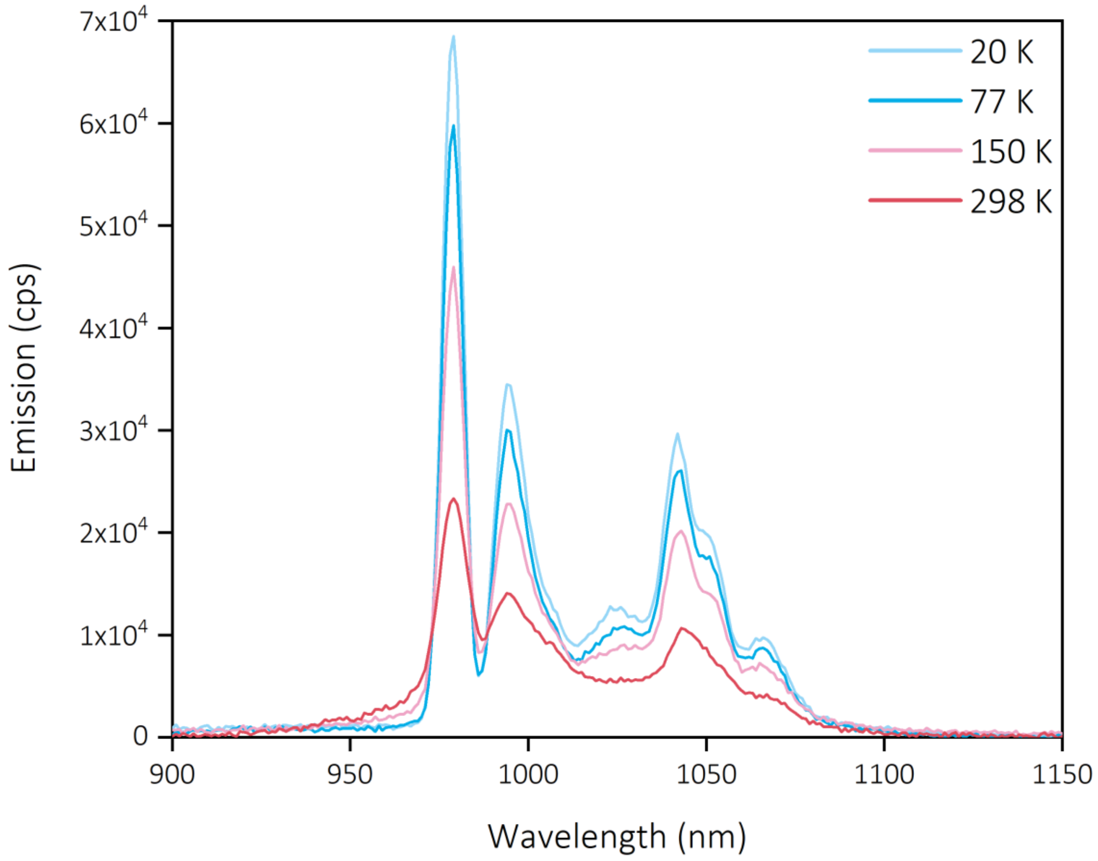

Figure S2.24: Solid-state variable temperature emission spectra of Yb^{III} in [Yb_2Eu] at 20 K (light blue), 77 K (dark blue), 150 K (light red) and 298 K (dark red) (λ_{ex} = 366 nm).

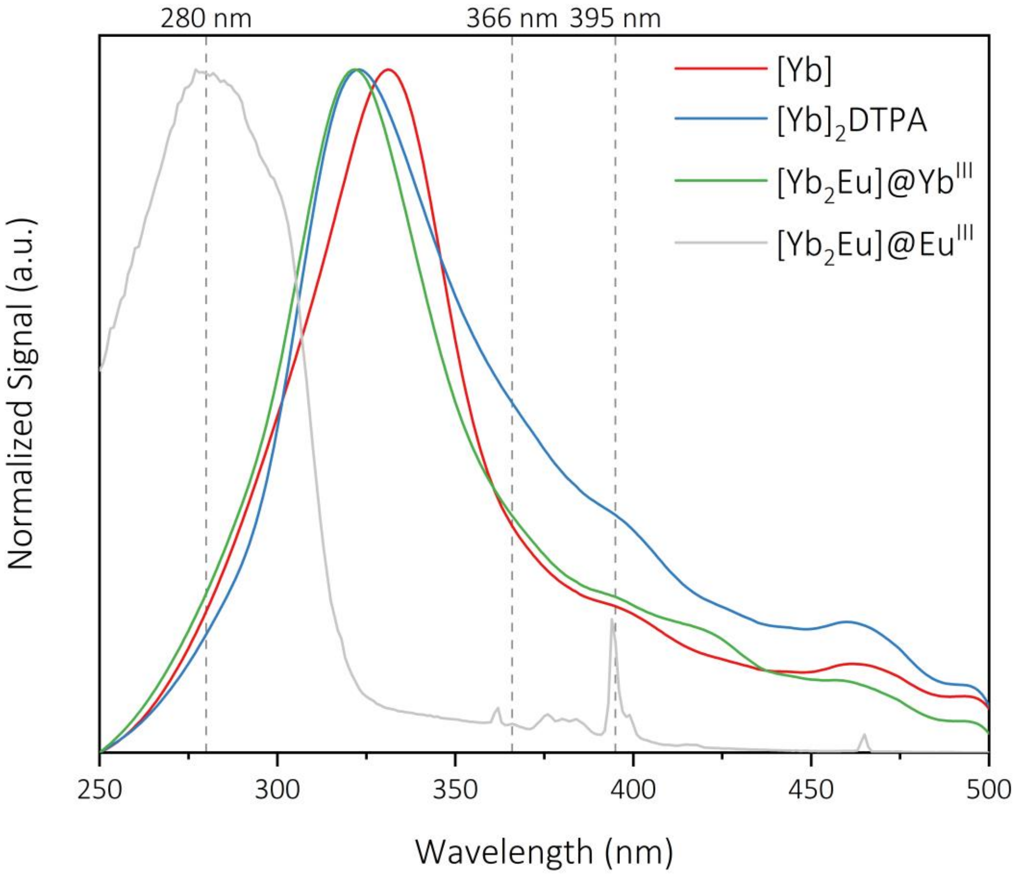

Figure S2.25: Normalized solid-state excitation spectra of Yb^{III} complexes [Yb] (red), $[Yb]_2DTPA$ (blue) and target heterometallic $[Yb_2Eu]$ (green) at 298 K, λ_{em} = 980 nm. The excitation trace of Eu^{III} from $[Yb_2Eu]$ (λ_{em} = 615 nm, grey) is included for comparison and shows distinct f-f bands.

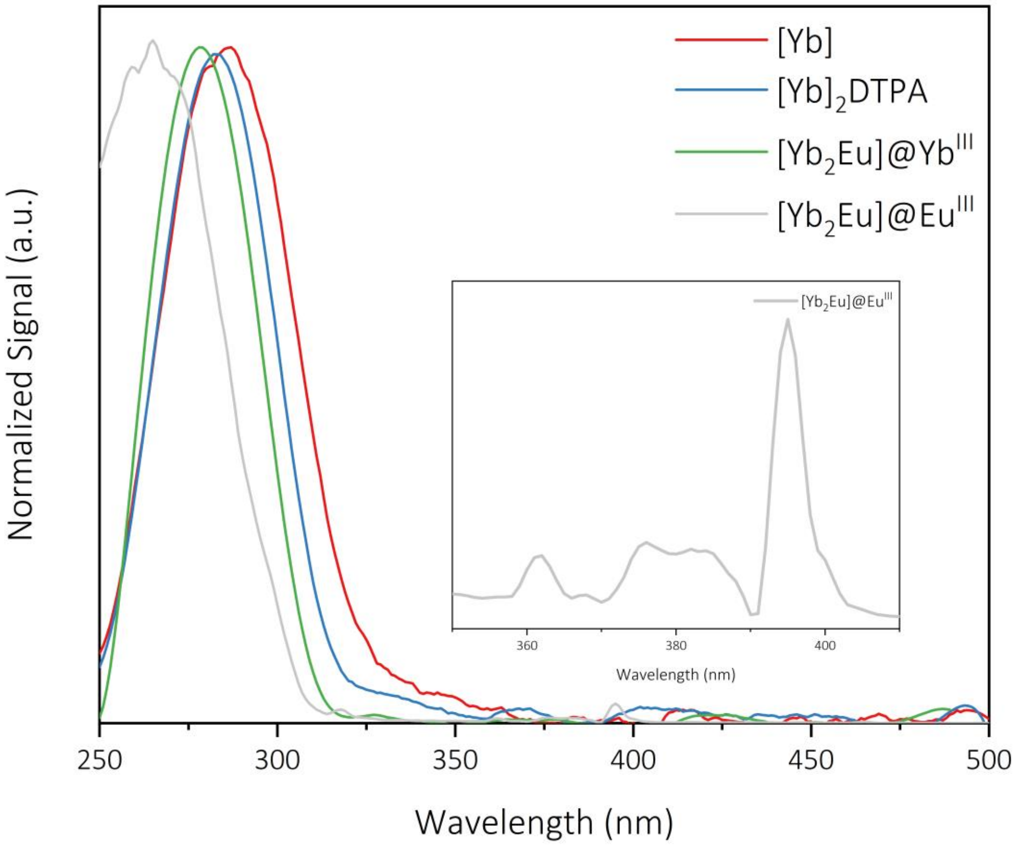

Figure S2.26: Normalized solution-state excitation spectra of Yb^{III} complexes [Yb] (red), [Yb]$_2$DTPA (blue) and target trinuclear bimetallic [Yb$_2$Eu] (green), also with f-f transitions present for Eu^{III} excitation (inset).

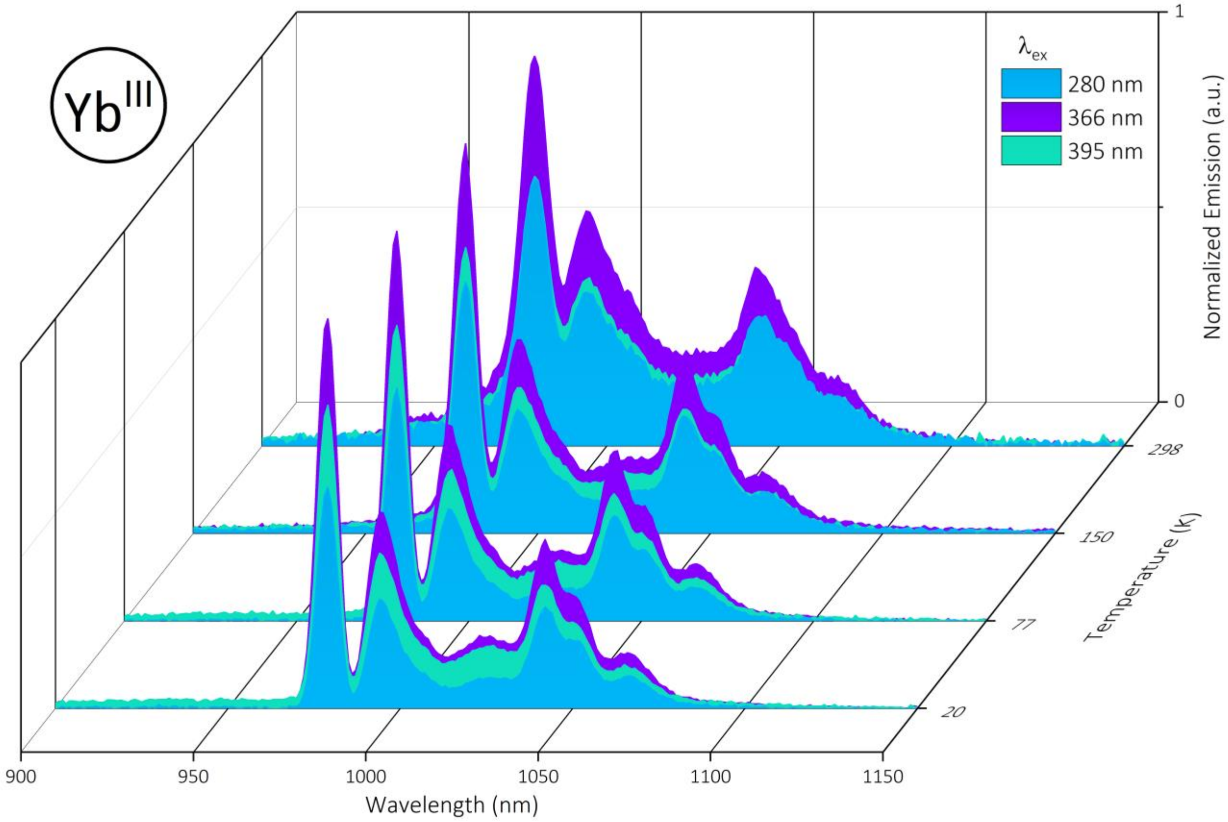

Figure S2.27: Normalized solid-state NIR emission spectra of [Yb_2Tb] at λ_{ex} = 280 nm (light blue), 366 nm (purple) and 395 nm (green) across a variable temperature range. Data are normalized at each temperature relative to the emission maximum (~980 nm @ λ_{ex} = 366 nm).

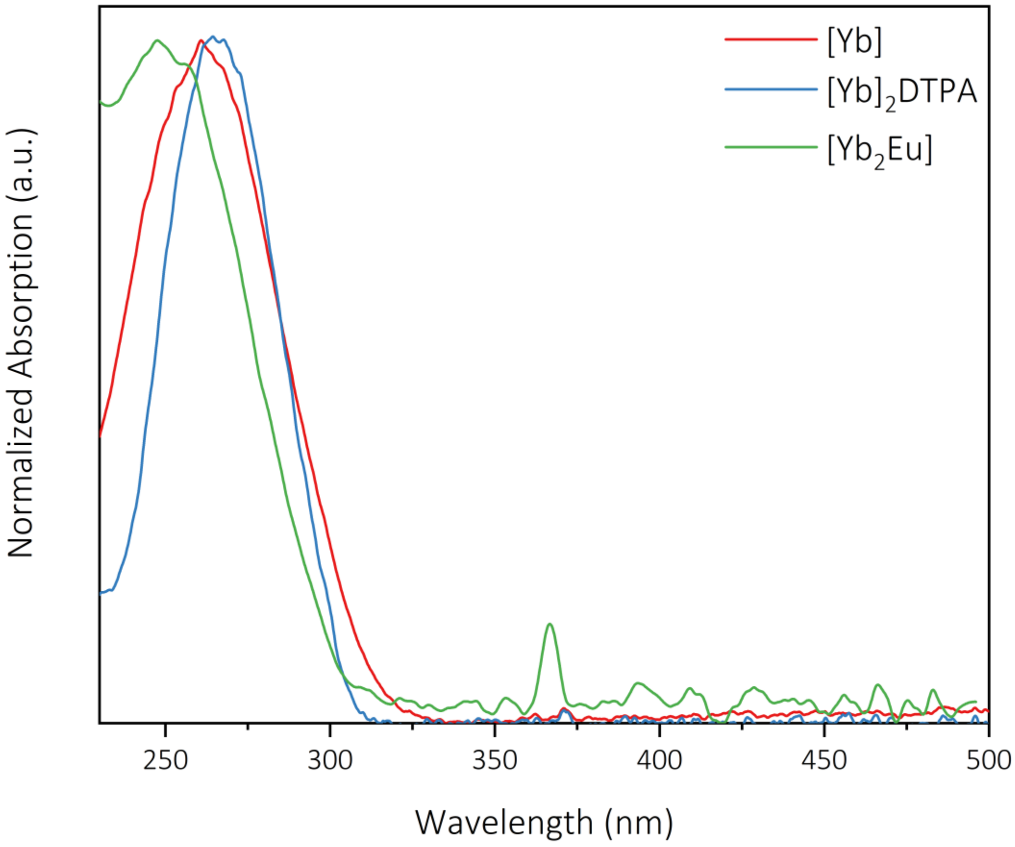

Figure S2.28: UV-VIS absorption spectra of Yb^{III} complexes [Yb] (red), $[Yb]_2DTPA$ (blue) and target trinuclear bimetallic $[Yb_2Eu]$ (green) recorded in D_2O, A = 0.1.

Table S5: List of individual decay components τ_n and their relative contribution (%) towards a bi-exponential decay in solid-state $[Yb_2Eu]$ lifetimes (Eu^{III} λ_{em} = 615 nm), across variable excitation and temperature ranges. Percentages are color-scaled from high (green) to low (red) and highlight the dominance of the long-lived τ_2 component throughout.

**Eu^{III} lifetimes (ms)**		**20 K**		**77 K**		**150 K**		**298 K**	
		τ_1	τ_2	τ_1	τ_2	τ_1	τ_2	τ_1	τ_2
**λ_{ex} (nm)**	**280**	0.15	0.59	0.17	0.67	0.16	0.66	0.16	0.67
		29%	71%	21%	79%	22%	78%	21%	79%
	**366**	0.19	0.79	0.19	0.77	0.23	0.82	0.15	0.69
		18%	82%	16%	84%	21%	79%	21%	79%
	**395**	0.17	0.81	0.2	0.85	0.17	0.79	0.16	0.75
		16%	84%	16%	84%	16%	84%	18%	82%

2.5 Supporting figures

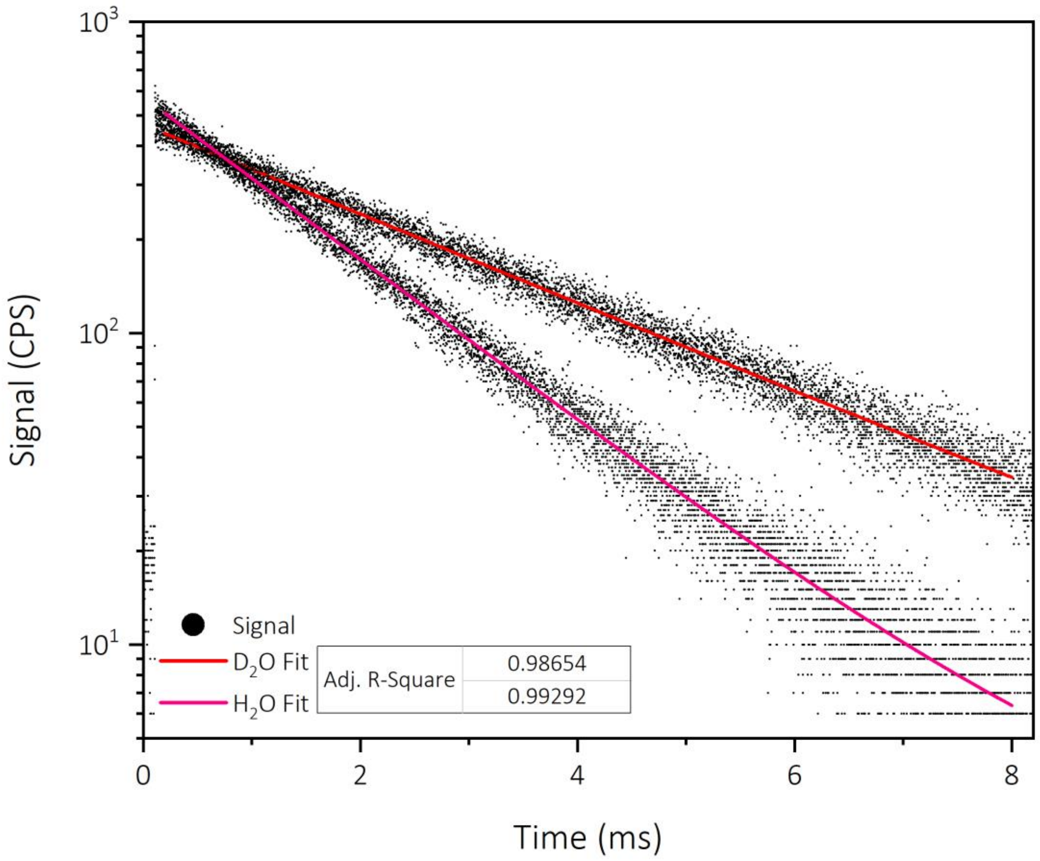

Figure S2.29: Solution-state luminescence decay profiles (black) of Tb^{III} in [Yb_2Tb] in deuterated and non-deuterated solvent. Corresponding mono-exponential decay fits are shown for both D_2O (red) and H_2O (pink) (λ_{ex} = 280 nm, λ_{em} = 545 nm T = 298 K).

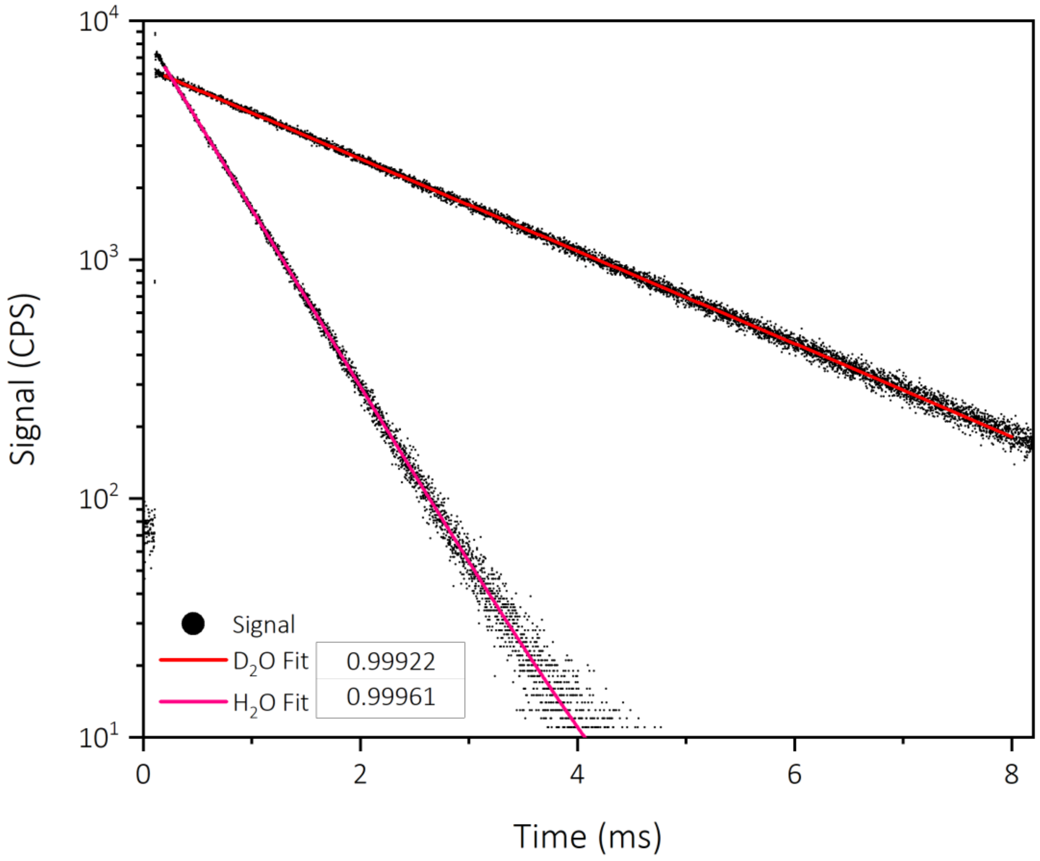

Figure S2.30: Solution-state luminescence decay profiles (black) of Eu^{III} in [Yb_2Eu] in deuterated and non-deuterated solvent. Corresponding mono-exponential decay fits are shown for both D_2O (red) and H_2O (pink) (λ_{ex} = 280 nm, λ_{em} = 615 nm T = 298 K).

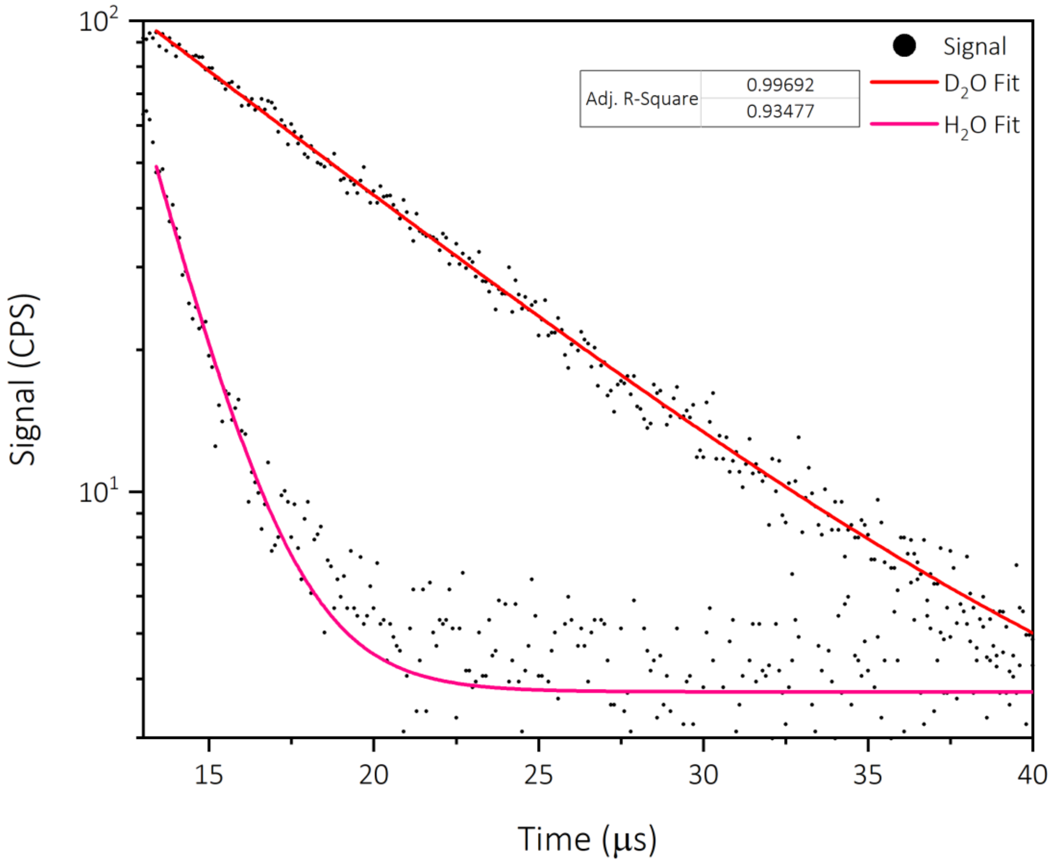

Figure S2.31: Solution-state luminescence decay profiles (black) of Yb^{III} in [Yb$_2$Eu] in deuterated and non-deuterated solvent. Corresponding mono-exponential decay fits are shown for both D_2O (red) and H_2O (pink) (λ_{ex} = 280 nm, λ_{em} = 980 nm T = 298 K).

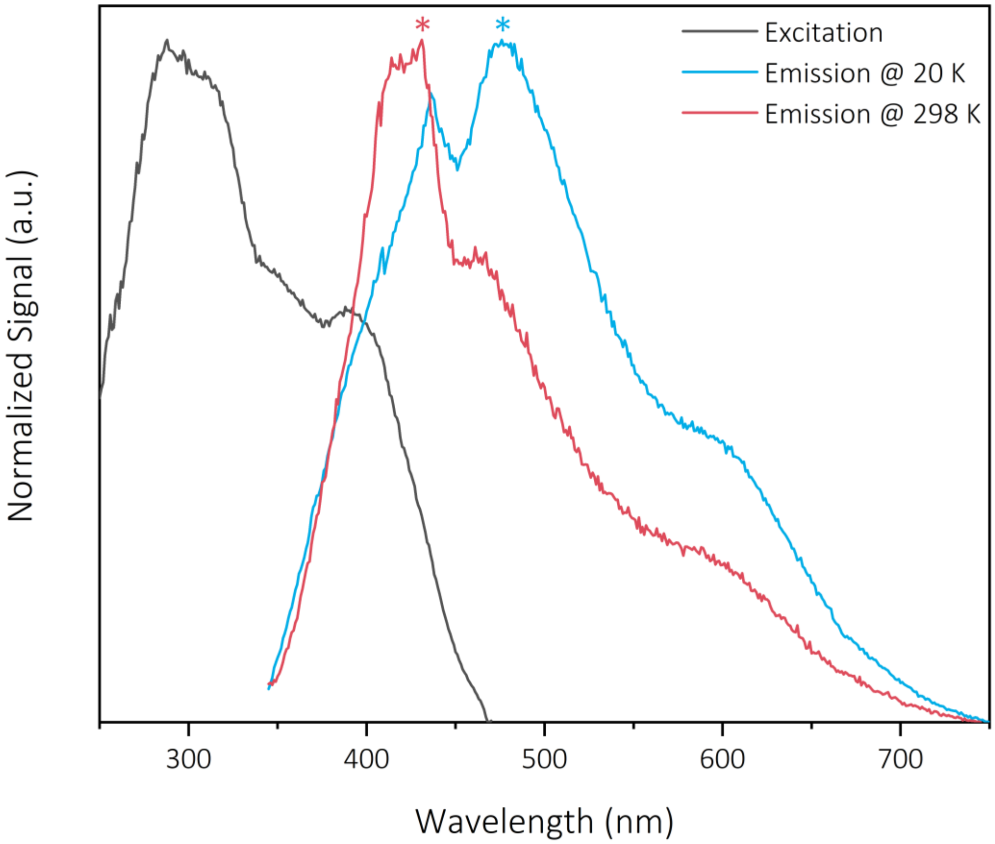

Figure S2.32: Normalized solid-state excitation (λ_{em} = 480 nm, grey) and variable temperature emission (λ_{ex} = 300 nm) spectra of [Gd]. Emission at 298 K (red) and 20 K (blue) imply relative positions of the ligand S_1 and T_1 states.

Table S6: Solid-state lifetimes for 'silent partner' [Ln_2Lu] complexes, compared with analogous compounds from the [Ln_2Ln'] complex series. Eu^{III} values are calculated average lifetimes from a bi-exponential fit.

Lifetime		Yb^{III} τ (μs)			Eu^{III} τ_{avg} (ms)		
**Complex**		[Yb_2Tb]	[Yb_2Eu]	[Yb_2Lu]	[Eu_2Tb]	[Yb_2Eu]	[Eu_2Lu]
**Temperature (K)**	**298**	8.1	9.1	8.4	0.15	0.60	0.70
	**150**	8.4	9.7	8.6	0.21	0.65	0.82
	**77**	8.7	10	8.7	0.23	0.66	0.84
	**20**	9.2	11	9.0	0.24	0.62	0.83
**Global τ**		**8.6** ± *0.4*	**9.8** ± *0.6*	**8.7** ± *0.3*	**0.21** ± *0.02*	**0.63** ± *0.09*	**0.80** ± *0.06*

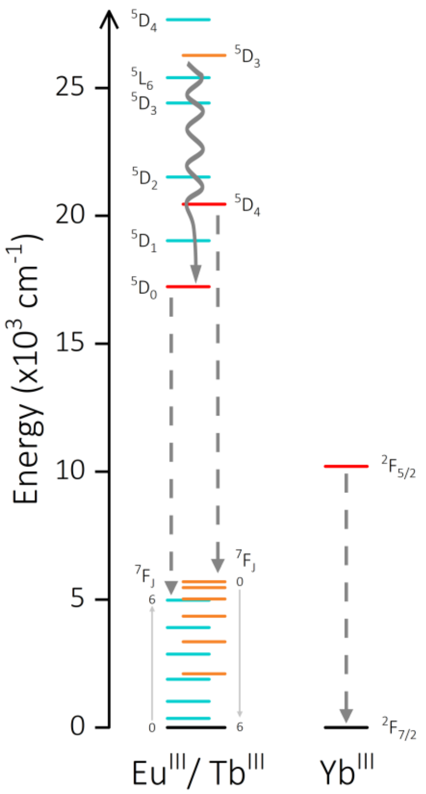

Figure S2.33: Energy level diagram showing the high degree of energetic overlap in ^5D_J and ^7F_J states of Eu^{III} (green) and Tb^{III} (orange). Primary emissive states are highlighted red. Waved arrow: non-radiative decay, dashed arrow: emission.

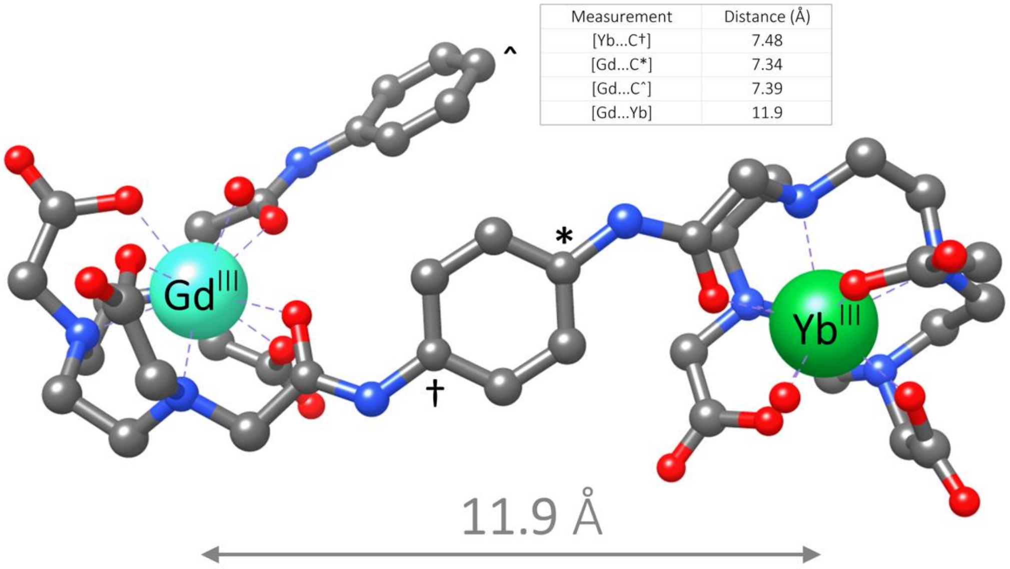

Figure S2.34: Proposed intermetallic distance in [Ln_2Ln'] calculated from modified and energy minimized crystal structures of Gd^{III}(DTPA) (CCDC ID: QEZGIM) and Yb^{III}(DO3A) (CCDC ID: EGOWUV) complexes (Dutta *et al*., 2006; Pujales-Paradela *et al*., 2019). Tabulated Ln^{III}…C distances are measured directly from CCDC data. Energy minimization and distance measurements carried out in Avogadro (UFF force field, steepest descent) and visualized using Chimera.

3 References

Beeby, A., Clarkson, I. M., Dickins, R. S., Faulkner, S., Parker, D., Royle, L., et al. (1999). Non-radiative deactivation of the excited states of europium, terbium and ytterbium complexes by proximate energy-matched OH, NH and CH oscillators: an improved luminescence method for establishing solution hydration states. *J. Chem. Soc., Perkin Trans., 2*, 493-503. doi: 10.1039/a808692c

Dutta, S., Kim, S. K., Eun, J. L., Kim, T. J., Kang, D. S., Chang, Y., et al. (2006). Synthesis and Magnetic Relaxation Properties of Paramagnetic Gd-complexes of New DTPA-bis-amides. The X-ray Crystal Structure of $[Gd(L)(H_2O)]\cdot 3H_2O$ (L = DTPA-bis(4-carboxylicphenyl)amide). *Bull. Korean Chem. Soc.*, 27, 1038–1042. doi: 10.5012/bkcs.2006.27.7.1038.

Natrajan, L. S., Villaraza, A. J. L., Kenwright, A. M., and Faulkner, S. (2009). Controlled preparation of a heterometallic lanthanide complex containing different lanthanides in symmetrical binding pockets. *Chem. Commun.* 40, 6020–6022. doi: 10.1039/b913702e.

Pujales-Paradela, R., Savić, T., Pérez-Lourido, P., Esteban-Gómez, D., Angelovski, G., Botta, M., et al. (2019). Lanthanide Complexes with ^{1}H paraCEST and ^{19}F Response for Magnetic Resonance Imaging Applications. *Inorg. Chem.*, 58, 7571–7583. doi: 10.1021/acs.inorgchem.9b00869.
